# Distinct neural activation patterns of age in subcomponents of inhibitory control: A fMRI meta-analysis

**DOI:** 10.3389/fnagi.2022.938789

**Published:** 2022-08-05

**Authors:** Jixin Long, Xiaoqi Song, You Wang, Chanyu Wang, Ruiwang Huang, Ruibin Zhang

**Affiliations:** ^1^Cognitive Control and Brain Healthy Laboratory, Department of Psychology, School of Public Health, Southern Medical University, Guangzhou, China; ^2^Department of Psychiatry, Zhujiang Hospital, Southern Medical University, Guangzhou, China; ^3^School of Psychology, South China Normal University, Guangzhou, China

**Keywords:** inhibitory control, response inhibition, cognitive inhibition, fMRI, aging

## Abstract

Inhibitory control (IC) is a fundamental cognitive function showing age-related change across the healthy lifespan. Since different cognitive resources are needed in the two subcomponents of IC (cognitive inhibition and response inhibition), regions of the brain are differentially activated. In this study, we aimed to determine whether there is a distinct age-related activation pattern in these two subcomponents. A total of 278 fMRI articles were included in the current analysis. Multilevel kernel density analysis was used to provide data on brain activation under each subcomponent of IC. Contrast analyses were conducted to capture the distinct activated brain regions for the two subcomponents, whereas meta-regression analyses were performed to identify brain regions with distinct age-related activation patterns in the two subcomponents of IC. The results showed that the right inferior frontal gyrus and the bilateral insula were activated during the two IC subcomponents. Contrast analyses revealed stronger activation in the superior parietal lobule during cognitive inhibition, whereas stronger activation during response inhibition was observed primarily in the right inferior frontal gyrus, bilateral insula, and angular gyrus. Furthermore, regression analyses showed that activation of the left anterior cingulate cortex, left inferior frontal gyrus, bilateral insula, and left superior parietal lobule increased and decreased with age during cognitive inhibition and response inhibition, respectively. The results showed distinct activation patterns of aging for the two subcomponents of IC, which may be related to the differential cognitive resources recruited. These findings may help to enhance knowledge of age-related changes in the activation patterns of IC.

## Introduction

Inhibitory control refers to the ability to suppress unwanted actions that are not appropriate for the current situation or to resist distractions and adapt to conflicting situations (Goldman-Rakic, 1996; Aron, 2007; Aïte et al., 2018). By doing so, humans can selectively attend to task-relevant information and engage in goal-directed rather than habitual actions, as well as staying away from dangerous environments. Dysfunctional inhibitory control is considered to be one of the symptoms of various disorders, including affective and anxiety disorders (Paus et al., 2008), eating disorders (Bartholdy et al., 2019), learning difficulties (Eickhoff et al., 2008), and substance abuse (Steele et al., 2018). More importantly, it is thought to be an essential cause of cognitive function decline in the aging brain (Zacks et al., 2000). Better understanding of the developmental trajectory of inhibitory control and its neural correlates across the healthy lifespan could not only help to identify the pivotal time point at which inhibitory control and related neural networks decline over the lifespan but will also help to formulate an early intervention strategy for people who are at risk of inhibitory control deficits.

### Behavioral and neural developmental trajectory of inhibitory control

In the recent years, there has been an increase in the number of studies examining the behavioral and neural developmental trajectory of inhibitory control across the lifespan (refer to Supplementary Figure 1). However, there is a lack of agreement in the results of existing studies examining the behavioral developmental trajectory of inhibitory control. For example, some studies have suggested a steady increase in inhibitory control from adolescence to adulthood (Velanova et al., 2008; Aïte et al., 2018), and some studies revealed a lack of improvement in inhibitory control from adolescence to adulthood (Luna et al., 2004; Ordaz et al., 2013; Humphrey and Dumontheil, 2016), while other studies have found that inhibitory control develops until adolescence and then declines slightly from young adulthood to old age (Schachar and Logan, 1990; Williams et al., 1999).

In neuroimaging research examining the neural developmental trajectory of inhibitory control, the results are also not consistent. Increased frontal activation in adults compared with children has been observed in some fMRI studies (Bunge et al., 2002; Tamm et al., 2002; Luna et al., 2004). Rubia et al. (2013) reported that superior performance in adults was paralleled by increased activation in a network comprising prefrontal and parietal cortical regions in cognitive inhibition. Moreover, Vink et al. (2014) showed increased activation with age during response inhibition in the right inferior frontal cortex and supplementary motor area. Using a Go/NoGo task, Cope et al. (2020) reported a significant positive linear activation associated with age in the frontal, temporal, parietal, and occipital cortices, which means that activation in these regions during response inhibition increased with age. However, other neuroimaging studies have reported stronger activation during cognitive inhibition in the frontal and parietal lobes in children and adolescents aged 9–12 years compared with adults aged 20–30 years (Booth et al., 2003). Durston et al. (2002) reported stronger activation in the bilateral ventral prefrontal cortex, right parietal lobe, and right dorsolateral prefrontal cortex for children than adults during a response inhibition task.

Previous meta-analyses have also compared brain activation across different age groups to explore the age effect of brain activation during the process of inhibitory control (Nielson et al., 2004; Fernandez-Ruiz et al., 2018). For example, Nielson et al. (2004) reported that activation during inhibition occurred predominantly in the right prefrontal and parietal regions in participants older than the young adult group; in their study, the researchers compared data collected from four different age groups (18–31 years for young adults, 33–55 years for middle-aged adults, 62–72 years for young elderly, and 73–78 years for elderly). Moreover, Fernandez-Ruiz et al. (2018), in a study including individuals aged 18–25 years for young adults and 49–83 years for older adults, reported decreased activation in the anterior cingulate and increased activation in the dorsolateral prefrontal cortex in the older group but not in the younger group. The above studies, which had a lack of agreement on the developmental patterns of behavioral performance and neural activation during inhibitory control, suggest that the developmental patterns of inhibitory control with aging need to be further clarified.

The lack of agreement in the existing studies on the behavioral and neural developmental trajectory of inhibitory control across the lifespan may be due to studies including samples with limited age ranges or the fact that few existing studies or theories have systematically differentiated age-related changes in different inhibitory control tasks. For example, Aïte et al. (2018) recruited 160 participants aged 10–23 years to investigate the developmental patterns of inhibitory control and the degree of specificity of inhibitory control in children, adolescents, and adults (not including older adults), whereas Velanova et al. (2009) only recruited 98 individuals aged 8–27 years in their study. Similarly, Humphrey and Dumontheil (2016) only included 90 participants aged 12–18 years in their study, which revealed a lack of improvement in inhibitory control from adolescence to adulthood, and did not include younger children or older adults as participants. Furthermore, Ordaz et al. (2013) carried out a longitudinal study including a total of 123 individuals spanning the age range of 9–26 years.

Most current studies on the behavioral and neural developmental trajectory of inhibitory control across the lifespan include only a single type of inhibitory control task (Booth et al., 2003; Andrés et al., 2008; Anguera and Gazzaley, 2012). For example, Anguera and Gazzaley (2012) reported that in a stop signal task, the stop signal reaction time of older adults was slower than that observed in younger adults, suggesting an age-related deficit in inhibitory control in the older population, whereas Booth et al. (2003) reported that children had more errors and slower reaction times compared to adults in a selective attention task. Similarly, Andrés et al. (2008) found that aging affected the ability to cancel a strong response in a stop signal task but did not affect performance in a Stroop task. Notably, Hasher and Zacks (1988) suggested that working memory capacity, as a core component of executive function, was constrained by the resources demanded in different working memory tasks and declined across the adult lifespan. The above studies suggest that since inhibitory control is also a subcomponent of executive function (Miyake et al., 2000), and different developmental trajectories of inhibitory control across the lifespan may also be impacted by varying degrees of task demand (Sebastian et al., 2013a). Therefore, there is a critical need to clarify the similarities and differences between different inhibitory control tasks (Dalley et al., 2011; Swick et al., 2011; Sebastian et al., 2013b) and further explore the developmental trajectory of inhibitory control through a wider age range under the framework of different subcomponents of inhibitory control.

### Subcomponents of inhibitory control and its neural correlates

In the recent times, an increasing number of studies have not only focused on explaining the diversity, scope, and range of inhibitory control functions, but have explored the intrinsic variability of different components of inhibitory control. These studies have suggested that inhibitory control is not a unitary construct, but is the one which can be further differentiated into different subcomponents (Aron, 2011; Brevers et al., 2017; Gavazzi et al., 2021). For example, by capturing the temporal dynamics in the processes of inhibitory control, Braver (2012) described a dual mechanism of control (DMC) framework, which hypothesized that inhibitory control operates through two distinct modes—proactive control and reactive control—depending on the time that the action was withheld. Proactive inhibition is considered as a “top-down” model of inhibitory control, which actively maintains goal-relevant information and facilitates the suppression of the coming action before the occurrence of cognitive events (Braver, 2012). In contrast, reactive inhibition is a “bottom-up” form of control and is thought to be a stopping mechanism triggered by an already-initiated motor response (Aron, 2011; Meyer and Bucci, 2016). Experimental evidence from neuroimaging studies shows partially overlapping regions such as the frontoparietal circuit activated in the processes of both reactive and proactive inhibitions, which may reflect cognitive function or brain network sharing between these two inhibitory control processes (Zandbelt et al., 2011; van Belle et al., 2014). These studies suggest that dual mechanisms of control, namely, proactive inhibition and reactive inhibition, may not be sensitive to the distinct cognitive function developmental trajectory.

To differentiate the cognitive process during inhibitory control, inhibitory control can also be classified into two subcomponents—cognitive and response inhibitions—depending on the cognitive process of different inhibition targets in inhibitory control tasks (Hung et al., 2018). Cognitive inhibition involves the suppression of competing cognitive processing to solve relevant problems, whereas response inhibition involves the suppression of a prepotent response or an already-initiated action to perform a different, more context-appropriate response (Sebastian et al., 2013a; Kan et al., 2021). The dissociation of cognitive inhibition and response inhibition may provide useful information to further understand different manifestations of inhibitory dysfunction, which will greatly benefit clinical research.

Differences between inhibition difficulties and complexity of the two types of inhibition tasks might originate from the differences in cognitive resources recruited in these subcomponents of inhibitory control tasks (Sebastian et al., 2013a). Stahl et al. (2014) used a multicomponent modeling approach and showed that the control of response-related interference is not a unitary construct and that cognitive interference can be separated from response inhibition. Noreen and MacLeod (2015) showed no significant correlations or commonalities between different inhibition tasks such as Stroop, Go/NoGo, and stop signal tasks, suggesting that different inhibitory control tasks primarily assess different aspects of inhibition processes and involve different brain system or neural mechanisms. This may be attributed to the variety of inhibitory control tasks with different demands of cognitive resources, the latter of which may eventually result in the differences in inhibition behavioral performance with increasing age across the healthy lifespan.

Cognitive inhibition can be captured by paradigms including the Stroop, Flanker, and Simon and stimulus response compatibility (SRC) (Stahl et al., 2014; van Velzen et al., 2014; Almdahl et al., 2021). In the Stroop or Flanker tasks, participants are required to suppress interference due to stimulus competition or irrelevant information and need to resolve a conflicting representation arising from the cognitive level (Hung et al., 2018). A measure of cognitive inhibition is thus the difference in reaction time in incompatible compared to compatible or baseline trials (Sebastian et al., 2013a). Moreover, response inhibition is usually assessed by tasks such as the antisaccade task, Go/NoGo task, or stop signal task. A measure of response inhibition is the proportion of correctly withheld responses compared to incorrectly withheld actions in a NoGo or antisaccade stimulus, or reaction time in a stop signal task, which may reflect the latency of the inhibition process.

In line with the findings of behavioral studies regarding cognitive inhibition and response inhibition, studies characterizing the neural correlates of inhibitory control have also found a significant difference in neural correlates between the two components. For example, Rubia et al. (2006) demonstrated a stronger activation of the cingulo-opercular network in cognitive inhibition compared to response inhibition, whereas Sebastian et al. (2013a) revealed that cognitive inhibition activated the pre-SMA and parietal regions to a greater extent than response inhibition. More recently, through quantitatively synthesizing the published studies on inhibitory control, Hung et al. (2018) reported stronger activation of the dorsal frontal and parietal lobe in cognitive inhibition tasks compared to stronger activation of the frontostriatal network including the dorsal anterior cingulate cortex, supplementary motor cortex, lateral prefrontal cortex, basal ganglia, and parietal regions in response inhibition tasks, whereas the left anterior insula was found to be consistently activated in cognitive inhibition and response inhibition. In sum, the findings of distinct inhibitory networks in different subcomponents of inhibitory control provide supporting evidence for the differences in neural correlates between cognitive inhibition and response inhibition. Furthermore, Sebastian et al. (2013a) reported that with an increasing demand of inhibition tasks, further additional brain regions including the frontal and parietal cortices were recruited in older adults. This finding may confirm that age-related distinct brain region activation patterns in the two subcomponents of inhibitory control across the lifespan may originate from the different cognitive demand involved in the two subcomponents of inhibitory control processes; thus, this may reflect the behavioral developmental trajectory of inhibitory control.

In this study, we investigated how neural activation during the two subcomponents of inhibitory control changes across the healthy lifespan with aging using meta-analytic technology. The study had two aims: that first was to characterize the common or distinct neural correlates in the two subcomponents of inhibitory control and the second was to identify the distinct age-related activation pattern in the two subcomponents of inhibitory control. Since the cognitive resources recruited in the two subcomponents are different, we hypothesized that the activation patterns of inhibitory control may show distinct age-related trajectories in the two subcomponents of inhibitory control, or differential activation patterns may be shown across different age groups in cognitive and response inhibitions.

## Methods

### Literature search and article selection

**FIGURE 1:**
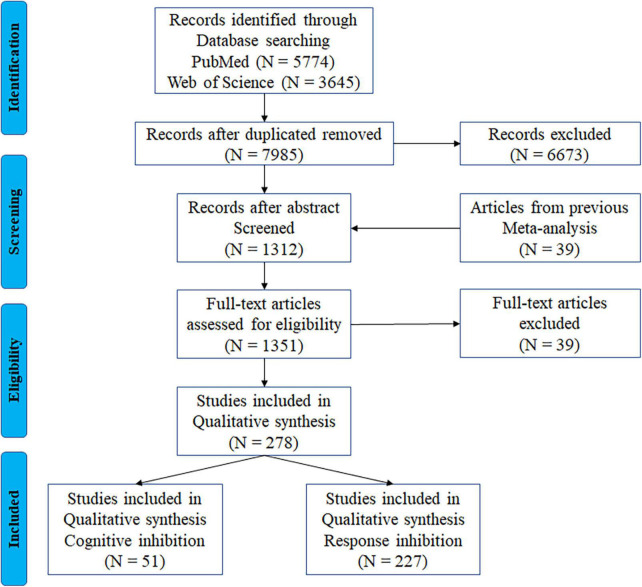
PRISMA flowchart for the identification and eligibility of articles. *N* = number of articles.

First, two online citation indexing services—PubMed and Web of Science—were searched. This search used the keywords “fMRI” with “interference resolution,” “action withholding,” “action cancellation,” “response inhibition,” “cognitive inhibition,” “inhibitory control,” “stop signal,” “stopping,” “go nogo,” “action restraint,” or “countermanding,” including articles published prior to April 2020, yielding a total of 9,419 articles. After removing duplicates, a total of 7,985 articles were screened. We then compiled 39 eligible articles identified in a previous meta-analysis (Zhang R. et al., 2017). The following exclusion criteria were applied to eliminate articles that were not directly relevant to this study: (1) non-original studies (e.g., review, abstract), (2) studies that did not report results either in Talairach or Montreal Neurology Institute (MNI) coordinate space, (3) studies with a sample size below five, (4) studies on older adults with dementia, head injury, stroke, or any neurological or other psychiatric disease, (5) pharmacological or training-related studies, unless they conducted a baseline comparison and fulfilled our inclusion criteria, (6) studies with no control group or within-group contrast, (7) studies that used positive or negative stimulation only in inhibition tasks. A total of 278 articles were included in the current meta-analysis. Figure 1 shows the detailed search and selection procedures. The final dataset was then divided into the two subcomponents of inhibitory control: 51 articles on cognitive inhibition and 227 articles on response inhibition.

### Data extraction

We extracted the following information from each study: authors, year of publication, sample size, experimental design, paradigms, mean age with the age range, task contrasts, stimulation types, and cluster coordinates in the MNI or Talairach space.

### Experiment categorization

#### Cognitive inhibition

Cognitive inhibition is the inhibitory process of suppression of competing cognitive processing to solve relevant problems (Hung et al., 2018). For the cognitive inhibition domain, we included commonly used cognitive interference paradigms (Hung et al., 2018): Stroop, Flanker, Simon task, and SRC. We examined the changes in activation between incongruent and neutral or incongruent and congruent conditions to measure straightforward processing of cognitive interference. A total of 51 articles consisting of 54 experiments were included to explore the neural correlates of cognitive inhibition. The characteristics of each study are listed in Supplementary Table 1.

#### Response inhibition

Response inhibition is the process of suppression of a prepotent response to perform a different, more context-appropriate response (Hung et al., 2018). To measure the response inhibition, we included the classical paradigms, including antisaccade, Go/NoGo, and stop signal tasks, which primarily require the inhibition of prepotent motor responses. Qualified response inhibition experimental contrasts measured the differences in activation between go and no-go or stop conditions. A total of 227 articles comprising 236 contrasts using the antisaccade, Go/NoGo paradigm, or stop signal paradigm were employed to identify the response inhibition-related activation patterns.

### Multilevel kernel density analysis

Meta-analyses were performed using the Multilevel kernel density analysis (MKDA) (Wager et al., 2007) toolbox^1^ to identify the brain regions activated during inhibitory control. Peak effect coordinates from each study were convolved with a spherical kernel (*r* = 16 mm) (Wager et al., 2004) to generate comparison indicator maps (CIMs), with a value of 1 indicating that “this study activated near this voxel” and a value of 0 indicating that “this study did not activate near this voxel.” The CIMs were averaged to yield the proportion of studies in which the activation was observed within 16 mm of each voxel. The family-wise error (FWE) rate was estimated to correct for multiple comparisons (5,000 permutations).

Previously used meta-analyses, such as the activation likelihood estimate (ALE), count how many peak coordinates are within each voxel divided by the brain regions and compare this to the number expected by chance if the peak coordinates were randomly distributed in the brain. This method is limited by the consequence that the peak coordinates in any single study may overly influence the results from analyses (Radua and Mataix-Cols, 2012). Using MKDA may overcome this limitation by separating the peaks of each study. In the MKDA method, the null hypothesis is that the n peak coordinates reported in the set of studies to be analyzed are randomly and uniformly distributed throughout the gray matter. Thus, in this study, the meta-analytic results represent common activated regions across studies: regions in which the significant activation was observed in the local neighborhood by more studies than would be expected by chance (*p* < 0.05, FWE-corrected across the entire brain). Specifically, to characterize brain activation patterns, first, we identified brain regions that showed significant convergence across 278 studies comprising 4,393 foci from 290 contrasts. Then, contrast analyses were conducted to verify the differences in cognitive demand for the two subcomponents of inhibitory control and capture the selectively or preferentially activated brain regions for two subcomponents of inhibitory control: cognitive inhibition vs. response inhibition. All cluster coordinates were analyzed in Montreal Neurological Institute (MNI) standard stereotaxic spaces.

### Effects of age on subcomponents of inhibitory control

#### Meta-regression analyses with age

To further assess age-related change in activation patterns in the subcomponents of inhibitory control, the effect-size seed-based d mapping (ES-SDM) toolbox (SdmPsiGui-v6.21 from the Seed-based d Mapping project) was used to perform meta-regression analyses because the ES-SDM software can provide accurate results of regression analyses incorporating meta-regression methods. This is achieved by first using peak coordinates and their statistical values to recreate statistical parametric maps and then conducting an image-based meta-analysis (Radua and Mataix-Cols, 2012). The full width at half maximum (FWHM) in SDM was set at 20 mm (Radua and Mataix-Cols, 2012) by default to control for false positives and the resulting statistical maps were thresholded at *p* < 0.05 to control for family-wise error rate. We performed two meta-regression analyses in ES-SDM. Data involved in the meta-regression analyses were derived from response inhibition contrasts and cognitive inhibition contrasts separately. Given that different age ranges were reported in the original articles, the age computed in the meta-regression analysis as a continuous variable was determined by the mean age of each sample in the original articles. The results from the two regression analyses were then compared to identify brain regions with distinct age-related activation patterns in the two subcomponents of inhibitory control.

#### Meta-analyses across different age groups

Further, given that age was computed in the meta-regression analysis as a continuous variable determined by the mean age of each sample in the original articles, the mean age was affected by extreme values, which cannot well represent the age distribution of all subjects in each original study. As mentioned above, to more completely explore the age-related changes in the two subcomponents of inhibitory control within individuals of different ages, we performed additional MKDA analyses as validation analyses. We divided the datasets from all articles included in the current meta-analysis into four age groups: under 18 years for under-aged children, 18–35 years for young adults, 35–55 years for middle-aged adults, and 55–80 years of age for older adults. Then, we performed contrast analyses and computed the differences among all age groups in the two subcomponents of inhibitory control: under-aged children vs. young adults, young adults vs. middle-aged adults, middle-aged adults vs. older adults, under-aged children vs. middle-aged adults, young adults vs. older adults, and under-aged children vs. older adults.

### Validation analyses

Additional validation analyses were performed to reduce the impact of the number of experiments, the different types of inhibition tasks, and the behavioral performance reported in the studies included in the results of the current meta-analysis.

First, studies on response inhibition (236 experiments of Go/NoGo and stop signal tasks) included a much larger number of experiments compared to those on cognitive inhibition (54 experiments). To test the effect of the number of experiments, we randomly selected 54 contrasts from the response inhibition data and repeated the MKDA and meta-regression analysis with age as a variable using the same settings.

Second, leave-one-out analysis was performed to test homogeneity in the cognitive inhibition tasks and response inhibition tasks separately. For the two subcomponents of inhibitory control, we removed each type of inhibition task one at a time: including Flanker, Simon, Stroop, WCST, and other tasks for cognitive inhibition, and tasks including antisaccade, Go/NoGo, and stop signal tasks for response inhibition. MKDA analyses were separately performed on the remaining studies with a total of five activation maps for cognitive inhibition and three activation maps for response inhibition. Then, we pooled the activation maps to obtain the overlapping rate maps for cognitive and response inhibitions, respectively. The overlap rate maps obtained from leave-one-out analysis and the results from MKDA on cognitive or response inhibition were then contrasted to test whether different tasks employed in studies influenced the activation patterns in the subcomponents of inhibitory control. Furthermore, since the meta-analysis results of cognitive inhibition and response inhibition identified different major regions (i.e., insula, inferior frontal gyrus, and angular gyrus), we conducted contrast analyses on the regions identified in the leave-one-out meta-analysis results for cognitive inhibition and response inhibitions separately.

Third, to investigate whether older adults that performed a task similarly to younger adults may show different patterns of activity to older adults who underperformed relative to younger adults, we selected a total of 81 studies which reported task performance with successful inhibition in response inhibition tasks. Then, we performed a meta-regression analysis and several contrast analyses separately.

## Results

### Meta-analysis of all included inhibitory control experiments

**FIGURE 2:**
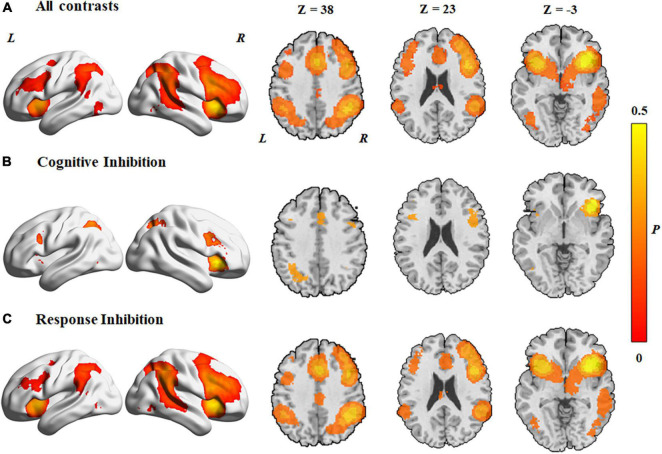
Concordance of brain activation from the MKDA analyses. **(A)** Brain areas activated by all contrasts. Brain areas activated in **(B)** cognitive inhibition and **(C)** response inhibition. The color bar represents the proportion of studies exhibiting the effect at the peak density weighted by sample size (P).

The MKDA of the 278 studies showed significant activation of clusters in both hemispheres, including the frontal cortex, the angular gyrus, and the supplementary motor area (Figure 2A). The results are provided in Supplementary Table 2.

### Brain activation patterns of each component

#### Brain activation patterns of cognitive inhibition

In both hemispheres, activated areas during the cognitive inhibition tasks included the inferior frontal gyrus, precentral gyrus, anterior insula, inferior parietal lobule, supplementary motor cortex, superior parietal lobule, superior frontal gyrus, middle cingulate gyrus, inferior frontal gyrus, and angular gyrus (Figure 2B and Table 1). Unilateral activations were observed in the right middle frontal gyrus.

**TABLE 1:** Brain activation in two subcomponents of inhibitory control.

Regions	R/L	MNI			No.Voxs	Maximum P
		x	y	z		
***Cognitive inhibition***						
Angular Gyrus	R	26	–64	48	247	0.35
Inferior Frontal Gyrus	R	44	12	24	769	0.49
Inferior Frontal Gyrus	L	–40	14	20	281	0.37
Inferior Parietal Lobule	L	–30	–58	44	870	0.4
Inferior Parietal Lobule	R	34	–52	48	131	0.34
Insula	R	38	22	–2	1696	0.57
Supplementary Motor Area	L	–2	14	48	865	0.4
***Response inhibition***						
Angular Gyrus	R	30	–60	46	1627	0.38
Inferior Frontal Gyrus	L	–38	30	4	724	0.39
Inferior Frontal Gyrus	L	–42	18	22	1239	0.4
Inferior Frontal Gyrus	R	46	14	30	2630	0.45
Inferior Parietal Lobule	R	48	–44	40	2505	0.44
Inferior Parietal Lobule	L	–50	–44	42	1540	0.31
Inferior Parietal Lobule	L	–30	–58	48	1096	0.31
Inferior Temporal Gyrus	R	42	–70	–6	362	0.21
Insula	R	40	20	–2	4106	0.53
Middle Cingulate Cortex	R	4	30	34	1550	0.38
Middle Cingulate Cortex	R	2	–24	34	348	0.21
Middle Frontal Gyrus	R	36	42	24	2229	0.37
Middle Temporal Gyrus	R	56	–28	–2	653	0.27
Occipital Gyrus	L	–38	–66	–8	396	0.2
Pallidum	R	18	4	4	1664	0.42
Precentral Gyrus	R	34	2	50	1573	0.35
Precentral Gyrus	L	–34	–2	48	906	0.23
Precuneus	R	14	–68	44	357	0.27
Superior Temporal Gyrus	L	–8	2	6	1837	0.32
Superior Temporal Gyrus	R	56	–42	14	2109	0.38
Superior Temporal Gyrus	L	–58	–46	22	576	0.25
Supplementary Motor Area	R	4	10	52	4191	0.45
Thalamus	R	4	–18	14	336	0.22

Maximum P is the maximum proportion of studies exhibiting the effect at the peak density weighted by sample size. The coordinates are Montreal Neurological Institute (MNI) standard stereotaxic spaces. The voxel size is 2 mm × 2 mm × 2 mm. R/L, right/left hemisphere.

#### Brain activation patterns of response inhibition

Data from response inhibition experiments revealed activations in the right middle frontal gyrus, the right angular gyrus that extended to the middle temporal gyrus and superior temporal gyrus, the right inferior temporal gyrus, and the left middle cingulate gyrus. In addition, activation areas in both hemispheres were observed in the supplementary motor cortex, middle cingulate gyrus, superior frontal gyrus, precentral gyrus, inferior frontal gyrus, anterior insula, inferior parietal lobule, and supramarginal gyrus (Figure 2C and Table 1).

### Common and distinct activation patterns in the two subcomponents

**FIGURE 3:**
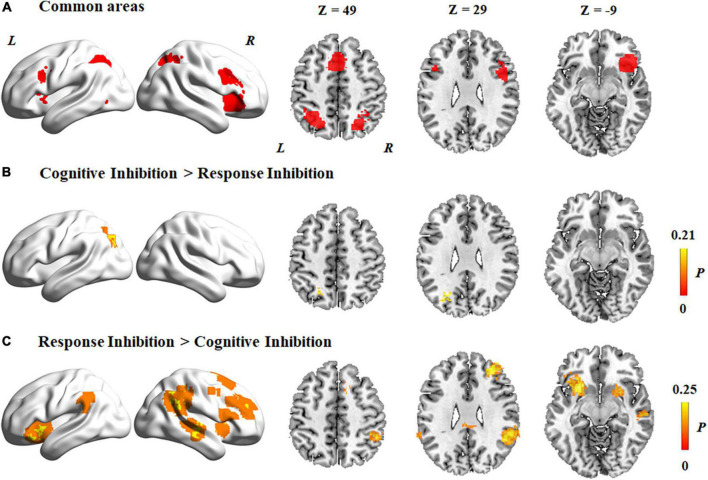
Common and distinct activation regions between two subcomponents. **(A)** Common areas between cognitive inhibition and response inhibition. **(B)** Higher activation in cognitive inhibition than response inhibition. **(C)** Higher activation in response inhibition than cognitive inhibition. The color bar represents the maximum proportion of studies exhibiting the effect at the peak density weighted by sample size (P).

Activation patterns common to the two subcomponents of inhibitory control were derived by conjunction analysis (Figure 3A). Regions commonly activated in the two subcomponents of inhibitory control included the following: (1) the supplementary motor cortex, which extended to the middle cingulate cortex and the superior parietal lobule in both hemispheres; (2) the inferior frontal gyrus, which extended to the middle frontal gyrus and insula in both hemispheres; (3) the right superior occipital gyrus and left middle occipital gyrus; and (4) the inferior parietal lobule and angular gyrus in both hemispheres.

Contrast analyses of cognitive inhibition and response inhibition revealed that significantly different regions were activated in the two subcomponents of inhibitory control. Specifically, compared to response inhibition, a higher level of activation was found during cognitive inhibition in the left superior parietal lobule (Figure 3B and Table 2). On the other hand, a higher level of activation during response inhibition compared to cognitive inhibition occurred in the frontal cortex including the bilateral insula and inferior frontal gyrus, the right middle frontal gyrus, and the right superior frontal gyrus, which extended to the bilateral putamen (Figure 3C and Table 2). Regions in the right middle temporal gyrus and right angular gyrus also showed a higher level of activation during response inhibition than in cognitive inhibition.

**TABLE 2:** Brain activation differences between cognitive inhibition and response inhibition.

Regions	R/L	MNI			No.Voxs	Maximum P
		x	y	z		
***Cognitive inhibition* > *Response inhibition***						
Occipital Gyrus	L	–28	–68	32	175	0.18
Superior Parietal Lobule	L	–22	–62	42	40	0.15
***Response inhibition* > *Cognitive inhibition***						
Inferior Frontal Gyrus	L	–36	22	–12	452	0.21
Inferior Frontal Gyrus	R	48	14	16	29	0.2
Inferior Frontal Gyrus	R	38	24	32	15	0.16
Inferior Parietal Lobule	L	–54	–48	38	10	0.15
Insula	R	30	16	10	312	0.23
Insula	L	–30	18	–8	100	0.23
Middle Cingulate Cortex	R	0	–32	30	93	0.16
Middle Frontal Gyrus	R	32	42	28	430	0.22
Middle Temporal Gyrus	R	50	–30	–2	38	0.17
Putamen	L	–24	12	0	874	0.26
Putamen	R	22	8	–2	436	0.22
Superior Temporal Gyrus	R	56	–22	–4	263	0.18
Supplementary Motor Area	R	12	14	56	291	0.25
Supplementary Motor Area	R	8	–2	60	131	0.2
Supramarginal Gyrus	R	52	–44	34	1295	0.23
Supramarginal Gyrus	L	–64	–44	30	48	0.16

Maximum P is the maximum proportion of studies exhibiting the effect at the peak density weighted by sample size. The coordinates are Montreal Neurological Institute (MNI) standard stereotaxic spaces. The voxel size is 2 mm × 2 mm × 2 mm. R/L, right/left hemisphere.

### Age-related brain activation patterns of each component

#### Age-related change in the activation patterns in cognitive inhibition

**FIGURE 4:**
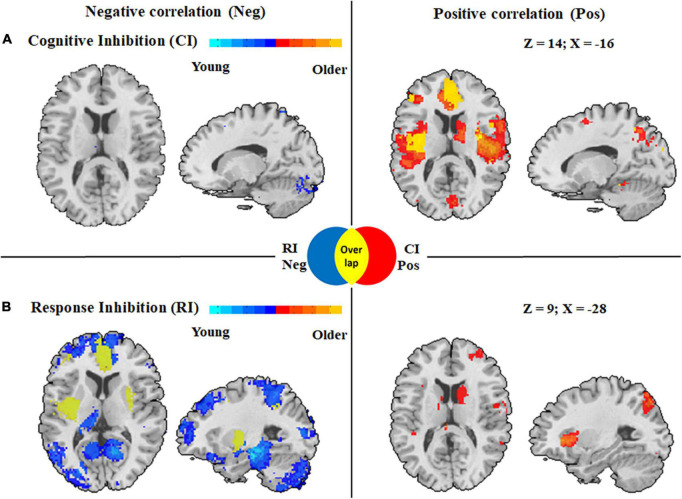
Activation maps displaying whole brain regression analysis in cognitive inhibition and response inhibition with age as a covariate. **(A)** Correlation with age with clusters in cognitive inhibition. **(B)** Correlation with age with clusters in response inhibition. Clusters associated with activity in older adults during inhibitory control are displayed in red, whereas clusters associated with activity during inhibitory control among younger adults are displayed in blue. CI Pos: Regions associated with increased activity with aging during cognitive inhibition; RI Neg: Regions associated with decreased activity with aging during response inhibition; Overlap: Regions displayed in yellow derived from overlapping results from regression analyses in two subcomponents with age, meaning a distinct correlation with age in these regions in two subcomponents of inhibitory control.

In the cognitive inhibition tasks, results from the meta-regression analysis with age as a continuous variable across all studies showed a positive association with clusters in the followings: (1) the middle cingulate cortex, anterior cingulate cortex, and insula in both hemispheres; (2) the angular gyrus, superior parietal lobule, inferior frontal gyrus, and supplementary motor cortex in the left hemisphere. In addition, a negative association between age and clusters was found in the bilateral middle frontal gyrus, left inferior parietal lobule, right angular gyrus, and right inferior frontal gyrus (Figure 4A).

#### Age-related change in the activation patterns in response inhibition

Activation in the response inhibition tasks showed significant positive correlations with age in the right angular gyrus, right middle frontal gyrus, bilateral inferior parietal lobule, and bilateral middle cingulate cortex, whereas a negative correlation with age was found in the followings: (1) the anterior cingulate cortex, inferior frontal gyrus, insula, hippocampus, and superior parietal lobule in the left hemisphere and (2) the superior frontal gyrus, cerebellum, insula, and inferior frontal gyrus in the right hemisphere (Figure 4B).

#### Distinct activation patterns with age in subcomponents of inhibitory control

To characterize distinct brain regions with age-related changes in activation patterns in the two subcomponents of inhibitory control, we overlapped the results from regression analyses in the two subcomponents with age and found different age-related activation patterns in the subcomponents (Figure 4). Activation of inhibitory regions including the left anterior cingulate cortex, left inferior frontal gyrus, bilateral insula, and left superior parietal lobule showed a positive correlation with age in the cognitive inhibition tasks, but a negative association with age in the response inhibition tasks.

### Brain activation patterns across different age groups for each component

#### Brain activation patterns across different age groups for cognitive inhibition

**FIGURE 5:**
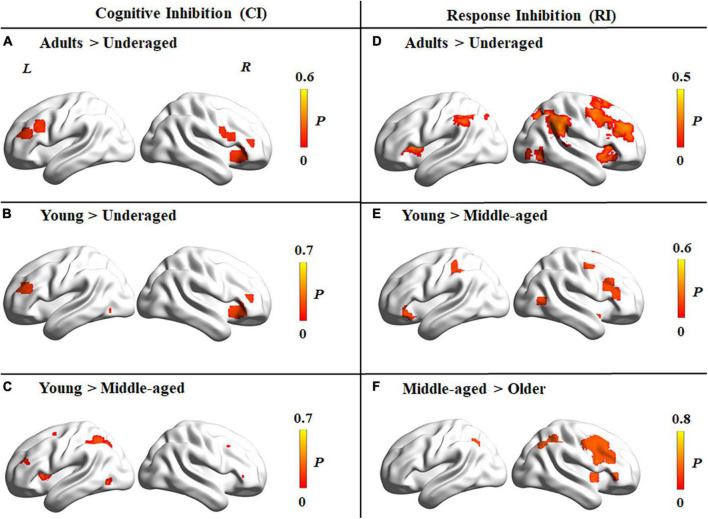
Brain activation differences among four age groups for tasks tapping cognitive inhibition and response inhibition. **(A–C)** Brain activation differences in cognitive inhibition. **(A)** Higher activation in adults than under-aged children group. **(B)** Higher activation in young than under-aged children. **(C)** Higher activation in young than middle-aged adults. **(D–F)** Brain activation differences in response inhibition. **(D)** Higher activation in adults than under-aged children group. **(E)** Higher activation in young than middle-aged adults. **(F)** Higher activation in middle-aged than older adults. The color bar represents the maximum proportion of studies exhibiting the effect at the peak density weighted by sample size (P).

Contrast analyses among different age groups for cognitive inhibition showed that regions with significantly higher levels of activation in adults (including young, middle-aged, and older adults) than under-aged children were located in the bilateral inferior frontal gyrus, right insula, and left supplementary motor area (Figure 5A). Compared to young adults, the right insula and left middle frontal gyrus were activated at a significantly lower level in under-aged children and middle-aged adults (Figures 5B,C). Further activated brain areas are reported in Supplementary Table 3.

#### Brain activation patterns across different age groups for response inhibition

Contrast analyses among different age groups for response inhibition showed that regions with significantly higher levels of activation in adults than under-aged children were located in the bilateral angular gyrus, insula, and right middle frontal gyrus (Figure 5D). Compared to middle-aged adults, the bilateral inferior frontal gyrus and left inferior parietal lobule were activated at a significantly higher level in young adults (Figure 5E), whereas a lower level of activation could be detected in the right inferior frontal gyrus, right inferior parietal lobule, and left middle cingulate cortex in older adults than middle-aged adults (Figure 5F). Further details are reported in Supplementary Table 4.

### Validation analysis

The evaluation of the number of experiments contrasting the two subcomponents showed no significant differences between the real contrasts and the randomly selected 54 experiments for response inhibition. The activated brain areas are reported in Supplementary Table 5 and Supplementary Figure 2.

The results from leave-one-out analysis (e.g., leaving out Stroop tasks from cognitive inhibition tasks) were highly consistent with the results of brain activation patterns in each component of inhibitory control derived from MKDA analyses. Further details are reported in Supplementary Tables 6, 7 and Supplementary Figure 3.

Meta-regression and contrast analyses on successful inhibition showed a similar activation pattern of brain regions including the inferior frontal gyrus, supplementary motor area, inferior parietal lobule, insula, angular gyrus, middle cingulate cortex, and occipital gyrus as compared to current MKDA analyses on response inhibition. The activated brain areas and activation maps are reported in Supplementary Tables 8 9 and Supplementary Figures 4, 5.

## Discussion

Applying MKDA and ES-SDM allowed the current meta-analysis to characterize the neural correlates and age-related effects in different subcomponents of inhibitory control. We observed brain areas including the inferior frontal gyrus, insula, middle cingulate cortex, and inferior parietal gyrus being activated in the two subcomponents. Contrast analyses conducted to elucidate the distinct neural substrates for each subcomponent revealed that relative to response inhibition, cognitive inhibition produced stronger activation in the left superior parietal lobule, whereas response inhibition primarily recruited the right inferior frontal gyrus, insula, middle temporal gyrus, and angular gyrus. Importantly, by performing a meta-regression analysis with age as a continuous variable, we found distinct age-related activation patterns in different subcomponents of inhibitory control in brain regions including the left anterior cingulate cortex, left inferior frontal gyrus, left superior parietal lobule, and bilateral insula. Overall, our results indicated common and distinct neural correlates and distinct age-related activation patterns in the two subcomponents of inhibitory control.

### Common neural activation in the two subcomponents of inhibitory control

The MKDA results showed that brain regions including the inferior frontal gyrus, insula, middle cingulate cortex, and superior parietal lobule were activated by both inhibition subcomponents. Therefore, this suggests that the inferior frontal gyrus, insula, middle cingulate cortex, and inferior parietal lobule played the core roles in inhibitory control (Yeo et al., 2011; Choi et al., 2012), which is in line with the findings of previous studies (Cieslik et al., 2015; Lemire-Rodger et al., 2019; Zhang and Iwaki, 2019). Moreover, Hobeika et al. (2016) reported the activation of domain-oriented regions within the inferior frontal gyrus and conflict-detecting regions within the middle cingulate cortex in both inhibition subcomponents, which can be interpreted as showing that either the cognitive inhibition process or response inhibition process involves the process of spatial orientation and conflict detection (Hung et al., 2018).

The main clusters of activation in the two subcomponents of inhibitory control were observed in the followings: (1) the inferior frontal gyrus extending to the insula and (2) the middle cingulate cortex and the superior parietal lobule. The inferior frontal gyrus is known to engage in the process of inhibiting automatic but irrelevant actions while activating task-relevant responses at the same time (Sharp et al., 2010; Wang et al., 2019). Moreover, the activation of the inferior frontal gyrus during detecting changes in stimulus features has also been observed (Dodds et al., 2011). Although the key role of the inferior frontal gyrus in processes of inhibitory control has been reported in a large number of previous studies (Aron et al., 2003; Swick et al., 2011), whether the right and left inferior frontal gyrus play different roles in this process is still subjected to debate. For example, Aron et al. (2003) found that patients with lesions in the right inferior frontal gyrus showed longer stop signal reaction times in stop signal tasks compared with the control group, which might strongly support inhibitory control being executed solely by the right inferior frontal gyrus. Conversely, Swick et al. (2011) found that patients with damage to the left inferior frontal gyrus and insula made more errors than did controls in a Go/NoGo task, which demonstrated that the left inferior frontal gyrus is critical for suppressing prepotent responses. In contrast, this study found that the bilateral inferior frontal gyri were activated during the process of the two subcomponents of inhibitory control. A potential explanation for this is that this study is a meta-analysis study which combined studies using different tasks and stimulus materials. The literature on frontal gyrus function shows that the left inferior frontal gyrus plays a critical role in semantic selection (Thompson-Schill et al., 1998), resolution of interference in semantic memory (Thompson-Schill et al., 2002), and conflicts from representations between incompatible word materials (Milham et al., 2001) during the processes of inhibitory control. Thus, activation of the left inferior frontal gyrus reported by Swick et al. (2011) may partly be due to the letter stimuli used in the Go/NoGo task. In line with this speculatory explanation, the literature included in the current study contained a sufficient number of different paradigms (such as Stroop, Simon, GNG, and SST) and experimental materials (such as picture, arrow, word, letter, sound; see Supplementary Table 1), which might partially explain the common activation of the bilateral inferior frontal gyrus during the two subcomponents of inhibitory control reported in the current research.

The anterior insula has been considered as the center that controls brain activity across different tasks and stimulus modalities and regulates inhibitory control mechanisms (W. Cai et al., 2019). Wager et al. (2005) reported a positive correlation between neural activity in the anterior insula and task performance in different inhibitory control tasks. One explanation for this positive correlation is that regions including the anterior insula implement a regulating process that increases with greater input conflict (Miller and Cohen, 2001). Regarding the middle cingulate cortex, studies have revealed that the middle cingulate cortex is the key region for conflict detection in information processing, reallocation of attention resources, and the formation of corresponding actions (Badzakova-Trajkov et al., 2009). When participants were required to perform a dual task, such as the Stroop task, stronger middle cingulate cortex activation could be observed (Hoffstaedter et al., 2013, 2014; Palomero-Gallagher et al., 2019). Based on the previous findings and the results on the common regions engaged in different inhibitory control tasks, we propose that the inferior frontal gyrus, insula, superior parietal lobule, and middle cingulate cortex may comprise the core neural network of the inhibitory control system.

Additionally, our research found that the right superior occipital gyrus and left middle occipital gyrus were commonly activated in the two subcomponents of inhibitory control, which is in line with the results from previous studies and meta-analyses (Simmonds et al., 2008; Sebastian et al., 2016). Interestingly, Ramautar et al. (2006) indicated that the effect of hyperactivation in the occipital cortex was only present in successful but not in unsuccessful stop trials, which is consistent with the results from validation analyses on successful inhibition contrasts in the current meta-analysis. We speculate that common activation in occipital areas across different types of inhibitory control tasks presumably reflects enhanced visual attention to the inhibition stimuli or conflict interference, considering that the inhibitory control task stimulation used in most of the studies included in our meta-analyses was presented in visual form (refer to Supplementary Table 1). Meanwhile, this enhanced activation in the occipital areas may be functionally related to successful inhibitory control, which may be explained by the fact that enhanced visual attention may facilitate the detection of inhibitory signals and thereby contribute to successful inhibitory control.

### Distinct neural activation in the two subcomponents of inhibitory control

In this meta-analysis, the inhibitory control paradigms classified as cognitive inhibition required conflict resolution and inhibition of response tendencies for successful responding (Nee et al., 2007). When performing cognitive inhibition tasks (i.e., Stroop, Simon, and Flanker tasks), participants need to actively reorient attention away from a task-relevant stimulus location or feature and then select and initiate an adequate response. Reorienting of attention mainly involves the presupplementary motor area and the superior parietal lobule. The superior parietal lobule plays an essential role in facilitating attention reallocation to characteristics of stimuli and then re-directing attention. Therefore, the significantly stronger activation in the left superior parietal lobule observed in cognitive inhibition compared to response inhibition in the current contrast analysis indicates a higher level of attention reallocation or requirement when performing cognitive inhibition tasks. This thus supports cognitive inhibition depending largely on the inhibition processes of a predominant mental set regulated by goals and conflicts (Nee et al., 2007).

In contrast, for response inhibition, the Go/NoGo and stop signal tasks encompass future action selection and inhibition of a predominant response tendency or an ongoing response, respectively. As mentioned above, the inferior frontal gyrus plays an inhibitory role in resolving conflicts during response execution, and the anterior insula is involved in the regulating process of response inhibition. Thus, there were more activated regions in response inhibition than cognitive inhibition, primarily located in the inferior frontal gyrus and anterior insula. The distinctiveness of response and cognitive inhibition, we suggest, may partly be due to the different cognitive demands in these inhibitory control tasks. Participants are required to resolve conflicts and to involve more sensory or stimulus-related neural activity in cognitive inhibition tasks, whereas the demand for inhibitory control may further increase in response inhibition tasks, which require inhibiting a predominant tendency or stopping of already-initiated actions. Furthermore, these tasks differ in terms of task-related complexity. Suppressing a response tendency or canceling an ongoing action might increase the inhibitory demand as compared to suppressing interference due to irrelevant information or resolving conflicts, as is the case in cognitive inhibition tasks (Sebastian et al., 2013a). As the engagement of the inferior frontal gyrus, middle frontal gyrus and insula play the core roles in the process of inhibitory control, activation in these regions was observed to increase with the increasing demands of inhibitory control tasks in response inhibition.

Interestingly, results from contrast analyses of cognitive inhibition and response inhibition showed a predominantly lateralized differential activation between response inhibition and cognitive inhibition. We hypothesized that the underlying reason may be the different types of stimuli (e.g., letter, word, arrow, and picture) materials used in inhibitory control tasks. By compiling a list of all types of stimulation used in the included literature (refer to Supplementary Table 1), we found that more letter-related or text-related stimuli were used in cognitive inhibition tasks, especially the Stroop task, as compared to response inhibition tasks, whereas stimuli including arrows, pictures, or dots were more frequently used in response inhibition tasks. This less verbal nature of the stimuli used in response inhibition tasks compared to cognitive inhibition tasks may ultimately reflect the right-lateralized hyperactivation involved in response inhibition, which is not present in cognitive inhibition.

### Age-related changes in activation in subcomponents of inhibitory control

In this meta-analysis, we observed neither a completely coherent increase nor a decrease in the inhibitory network with increasing age in the two subcomponents. In the cognitive inhibition tasks, activation showed a positive association with age in the anterior cingulate cortex, insula, superior parietal lobule, and inferior frontal gyrus. The results from contrast analyses among different age groups also showed that compared to under-aged children, activation in the inferior frontal gyrus and insula was stronger in adult groups. These age-related changes fit with the existing literature showing that prefrontal regions, including the inferior frontal gyrus and the middle frontal gyrus, become more active with aging (Sebastian et al., 2013b). A previous investigation of neural recruitment involved in cognitive inhibition in children aged 5–10 years reported a higher level of activation in incongruent compared with congruent trials in a network of brain regions supporting cognitive inhibition, including the frontal gyrus and parietal cortex, whereas the activation in the anterior cingulate cortex for incongruent relative to congruent trials decreased with aging (Sheridan et al., 2014). Sheridan et al. (2014) argued that as anterior cingulate cortex plays a specific role in the neural development of cognitive conflict detection and resolution, the decreased activation in the anterior cingulate cortex and general improvement in Simon task performance with aging may suggest that neural recruitment during the process of cognitive inhibition becomes more efficient with aging, which may be related to inhibitory control-related cortical thinning. Moreover, increased activation of task-related regions in the frontal and parietal cortices may reflect the cognitive function underlying cognitive inhibition tasks in younger children developing early but not being fully developed, which means that younger children need to recruit more inhibition-relevant brain regions in the frontal and parietal cortices to improve their performance in cognitive inhibition tasks. Similarly, older adults increasingly recruit additional prefrontal regions to compensate for age-related decline in brain structure and function in cognitive inhibition tasks (Sebastian et al., 2013a). Meanwhile, Nielson et al. (2002) revealed compensational activation in the left prefrontal cortex during cognitive inhibition. These results may support our assumption that a simple cognitive inhibition task requires sufficient functional compensation in the prefrontal regions recruited with aging.

A different pattern of functional age-related changes was found in the response inhibition tasks. We found the activation of the response inhibitory network, including the left anterior cingulate cortex, bilateral inferior frontal gyrus, insula, left superior parietal lobule, and right superior frontal gyrus, was negatively correlated with age. Contrast analyses also showed a linear decline with increasing age in the activation of brain regions including the bilateral inferior frontal gyrus, insula, middle cingulate cortex, and inferior parietal lobule when performing response inhibition tasks. These seemingly differential results might also be explained by the differences in the inhibition processes involved.

In line with a previous study (Reuter-Lorenz and Cappell, 2008), the current findings show that the aging brain fails to recruit additional inhibitory regions with increasing inhibitory demand and a resource ceiling is reached. With increasing task demand, the immaturity of the frontoparietal regions in younger children is associated with an inability to recruit additional inhibition-related brain regions during response inhibition tasks to maintain task performance (Bunge et al., 2002), whereas relative hypoactivation in both the core and expanded inhibitory networks in older adults may further represent a limitation of abilities for flexibly recruiting additional inhibitory networks (Cappell et al., 2010; Schneider-Garces et al., 2010). Prakash et al. (2009) pointed out that the flexibility of the cortical regions becomes limited in older adults with the number of conflicts increasing. It is important to note that the above-mentioned theories have partly been based on the studies investigating age-related differences in working memory. Turner and Spreng (2012) reported differential changes in the activation patterns for the working memory with different cognitive loads and inhibitions with age. Similarly, Sebastian et al. (2013a) showed different activation patterns in the prefrontal regions during inhibition with medium and low inhibition resource demand. Our results and those of these studies indicate that inhibition tasks with high demand might result in limited allocation of cognitive resources in older adults, which can be reflected in declined performance associated with a lower activation of inhibitory networks (Bloemendaal et al., 2018; Billig et al., 2020).

### Implications and future outlook

Several neuroimaging studies have contributed greatly to enhancing our knowledge of the neural correlates of subcomponents of inhibitory control or age-related change in the activation in the two subcomponents (Wright et al., 2014; Hung et al., 2018; Zhang R. et al., 2017). However, these studies are limited because of their focus on a restricted age range (Nielson et al., 2002, 2004), their inclusion of a single subcomponent of inhibitory control (Simmonds et al., 2008; Wright et al., 2014; Hu et al., 2018), or their use of a small sample size (Tsvetanov et al., 2018). For example, Simmonds et al. (2008) included only 11 studies in their meta-analysis. It has been argued that to ensure the replicability of a meta-analysis, it should include at least 20 studies (Eickhoff et al., 2016), as otherwise, the conclusions may be questionable. Moreover, Tsvetanov et al. (2018) conducted a study on activity and connectivity differences underlying inhibitory control across the adult lifespan using only response inhibition tasks, including Go/NoGo and stop signal tasks. Hung et al. (2018) reported that unique neural activity was associated with different inhibitory control tasks, but the age-related effects on different types of inhibitory control tasks were unknown. Our meta-analysis addressed these limitations and provided an updated review; thus, our understanding on changes in the neural correlates underlying inhibitory control with aging has been further advanced.

Through synthesizing data from different subcomponents, we found that brain regions including the inferior frontal gyrus and anterior insula, as well as regions including the middle cingulate cortex and supplementary motor cortex, were consistently activated across all inhibition tasks. This finding may suggest that these brain areas are core inhibitory control regions. Meanwhile, different age-related changes in the activation between the subcomponents of inhibitory control could be observed. Functional reorganization of the aging brain in different inhibitory control tasks showed a complex pattern of increase and decline: the corresponding cognitive inhibition tasks required older adults to increasingly recruit the core inhibitory network and additional inhibitory regions, such as the frontal regions and bilateral insula. However, a contrary pattern of age-related decline in the inhibitory network including prefrontal areas and middle cingulate cortex was shown during the process of response inhibition. The current results suggest that these differences might result from the increasing demands on inhibitory function from cognitive inhibition to response inhibition. Furthermore, age-related increased activation of additional inhibitory networks was limited. When the task demand exceeded the older adults’ capacity, activation in the inhibitory network decreased clearly. These findings are of significance for the understanding of the neuro-developmental mechanisms of inhibitory control and may provide insights into inhibitory control deficits in clinical settings.

As mentioned above, common and distinct activation patterns are involved in the processes of proactive and reactive inhibition, and age-related changes in activation patterns in proactive and reactive inhibition have been investigated. Experimental evidence from neuroimaging studies supports a right-lateralized frontoparietal circuit being widely recruited through the reactive inhibition process (Yanaka et al., 2010; Gavazzi et al., 2019). In addition, studies currently available on brain substrates of the proactive inhibition process suggest that proactive inhibition seems to rely on a wide network including the presupplementary motor area, right inferior frontal gyrus, and inferior parietal cortex (Aron et al., 2014; Cai et al., 2016). Moreover, Bloemendaal et al. (2016) explored whether age-related neurocognitive deficits in inhibitory control reflect impairments in the proactive inhibition process or reactive inhibition process; they reported that relative to young adults, older adults exhibited impaired reactive inhibition and proactive slowing in the left frontal cortex and cerebellum. Similarly, Kleerekooper et al. (2016) found that with advancing age, the patterns of activation in the right inferior frontal gyrus and motor cortex showed a clear age effect on both proactive and reactive inhibitions. The data collected in the current meta-analysis from stop signal tasks and Go/NoGo tasks to assess response inhibition drew more on proactive than reactive inhibition for suppressing prepotent responses. Although previous studies have not reported a distinct age-related activation pattern of inhibitory control in proactive and reactive inhibitions, the division of inhibitory control in the two subcomponents of cognitive inhibition and response inhibition found in this study is a robust finding on differential age-related activation patterns. However, the heterogeneity of the response inhibition tasks should still be a point of concern for future research.

### Methodological considerations

We acknowledge that the current study still has some limitations. First, we note that a potential limitation in meta-analysis methods in general is that any meta-analysis method is prone to publication bias; in this study, we only considered the results available in the published literature and original studies which reported coordinates. Moreover, we could not control the statistical methods used in original articles for thresholding the data. There is an emerging trend to store unthresholded statistical maps, which will allow image-based meta-analyses to be performed in the future studies (Gorgolewski et al., 2015).

The second limitation relates to the bias of employing different tasks for measuring the components of inhibitory control and including studies using a variety of stimulation types. Specifically, our meta-analysis adopted a mixture of inhibitory control tasks as different subcomponents of inhibitory control, such as the Stroop, Simon, and Flanker tasks used to capture cognitive inhibition and the GNG, stop signal task, and antisaccade tasks to capture response inhibition, which consequently increased the heterogeneity of the study designs. Leave-one-out analysis was performed as validation analysis to test homogeneity in cognitive inhibition and response inhibition separately. The results from leave-one-out analysis were consistent with the results of brain activation patterns in each component of inhibitory control from the MKDA analyses and the details are reported in Supplementary Tables 4, 5 and Supplementary Figure 3. Nevertheless, future meta-analyses including additional studies with positive or negative stimulations are needed.

Third, considering that task difficulty and behavioral performance in different inhibition tasks may influence age-related brain activity in different individuals, and not all studies included in the current meta-analysis reported behavioral performance for each participant or task difficulty, we selected a total of 81 studies which reported task performance with successful inhibition in response inhibition tasks. Then, we performed a meta-regression analysis and several contrast analyses separately. The results (refer Supplementary Tables 8, 9 and Supplementary Figures 4, 5) were consistent with the current research, which may confirm the reliability and stability of the current research to a certain extent. However, further considering the study-level task difficulty or behavioral performance reported in each study included in the research is still critical for future meta-analysis.

Fourth, the findings from this study are of significance for the understanding of the neuro-developmental mechanisms of inhibitory control and may provide insights into cognitive health in older adults. However, cognitive health is affected not only by physiological factors such as age, but also by socio-demographic factors; particularly significant is educational attainment. Understanding the links between education and cognitive health or cognitive aging could improve the cognitive prognoses and offer clues about the mechanisms underlying cognitive decline that can be targeted by interventions. Previous studies found that a better level of education might affect cognitive ability in older adults and attenuate aging-related decline in cognition functions (Lövdén et al., 2020; Zahodne and Zajacova, 2020). This may suggest that educational attainment can improve individual behavioral performance in inhibitory control tasks with aging and affect age-related patterns of brain activation during different subcomponents of inhibitory control. Thus, it is critical in the future research to include the level of education as an influencing factor to explore the commonalities or specificity of behavioral performance and age-related patterns of brain activation in inhibitory control tasks between groups with different levels of educational attainment.

## Conclusion

In this meta-analysis, we examined the neural correlates of subcomponents of inhibitory control and the difference in age-related changes in activation between the subcomponents. Activation of the middle cingulate cortex, supplementary motor area, inferior frontal gyrus, inferior parietal lobule, and anterior insula was common across the different inhibition processes, which revealed that these regions are the core neural system engaged in inhibitory control. On the other hand, differences in the activation patterns of subcomponents of inhibitory control with aging showed a complex pattern in the functional reorganization of the aging brain. Specifically, when performing cognitive inhibition tasks, stronger activation of the core inhibition regions was observed in older adults, whereas activation in prefrontal areas in older adults declined during response inhibition tasks. We summarize that these differences may be driven by the different demands between inhibitory control tasks. Aging individuals recruit additional inhibition-related brain regions when performing an inhibitory control task. However, with an increasing demand of inhibition tasks, limited reallocation of cognitive resources in older adults eventually results in a lower level of the activation of inhibitory brain regions in older adults during inhibitory control processes. These results may further enhance our knowledge of age-related changes in the activation patterns of inhibitory control and may provide insights into inhibitory control deficits in clinical settings.

## Data availability statement

The original contributions presented in the study are included in the article/Supplementary material, further inquiries can be directed to the corresponding author/s.

## Ethics statement

The study was reviewed and approved by the Institutional Review Board (IRB) of Southern Medical University.

## Author contributions

JL: conceptualization, data curation, data analysis, writing the manuscript, reviewing, and editing. XS, YW, CW, and RH: writing—reviewing and editing. RZ: conceptualization, supervision, funding acquisition, and writing—reviewing and editing. All authors contributed to the article and approved the submitted version.

## Footnotes

1
https://www.colorado.edu/ics/research/wager-lab

## References

[B1] Aïte A. Cassotti M. Linzarini A. Osmont A. Houdé O. Borst G. (2018). Adolescents’ inhibitory control: Keep it cool or lose control. *Dev. Sci.* 21:e12491. 10.1111/desc.12491 27882631

[B2] Almdahl I. S. Martinussen L. J. Agartz I. Hugdahl K. Korsnes M. S. (2021). Inhibition of emotions in healthy aging: age-related differences in brain network connectivity. *Brain Behav.* 11:e02052. 10.1002/brb3.2052 33543596 PMC8119855

[B3] Andrés P. Guerrini C. Phillips L. H. Perfect T. J. (2008). Differential effects of aging on executive and automatic inhibition. *Dev. Neuropsychol.* 33 101–123. 10.1080/87565640701884212 18443972

[B4] Anguera J. A. Gazzaley A. (2012). Dissociation of motor and sensory inhibition processes in normal aging. *Clin. Neurophysiol.* 123 730–740. 10.1016/j.clinph.2011.08.024 21963321 PMC3269557

[B5] Aron A. R. (2007). The neural basis of inhibition in cognitive control. *Neuroscientist* 13 214–228. 10.1177/1073858407299288 17519365

[B6] Aron A. R. (2011). From reactive to proactive and selective control: developing a richer model for stopping inappropriate responses. *Biol. Psychiatry* 69:e55–e68. 10.1016/j.biopsych.2010.07.024 20932513 PMC3039712

[B7] Aron A. R. Fletcher P. C. Bullmore E. T. Sahakian B. J. Robbins T. W. (2003). Stop-signal inhibition disrupted by damage to right inferior frontal gyrus in humans. *Nat. Neurosci.* 6 115–116. 10.1038/nn1003 12536210

[B8] Aron A. R. Robbins T. W. Poldrack R. A. (2014). Inhibition and the right inferior frontal cortex: one decade on. *Trends Cogn. Sci.* 18 177–185. 10.1016/j.tics.2013.12.003 24440116

[B9] Badzakova-Trajkov G. Barnett K. J. Waldie K. E. Kirk I. J. (2009). An ERP investigation of the Stroop task: the role of the cingulate in attentional allocation and conflict resolution. *Brain Res.* 1253 139–148. 10.1016/j.brainres.2008.11.069 19084509

[B10] Bartholdy S. O’Daly O. G. Campbell I. C. Banaschewski T. Barker G. Bokde A. L. W. (2019). Neural Correlates of Failed Inhibitory Control as an Early Marker of Disordered Eating in Adolescents. *Biol. Psychiatry* 85 956–965. 10.1016/j.biopsych.2019.01.027 31122340

[B11] Billig A. R. Feng N. C. Behforuzi H. McFeeley B. M. Nicastri C. M. Daffner K. R. (2020). Capacity-limited resources are used for managing sensory degradation and cognitive demands: implications for age-related cognitive decline and dementia. *Cortex* 133 277–294. 10.1016/j.cortex.2020.09.005 33157347

[B12] Bloemendaal M. Froböse M. I. Wegman J. Zandbelt B. B. van de Rest O. Cools R. (2018). Neuro-Cognitive Effects of Acute Tyrosine Administration on Reactive and Proactive Response Inhibition in Healthy Older Adults. *eNeuro* 5:ENEURO.0035-17.2018. 10.1523/eneuro.0035-17.2018 30094335 PMC6084775

[B13] Bloemendaal M. Zandbelt B. Wegman J. van de Rest O. Cools R. Aarts E. (2016). Contrasting neural effects of aging on proactive and reactive response inhibition. *Neurobiol. Aging* 46 96–106. 10.1016/j.neurobiolaging.2016.06.007 27460154

[B14] Booth J. R. Burman D. D. Meyer J. R. Lei Z. Trommer B. L. Davenport N. D. (2003). Neural development of selective attention and response inhibition. *Neuroimage* 20 737–751. 10.1016/s1053-8119(03)00404-x 14568448

[B15] Braver T. S. (2012). The variable nature of cognitive control: a dual mechanisms framework. *Trends Cogn. Sci.* 16 106–113. 10.1016/j.tics.2011.12.010 22245618 PMC3289517

[B16] Brevers D. He Q. Keller B. Noël X. Bechara A. (2017). Neural correlates of proactive and reactive motor response inhibition of gambling stimuli in frequent gamblers. *Sci. Rep.* 7:7394. 10.1038/s41598-017-07786-5 28785029 PMC5547049

[B17] Bunge S. A. Dudukovic N. M. Thomason M. E. Vaidya C. J. Gabrieli J. D. (2002). Immature frontal lobe contributions to cognitive control in children: evidence from fMRI. *Neuron* 33 301–311. 10.1016/s0896-6273(01)00583-9 11804576 PMC4535916

[B18] Cai W. Duberg K. Padmanabhan A. Rehert R. Bradley T. Carrion V. (2019). Hyperdirect insula-basal-ganglia pathway and adult-like maturity of global brain responses predict inhibitory control in children. *Nat. Commun.* 10:4798. 10.1038/s41467-019-12756-8 31641118 PMC6805945

[B19] Cai Y. Li S. Liu J. Li D. Feng Z. Wang Q. (2016). The role of the frontal and parietal cortex in proactive and reactive inhibitory control: a transcranial direct current stimulation study. *J. Cogn. Neurosci.* 28 177–186. 10.1162/jocn_a_00888 26439269

[B20] Cappell K. A. Gmeindl L. Reuter-Lorenz P. A. (2010). Age differences in prefontal recruitment during verbal working memory maintenance depend on memory load. *Cortex* 46 462–473. 10.1016/j.cortex.2009.11.009 20097332 PMC2853232

[B21] Choi E. Y. Yeo B. T. Buckner R. L. (2012). The organization of the human striatum estimated by intrinsic functional connectivity. *J. Neurophysiol.* 108 2242–2263. 10.1152/jn.00270.2012 22832566 PMC3545026

[B22] Cieslik E. C. Mueller V. I. Eickhoff C. R. Langner R. Eickhoff S. B. (2015). Three key regions for supervisory attentional control: evidence from neuroimaging meta-analyses. *Neurosci. Biobehav. Rev.* 48 22–34. 10.1016/j.neubiorev.2014.11.003 25446951 PMC4272620

[B23] Cope L. M. Hardee J. E. Martz M. E. Zucker R. A. Nichols T. E. Heitzeg M. M. (2020). Developmental maturation of inhibitory control circuitry in a high-risk sample: a longitudinal fMRI study. *Dev. Cogn. Neurosci.* 43:100781. 10.1016/j.dcn.2020.100781 32510344 PMC7212183

[B24] Dalley J. W. Everitt B. J. Robbins T. W. (2011). Impulsivity, compulsivity, and top-down cognitive control. *Neuron* 69 680–694. 10.1016/j.neuron.2011.01.020 21338879

[B25] Dodds C. M. Morein-Zamir S. Robbins T. W. (2011). Dissociating inhibition, attention, and response control in the frontoparietal network using functional magnetic resonance imaging. *Cereb. Cortex* 21 1155–1165. 10.1093/cercor/bhq187 20923963 PMC3077432

[B26] Durston S. Thomas K. M. Worden M. S. Yang Y. Casey B. J. (2002). The effect of preceding context on inhibition: an event-related fMRI study. *Neuroimage* 16 449–453. 10.1006/nimg.2002.1074 12030830

[B27] Eickhoff S. B. Dafotakis M. Grefkes C. Shah N. J. Zilles K. Piza-Katzer H. (2008). Central adaptation following heterotopic hand replantation probed by fMRI and effective connectivity analysis. *Exp. Neurol.* 212 132–144. 10.1016/j.expneurol.2008.03.025 18501895

[B28] Eickhoff S. B. Nichols T. E. Laird A. R. Hoffstaedter F. Amunts K. Fox P. T. (2016). Behavior, sensitivity, and power of activation likelihood estimation characterized by massive empirical simulation. *Neuroimage* 137 70–85. 10.1016/j.neuroimage.2016.04.072 27179606 PMC4981641

[B29] Fernandez-Ruiz J. Peltsch A. Alahyane N. Brien D. C. Coe B. C. Garcia A. (2018). Age related prefrontal compensatory mechanisms for inhibitory control in the antisaccade task. *Neuroimage* 165 92–101. 10.1016/j.neuroimage.2017.10.001 28988829

[B30] Gavazzi G. Giovannelli F. Currò T. Mascalchi M. Viggiano M. P. (2021). Contiguity of proactive and reactive inhibitory brain areas: a cognitive model based on ALE meta-analyses. *Brain Imaging Behav.* 15 2199–2214. 10.1007/s11682-020-00369-5 32748318 PMC8413163

[B31] Gavazzi G. Rossi A. Orsolini S. Diciotti S. Giovannelli F. Salvadori E. (2019). Impulsivity trait and proactive cognitive control: An fMRI study. *Eur. J. Neurosci.* 49 1171–1179. 10.1111/ejn.14301 30549328

[B32] Goldman-Rakic P. S. (1996). The prefrontal landscape: implications of functional architecture for understanding human mentation and the central executive. *Philos. Trans R. Soc. Lond. B. Biol. Sci.* 351 1445–1453. 10.1098/rstb.1996.0129 8941956

[B33] Gorgolewski K. J. Varoquaux G. Rivera G. Schwarz Y. Ghosh S. S. Maumet C. (2015). NeuroVault.org: a web-based repository for collecting and sharing unthresholded statistical maps of the human brain. *Front. Neuroinform.* 9:8. 10.3389/fninf.2015.00008 25914639 PMC4392315

[B34] Hasher L. Zacks R. T. (1988). Working Memory, Comprehension, and Aging: A Review and a New View. In Bower G. H. (Ed) *The Psychology of Learning and Motivation: Advances in Research and Theory*, Vol. 22. San Diego, CA: Academic Press, 193–225.

[B35] Hobeika L. Diard-Detoeuf C. Garcin B. Levy R. Volle E. (2016). General and specialized brain correlates for analogical reasoning: A meta-analysis of functional imaging studies. *Hum. Brain Mapp.* 37 1953–1969. 10.1002/hbm.23149 27012301 PMC6867453

[B36] Hoffstaedter F. Grefkes C. Caspers S. Roski C. Palomero-Gallagher N. Laird A. R. (2014). The role of anterior midcingulate cortex in cognitive motor control: evidence from functional connectivity analyses. *Hum. Brain Mapp.* 35 2741–2753. 10.1002/hbm.22363 24115159 PMC5293144

[B37] Hoffstaedter F. Grefkes C. Zilles K. Eickhoff S. B. (2013). The “what” and “when” of self-initiated movements. *Cereb. Cortex* 23 520–530. 10.1093/cercor/bhr391 22414772 PMC3593700

[B38] Hu S. Ide J. S. Chao H. H. Castagna B. Fischer K. A. Zhang S. (2018). Structural and functional cerebral bases of diminished inhibitory control during healthy aging. *Hum. Brain Mapp.* 39 5085–5096. 10.1002/hbm.24347 30113124 PMC6287913

[B39] Humphrey G. Dumontheil I. (2016). Development of Risk-Taking, Perspective-Taking, and Inhibitory Control During Adolescence. *Dev. Neuropsychol.* 41 59–76. 10.1080/87565641.2016.1161764 27070826

[B40] Hung Y. Gaillard S. L. Yarmak P. Arsalidou M. (2018). Dissociations of cognitive inhibition, response inhibition, and emotional interference: voxelwise ALE meta-analyses of fMRI studies. *Hum. Brain Mapp.* 39 4065–4082. 10.1002/hbm.24232 29923271 PMC6866358

[B41] Kan Y. Xue W. Zhao H. Wang X. Guo X. Duan H. (2021). The discrepant effect of acute stress on cognitive inhibition and response inhibition. *Conscious Cogn.* 91:103131. 10.1016/j.concog.2021.103131 33862365

[B42] Kleerekooper I. van Rooij S. J. H. van den Wildenberg W. P. M. de Leeuw M. Kahn R. S. Vink M. (2016). The effect of aging on fronto-striatal reactive and proactive inhibitory control. *Neuroimage* 132 51–58. 10.1016/j.neuroimage.2016.02.031 26899783

[B43] Lemire-Rodger S. Lam J. Viviano J. D. Stevens W. D. Spreng R. N. Turner G. R. (2019). Inhibit, switch, and update: a within-subject fMRI investigation of executive control. *Neuropsychologia* 132:107134. 10.1016/j.neuropsychologia.2019.107134 31299188

[B44] Lövdén M. Fratiglioni L. Glymour M. M. Lindenberger U. Tucker-Drob E. M. (2020). Education and Cognitive Functioning Across the Life Span. *Psychol. Sci. Public Interest* 21 6–41. 10.1177/1529100620920576 32772803 PMC7425377

[B45] Luna B. Garver K. E. Urban T. A. Lazar N. A. Sweeney J. A. (2004). Maturation of cognitive processes from late childhood to adulthood. *Child Dev.* 75 1357–1372. 10.1111/j.1467-8624.2004.00745.x 15369519

[B46] Meyer H. C. Bucci D. J. (2016). Neural and behavioral mechanisms of proactive and reactive inhibition. *Learn. Mem.* 23 504–514. 10.1101/lm.040501.115 27634142 PMC5026209

[B47] Milham M. P. Banich M. T. Webb A. Barad V. Cohen N. J. Wszalek T. (2001). The relative involvement of anterior cingulate and prefrontal cortex in attentional control depends on nature of conflict. *Brain Res. Cogn. Brain Res.* 12 467–473. 10.1016/s0926-6410(01)00076-3 11689307

[B48] Miller E. K. Cohen J. D. (2001). An integrative theory of prefrontal cortex function. *Annu. Rev. Neurosci.* 24 167–202. 10.1146/annurev.neuro.24.1.167 11283309

[B49] Miyake A. Friedman N. P. Emerson M. J. Witzki A. H. Howerter A. Wager T. D. (2000). The unity and diversity of executive functions and their contributions to complex “Frontal Lobe” tasks: a latent variable analysis. *Cogn. Psychol.* 41 49–100. 10.1006/cogp.1999.0734 10945922

[B50] Nee D. E. Wager T. D. Jonides J. (2007). Interference resolution: insights from a meta-analysis of neuroimaging tasks. *Cogn. Affect. Behav. Neurosci.* 7 1–17. 10.3758/cabn.7.1.1 17598730

[B51] Nielson K. A. Langenecker S. A. Garavan H. (2002). Differences in the functional neuroanatomy of inhibitory control across the adult life span. *Psychol. Aging* 17 56–71. 10.1037//0882-7974.17.1.56 11931287

[B52] Nielson K. A. Langenecker S. A. Ross T. J. Garavan H. Rao S. M. Stein E. A. (2004). Comparability of functional MRI response in young and old during inhibition. *Neuroreport* 15 129–133. 10.1097/00001756-200401190-00025 15106844 PMC2078238

[B53] Noreen S. MacLeod M. D. (2015). What Do We Really Know about Cognitive Inhibition? Task Demands and Inhibitory Effects across a Range of Memory and Behavioural Tasks. *PLoS One* 10:e0134951. 10.1371/journal.pone.0134951 26270470 PMC4536050

[B54] Ordaz S. J. Foran W. Velanova K. Luna B. (2013). Longitudinal growth curves of brain function underlying inhibitory control through adolescence. *J. Neurosci.* 33 18109–18124. 10.1523/jneurosci.1741-13.2013 24227721 PMC3828464

[B55] Palomero-Gallagher N. Hoffstaedter F. Mohlberg H. Eickhoff S. B. Amunts K. Zilles K. (2019). Human pregenual anterior cingulate cortex: structural, functional, and connectional heterogeneity. *Cereb. Cortex* 29 2552–2574. 10.1093/cercor/bhy124 29850806 PMC6519696

[B56] Paus T. Keshavan M. Giedd J. N. (2008). Why do many psychiatric disorders emerge during adolescence? *Nat. Rev. Neurosci.* 9 947–957. 10.1038/nrn2513 19002191 PMC2762785

[B57] Prakash R. S. Erickson K. I. Colcombe S. J. Kim J. S. Voss M. W. Kramer A. F. (2009). Age-related differences in the involvement of the prefrontal cortex in attentional control. *Brain Cogn.* 71 328–335. 10.1016/j.bandc.2009.07.005 19699019 PMC2783271

[B58] Radua J. Mataix-Cols D. (2012). Meta-analytic methods for neuroimaging data explained. *Biol. Mood Anxiety Disord.* 2:6. 10.1186/2045-5380-2-6 22737993 PMC3384225

[B59] Ramautar J. R. Slagter H. A. Kok A. Ridderinkhof K. R. (2006). Probability effects in the stop-signal paradigm: the insula and the significance of failed inhibition. *Brain Res.* 1105 143–154. 10.1016/j.brainres.2006.02.091 16616048

[B60] Reuter-Lorenz P. A. Cappell K. A. (2008). Neurocognitive aging and the compensation hypothesis. *Curr. Direct. Psychol. Sci.* 17 177–182. 10.1111/j.1467-8721.2008.00570.x

[B61] Rubia K. Lim L. Ecker C. Halari R. Giampietro V. Simmons A. (2013). Effects of age and gender on neural networks of motor response inhibition: from adolescence to mid-adulthood. *Neuroimage* 83 690–703. 10.1016/j.neuroimage.2013.06.078 23845427

[B62] Rubia K. Smith A. B. Woolley J. Nosarti C. Heyman I. Taylor E. (2006). Progressive increase of frontostriatal brain activation from childhood to adulthood during event-related tasks of cognitive control. *Hum. Brain Mapp.* 27 973–993. 10.1002/hbm.20237 16683265 PMC6871373

[B63] Schachar R. J. Logan G. D. J. D. P. (1990). Impulsivity and Inhibitory Control in Normal Development and Childhood Psychopathology. *Dev. Psychol.* 26 710–720. 10.1016/j.jneumeth.2005.08.023 16427129

[B64] Schneider-Garces N. J. Gordon B. A. Brumback-Peltz C. R. Shin E. Lee Y. Sutton B. P. (2010). Span, CRUNCH, and beyond: working memory capacity and the aging brain. *J. Cogn. Neurosci.* 22 655–669. 10.1162/jocn.2009.21230 19320550 PMC3666347

[B65] Sebastian A. Baldermann C. Feige B. Katzev M. Scheller E. Hellwig B. (2013a). Differential effects of age on subcomponents of response inhibition. *Neurobiol. Aging* 34 2183–2193. 10.1016/j.neurobiolaging.2013.03.013 23591131

[B66] Sebastian A. Pohl M. F. Klöppel S. Feige B. Lange T. Stahl C. (2013b). Disentangling common and specific neural subprocesses of response inhibition. *Neuroimage* 64 601–615. 10.1016/j.neuroimage.2012.09.020 22986077

[B67] Sebastian A. Jung P. Neuhoff J. Wibral M. Fox P. T. Lieb K. (2016). Dissociable attentional and inhibitory networks of dorsal and ventral areas of the right inferior frontal cortex: a combined task-specific and coordinate-based meta-analytic fMRI study. *Brain Struct. Funct.* 221 1635–1651. 10.1007/s00429-015-0994-y 25637472 PMC4791198

[B68] Sharp D. J. Bonnelle V. De Boissezon X. Beckmann C. F. James S. G. Patel M. C. (2010). Distinct frontal systems for response inhibition, attentional capture, and error processing. *Proc. Natl. Acad. Sci. U.S.A.* 107 6106–6111. 10.1073/pnas.1000175107 20220100 PMC2851908

[B69] Sheridan M. Kharitonova M. Martin R. E. Chatterjee A. Gabrieli J. D. (2014). Neural substrates of the development of cognitive control in children ages 5-10 years. *J. Cogn. Neurosci.* 26 1840–1850. 10.1162/jocn_a_00597 24650280 PMC4080218

[B70] Simmonds D. J. Pekar J. J. Mostofsky S. H. (2008). Meta-analysis of Go/No-go tasks demonstrating that fMRI activation associated with response inhibition is task-dependent. *Neuropsychologia* 46 224–232. 10.1016/j.neuropsychologia.2007.07.015 17850833 PMC2327217

[B71] Stahl C. Voss A. Schmitz F. Nuszbaum M. Tüscher O. Lieb K. (2014). Behavioral components of impulsivity. *J. Exp. Psychol. Gen.* 143 850–886. 10.1037/a0033981 23957282

[B72] Steele V. R. Maurer J. M. Arbabshirani M. R. Claus E. D. Fink B. C. Rao V. (2018). Machine Learning of Functional Magnetic Resonance Imaging Network Connectivity Predicts Substance Abuse Treatment Completion. *Biol. Psychiatry Cogn. Neurosci. Neuroimaging* 3 141–149. 10.1016/j.bpsc.2017.07.003 29529409 PMC5851466

[B73] Swick D. Ashley V. Turken U. (2011). Are the neural correlates of stopping and not going identical? Quantitative meta-analysis of two response inhibition tasks. *Neuroimage* 56 1655–1665. 10.1016/j.neuroimage.2011.02.070 21376819

[B74] Tamm L. Menon V. Reiss A. L. (2002). Maturation of brain function associated with response inhibition. *J. Am. Acad. Child Adolesc. Psychiatry* 41 1231–1238. 10.1097/00004583-200210000-00013 12364845

[B75] Thompson-Schill S. L. Jonides J. Marshuetz C. Smith E. E. D’Esposito M. Kan I. P. (2002). Effects of frontal lobe damage on interference effects in working memory. *Cogn. Affect. Behav. Neurosci.* 2 109–120. 10.3758/cabn.2.2.109 12455679

[B76] Thompson-Schill S. L. Swick D. Farah M. J. D’Esposito M. Kan I. P. Knight R. T. (1998). Verb generation in patients with focal frontal lesions: a neuropsychological test of neuroimaging findings. *Proc. Natl. Acad. Sci. U.S.A.* 95 15855–15860. 10.1073/pnas.95.26.15855 9861060 PMC28134

[B77] Tsvetanov K. A. Ye Z. Hughes L. Samu D. Treder M. S. Wolpe N. (2018). Activity and Connectivity Differences Underlying Inhibitory Control Across the Adult Life Span. *J. Neurosci.* 38 7887–7900. 10.1523/jneurosci.2919-17.2018 30049889 PMC6125816

[B78] Turner G. R. Spreng R. N. (2012). Executive functions and neurocognitive aging: dissociable patterns of brain activity. *Neurobiol. Aging* 33:826.e1-13. 10.1016/j.neurobiolaging.2011.06.005 21791362

[B79] van Belle J. Vink M. Durston S. Zandbelt B. B. (2014). Common and unique neural networks for proactive and reactive response inhibition revealed by independent component analysis of functional MRI data. *Neuroimage* 103 65–74. 10.1016/j.neuroimage.2014.09.014 25224995

[B80] van Velzen L. S. Vriend C. de Wit S. J. van den Heuvel O. A. (2014). Response inhibition and interference control in obsessive-compulsive spectrum disorders. *Front. Hum. Neurosci.* 8:419. 10.3389/fnhum.2014.00419 24966828 PMC4052433

[B81] Velanova K. Wheeler M. E. Luna B. (2008). Maturational changes in anterior cingulate and frontoparietal recruitment support the development of error processing and inhibitory control. *Cereb. Cortex* 18 2505–2522. 10.1093/cercor/bhn012 18281300 PMC2733315

[B82] Velanova K. Wheeler M. E. Luna B. (2009). The maturation of task set-related activation supports late developmental improvements in inhibitory control. *J. Neurosci.* 29 12558–12567. 10.1523/jneurosci.1579-09.2009 19812330 PMC2788337

[B83] Vink M. Zandbelt B. B. Gladwin T. Hillegers M. Hoogendam J. M. van den Wildenberg W. P. (2014). Frontostriatal activity and connectivity increase during proactive inhibition across adolescence and early adulthood. *Hum. Brain Mapp.* 35 4415–4427. 10.1002/hbm.22483 24532023 PMC6869143

[B84] Wager T. D. Jonides J. Reading S. (2004). Neuroimaging studies of shifting attention: a meta-analysis. *Neuroimage* 22 1679–1693. 10.1016/j.neuroimage.2004.03.052 15275924

[B85] Wager T. D. Lindquist M. Kaplan L. (2007). Meta-analysis of functional neuroimaging data: current and future directions. *Soc. Cogn. Affect. Neurosci.* 2 150–158. 10.1093/scan/nsm015 18985131 PMC2555451

[B86] Wager T. D. Sylvester C. Y. Lacey S. C. Nee D. E. Franklin M. Jonides J. (2005). Common and unique components of response inhibition revealed by fMRI. *Neuroimage* 27 323–340. 10.1016/j.neuroimage.2005.01.054 16019232

[B87] Wang Y. Braver T. S. Yin S. Hu X. Wang X. Chen A. (2019). Reward improves response inhibition by enhancing attentional capture. *Soc. Cogn. Affect. Neurosci.* 14 35–45. 10.1093/scan/nsy111 30535116 PMC6318467

[B88] Williams B. R. Ponesse J. S. Schachar R. J. Logan G. D. Tannock R. (1999). Development of inhibitory control across the life span. *Dev. Psychol.* 35 205–213. 10.1037//0012-1649.35.1.205 9923475

[B89] Wright L. Lipszyc J. Dupuis A. Thayapararajah S. W. Schachar R. (2014). Response inhibition and psychopathology: a meta-analysis of go/no-go task performance. *J. Abnorm. Psychol.* 123 429–439. 10.1037/a0036295 24731074

[B90] Yanaka H. T. Saito D. N. Uchiyama Y. Sadato N. (2010). Neural substrates of phasic alertness: a functional magnetic resonance imaging study. *Neurosci. Res.* 68 51–58. 10.1016/j.neures.2010.05.005 20561955

[B91] Yeo B. T. Krienen F. M. Sepulcre J. Sabuncu M. R. Lashkari D. Hollinshead M. (2011). The organization of the human cerebral cortex estimated by intrinsic functional connectivity. *J. Neurophysiol.* 106 1125–1165. 10.1152/jn.00338.2011 21653723 PMC3174820

[B92] Zacks R. T. Hasher L. Li K. Z. H. (2000). Human Memory. In Craik F. I. M. Salthouse T. A. (Eds.) *The Handbook of Aging and Cognition*, 2nd Edn. Mahwah, NJ: Lawrence Erlbaum Associates Publishers, 293–357.

[B93] Zahodne L. B. Zajacova A. (2020). Education and Cognitive Aging: an Introduction to the Special Section. *J. Gerontol. B. Psychol. Sci. Soc. Sci.* 75:e78–e80. 10.1093/geronb/gbaa091 32716028

[B94] Zandbelt B. B. van Buuren M. Kahn R. S. Vink M. (2011). Reduced proactive inhibition in schizophrenia is related to corticostriatal dysfunction and poor working memory. *Biol. Psychiatry* 70 1151–1158. 10.1016/j.biopsych.2011.07.028 21903198

[B95] Zhang F. Iwaki S. (2019). Common Neural Network for Different Functions: an Investigation of Proactive and Reactive Inhibition. *Front. Behav. Neurosci.* 13:124. 10.3389/fnbeh.2019.00124 31231199 PMC6568210

[B96] Zhang R. Geng X. Lee T. M. C. (2017). Large-scale functional neural network correlates of response inhibition: an fMRI meta-analysis. *Brain Struct. Funct.* 222 3973–3990. 10.1007/s00429-017-1443-x 28551777 PMC5686258

