# Supplementary material for: Distinct neural activation patterns of age in subcomponents of inhibitory control: A fMRI meta-analysis
Source: Front Aging Neurosci. 2022 Aug 5;14:938789. doi: 10.3389/fnagi.2022.938789 (PMC9389163; doi:10.3389/fnagi.2022.938789)
Supplement: Supplementary file 1 [file Presentation_1.pdf]

Supplementary material.

Distinct neural activation patterns of age in subcomponents of inhibitory control: a fMRI meta-analysis

Jixin Long[1], Xiaoqi Song[1], You Wang[1,2], Chanyu Wang[1], Ruiwang Huang[3], Ruibin Zhang[1,2]*

1. Cognitive control and Brain Healthy Laboratory, Department of Psychology, Southern Medical University, Guangzhou, China
2. Department of Psychiatry, Zhujiang Hospital, Southern Medical University, Guangzhou, China
3. School of Psychology, South China Normal University, Guangzhou, China

*** Corresponding author:**

Ruibin Zhang, Cognitive control and Brain Healthy Laboratory Department of Psychology, School of Public Health, Southern Medical University. Email: ruibinzhang@foxmail.com

Contents

Table S1 Information on source datasets included in the meta-analysis 3
Table S2 Brain activation in all included inhibitory control experiments 15
Table S3 Brain activation differences among four age groups for tasks tapping cognitive inhibition only 16
Table S4 Brain activation differences among four age groups for tasks tapping response inhibition only 17
Table S5 Brain activation differences between cognitive inhibition and 54 contrasts for response inhibition 18
Table S6 Activation regions of leave-one-out analysis in cognitive inhibition tasks 19
Table S7 Activation regions of leave-one-out analysis in response inhibition tasks 20
Table S8 Brain activation in successful inhibition for tasks tapping response inhibition only 21
Table S9 Brain activation differences among four age groups for tasks tapping successful response inhibition 22
Figure S1 Number of studies investigating the development of inhibitory control across life span (1 Jan. 2000–31 Mar. 2020). 24
Figure S2 Brain activation differences between cognitive inhibition and randomly selected 54 contrasts for response inhibition. 25
Figure S3 Activation maps between leave-one-out analysis and full tasks of each subcomponents in cognitive and response inhibition. 26
Figure S4 Activation maps displaying whole brain regression analysis in response inhibition and successful inhibition with age as a covariate. 27
Figure S5 Brain activation differences among four age groups for tasks tapping successful response inhibition. 28

Table S1 Information on source datasets included in the meta-analysis

Study	N(Male)	age range	Mean Age	Control Types	Paradigm	Stimulation Type	Contrast	Foci
Poirel et al. (2012)	28(12)	NA	7.71	CI	Stroop	Picture	surpass	10
Sheridan et al. (2014)	33(19)	5.7-10.7	8.1	CI	Simon	Picture	Incongruent>congruent	9
Bunge et al. (2002)	16(9)	8-12	10	CI	Flanker	Arrow	Incongruent>Neutral	5
Bunge et al. (2002)	16(9)	8-12	10	CI	Flanker	Arrow	NoGo>Neutral	2
Fitzgerald et al. (2008)	11(6)	8-14	11.5	RI	Antisaccade	Picture	Antisaccade	12
Bernal & Altman (2009)	18(8)	NA	14.67	CI	Stroop	Word	Incongruent - rest	44
Rubia et al. (2006)	29(29)	10-17	15	CI	Simon Task	Picture	Incongruent-Congruent	5
Velanova et al. (2009)	78	8-27	15.62	RI	Antisaccade	Picture	correct AS	5
Padmanabhan et al. (2011)	30(12)	8-25	15.8	RI	Antisaccade	Picture	NA	12
Hansen et al. (2018)	171(121)	NA	16.2	CI	Stroop	Word	Incongruent>Congruent	17
Halari et al. (2009)	21(10)	NA	16.3	CI	Simon	Picture	incongruent>congruent	6
Andrews-Hanna et al. (2011)	65	14-25	18.8	CI	Stroop	Word	Incongruent> Congruent	17
Anderson et al. (2016)	18(10)	18-22	19.9	CI	Flanker	Word	Incongruent	11
Huang et al. (2017)	33(18)	NA	20	CI	Stroop	Word	Incongruent>Neutral	5
Jiang et al. (2016)	24(14)	19-24	21	CI	Stroop	Word	Incongruent > congruent	13
Veroude et al. (2013)	74(35)	18.36-25.95	21.47	CI	Stroop	Word	Incongruent>Neutral	17
Wager et al. (2005)	14	18-25	21.5	CI	Flanker	Picture	Incongruent	9
Yang et al. (2018)	30(10)	18-25	21.65	CI	Flanker	Picture	Incongruent > fixation	31
Fernandez-Ruiz et al. (2018)	25(11)	18-25	21.7	RI	Antisaccade	Picture	Antisaccade	18
Song & Hakoda (2015)	20(10)	18-27	21.7	CI	Stroop	Word	Incongruent>Congruent	20

Grandjean et al. (2012)	25(12)	18-29	21.8	CI	Stroop	Word	Incongruent>neutral	19
Zurawska Vel Grajewska et al. (2011)	66(15)	19-34	22.35	CI	Flanker	Letter	Homogeneous>conflict	26
Korsch et al. (2014)	20(10)	NA	22.95	CI	Flanker/SRC	Arrow	Incongruent>Congruent	4
Berron et al. (2015)	24(11)	20-29	23.3	CI	Flanker	Arrow	Incongruent>Congruent	10
Mitchell (2005)	15(4)	NA	23.3	CI	Stroop	Word	Incongruent>neutral	23
Brass et al. (2001)	10(4)	20-26	23.5	CI	Stroop	Video	Incongruent-Congruent	5
King et al. (2012)	25(11)	18-33	23.8	CI	Flanker	Face	Incongruent>Congruent	9
McNab et al. (2008)	11(4)	22-34	24	CI	Flanker	Arrow	Incongruent >Congruent	5
Konishi et al. (2011)	78(35)	20-28	24	CI	WCST	Picture	Control-release	22
Bunge et al. (2002)	16(9)	19-33	24	CI	Flanker	Arrow	Incongruent>Neutral	11
Bunge et al. (2002)	16(9)	19-33	24	CI	Flanker	Arrow	NoGo>Neutral	16
Wagner et al. (2013)	34(16)	18-51	24.1	CI	Stroop	Word	Incongruent>congruent	8
Forstmann et al. (2008)	24(9)	NA	24.2	CI	Simon	Letter	Incongruent>Neutral	6
Sebastian et al. (2013b)	21(12)	NA	24.23	CI	Stroop	Letter	Incongruent >congruent	24
Sebastian et al. (2013b)	21(12)	NA	24.23	RI	GNG	Arrow	No-go>congruent go	17
Sebastian et al. (2013b)	21(12)	NA	24.23	RI	SST	Arrow	Stop>congruent go	24
Chikazoe et al. (2007)	24(12)	20-29	24.5	RI	Antisaccade	Picture	Antisaccade>Control	53
Mayer et al. (2012)	26(13)	NA	24.88	CI	Stroop	Sound	Incongruent>congruent	17
Reuter et al. (2010)	19	NA	24.9	RI	saccade	Picture	Inhibition	7
Iannaccone et al. (2015)	15(7)	NA	25.4	CI	Flanker	Picture	Conflict correct	13
Kim et al. (2011)	13(8)	19-32	25.5	CI	Stroop	Word	Incongruent>neutral	7
Wittfoth et al. (2006)	20(3)	21-31	25.5	CI	Simon	Picture	Incompatible > compatible	38
Marco-Pallarés et al. (2008)	34(12)	NA	25.8	CI	Flanker	Letter	Inhibited>Responses	11
Brass et al. (2005)	20(8)	21-37	26	CI	Stroop	Video	Incongruent>Congruent	7

Brown et al. (2006a)	10(4)	22-33	26	RI	prosaccIade	Picture	Pro-response	23
Brown et al. (2006a)	10(4)	22-33	26	RI	antisaccade	Picture	Anti-response	24
Enriquez-Geppert et al. (2010)	19	19-36	26	CI	picture-word interference task	Picture	Response suppression	23
Marsh et al. (2006)	70(34)	NA	26.7	CI	Stroop	Word	Control	11
Agam et al. (2010)	14(8)	NA	27	RI	Antisaccade	Picture	Antisaccade	7
Sebastian et al. (2013b)	24(9)	NA	27.42	CI	Simon Task	Arrow	Incongruent >Congruent	22
Tu et al. (2006)	10(5)	NA	27.9	RI	Antisaccade	Picture	Antisaccade	21
Rubia et al. (2006)	23(23)	20-43	28	CI	Simon Task	Picture	Incongruent>Congruent	9
Potenza et al. (2003)	11	NA	29	CI	Stroop	Picture	Incongruent	11
Wingenfeld et al. (2009)	14(6)	NA	29.45	CI	Stroop	Word	Incongruent>neutral	25
Hakvoort Schwerdtfeger et al. (2012)	14(9)	NA	29.6	RI	Antisaccade	Picture	Antisaccade	13
Jaffard et al. (2008)	14(14)	23-37	30	CI	Simon	Picture	withholding movement	7
Sebastian et al. (2012)	24(11)	NA	30.3	CI	Simon Task	Arrow	Incongruent >Congruent	10
Liu et al. (2004)	11(3)	24-40	32	CI	Simon Stroop	Arrow	Incongruent > Congruent	49
Page et al. (2009)	11(11)	NA	34.1	CI	Stroop	Arrow	Stroop>Baseline	6
Wagner et al. (2006)	16(0)	NA	38.8	CI	Stroop	Word	Incongruent>congruent	2
Schmitz et al. (2006)	12(12)	NA	39	CI	Stroop	Picture	Correct Stroop	5
Matsuda et al. (2004)	21	NA	39.2	RI	Antisaccade	Picture	Antisaccade>rest	29
Sebastian et al. (2013a)	49(19)	20-77	39.96	CI	Simon Task	Arrow	Success inhibition	10
Schulte et al. (2012)	17(17)	NA	50	CI	Stroop	Word	Incongruent>Congruent	4
Fernandez-Ruiz et al. (2018)	25(10)	49-83	66.2	RI	Antisaccade	Picture	Antisaccade	18
Korsch et al. (2014)	19(10)	NA	70.26	CI	Flanker/SRC	Arrow	Incongruent>Congruent	6
Janssen et al. (2015)	17(13)	NA	10.28	RI	SST	Picture	Successful Inhibition	4

Booth et al. (2003)	12(7)	9.3-11.7	10.9	RI	GNG	Picture	NoGo>Go	16
Bruce et al. (2013)	11(6)	9-12	10.98	RI	GNG	Picture	NoGo>Go	7
Lei et al. (2012)	22(13)	8-15	11.5	RI	GNG	Letter	NoGo>Go	14
Bennett et al. (2009)	11(5)	NA	12.6	RI	GNG	Letter	NoGo>Go	8
Todd et al. (2012)	44(15)	4-38	13.26	RI	GNG	Face	NoGO	20
Carrion et al. (2008)	14(6)	NA	13.3	RI	GNG	Letter	NoGo>Go	31
Liu et al. (2016)	76(40)	8-19	13.9	RI	MSIT	Digit	NA	7
Passarotti et al. (2010)	15(7)	NA	14.13	RI	SST	Picture	Stop>Go	5
Bernal & Altman (2009)	15	NA	14.2	RI	Motor Inhibition	Word	Inhibition	40
Singh et al. (2010)	22(13)	NA	14.3	RI	GNG	Arrow	NoGo-Go	2
Tamm et al. (2002)	19(8)	8-20	14.41	RI	GNG	Letter	NoGo>Go	4
Ware et al. (2015)	21(12)	13-16	14.5	RI	SST	Letter	Stop>Go	38
Bartholdy et al. (2019)	43(3)	NA	14.61	RI	SST	Arrow	Stop>Go	1
Stevens et al. (2007)	50	11-17	14.7	RI	GNG	Letter	response inhibition	60
Rubia et al. (2006)	29(29)	10-17	15	RI	GNG	Arrow	NoGo>Go	4
Roos et al. (2017)	7(3)	NA	15	RI	GNG	Digit	NoGo>Go	4
Bhaijiwala et al. (2014)	12	9-18	15.4	RI	SST	Letter	Stop>Go	11
Tamm et al. (2004)	12(12)	14-16	15.58	RI	GNG	Letter	NoGo	3
Lock et al. (2011)	13	NA	15.93	RI	GNG	Letter	NoGo>Go	7
Qiao et al. (2016)	17(17)	12-18	15.94	RI	GNG	Letter	NoGo>Go	14
Cope et al. (2020)	117	7-13	16.26	RI	GNG	Letter	Correct reject>base	20
Feldstein Ewing et al. (2015)	95(77)	14-18	16.29	RI	GNG	Letter	NoGo>Go	13
Halari et al. (2009)	21(10)	NA	16.3	RI	SST	Arrow	Succ Inhibition	7
Ordaz et al. (2013)	123(59)	9-26	16.65	RI	GNG	Letter	NoGo>Go	13
Schel et al. (2014b)	43(20)	10-26	17.1	RI	GNG	Picture	NoGo>Go	14

Schulz et al. (2004)	9	16.1-19.9	17.5	RI	GNG	Letter	NoGo>Go	5
Suárez-Suárez et al. (2020)	35(16)	NA	18.08	RI	GNG	Picture	NoGo>Go	8
Shafritz et al. (2015)	15(12)	12-26	18.4	RI	GNG	Letter	NoGo>Go	24
Smith et al. (2013)	20(7)	NA	18.7	RI	GNG	Letter	NoGo	19
Cohen-Gilbert et al. (2017)	23	18-20	18.8	RI	GNG	Picture	NoGo	15
Galván et al. (2011)	25(14)	16-21	19	RI	SST	Arrow	SuccStop-Go	22
Braet et al. (2009)	40(35)	10-35	19.6	RI	GNG	Digit	Successful Inhibition	23
Vink et al. (2014)	20(10)	NA	20	RI	SST	Bar	Stop>Go	8
Strakowski et al. (2008)	16(9)	NA	20	RI	SST	Letter	Increased activation	14
Lawrence et al. (2009)	21(9)	NA	20.13	RI	GNG	Arrow	NoGo>Oddball	3
Wang et al. (2019)	27(9)	18-23	20.5	RI	SST	Arrow	Stop>Go	19
Wang et al. (2019)	27(9)	18-23	20.5	RI	SST	Arrow	Stop>Continue	4
Longo et al. (2013)	13(6)	19-26	21	RI	GNG	Letter	inhibition>control	17
Lenartowicz et al. (2011)	26(11)	NA	21.3	RI	SST	Face	go/stop-stop > go/stop-go	7
Lenartowicz et al. (2011)	26(11)	NA	21.3	RI	SST	Face	go-stop > go/stop-go	2
Schel et al. (2014a)	24(11)	18-26	21.49	RI	marble task	Picture	NoGo>Go	23
Schel et al. (2014a)	24(11)	18-26	21.49	RI	SST	Picture	SuccStop>SuccGo	13
Chuah et al. (2006)	27(15)	19-26	21.5	RI	GNG	Letter	Stop	5
Sagaspe et al. (2011)	14	18-25	21.5	RI	SST	Face	Stop>Go	24
Wager et al. (2005)	14	18-25	21.5	RI	GNG	Picture	NoGo	13
Vanderhasselt et al. (2011)	34(9)	NA	21.56	RI	GNG	Face	NoGo>Null	8
Majid et al. (2013)	18(8)	NA	21.6	RI	SST	Picture	Stop>Go	20
Berkman et al. (2014)	60(27)	18-30	21.63	RI	SST	Arrow	Stop>Go	31
Rubia et al. (2013)	66(41)	13-45	22	RI	SST	Arrow	Succ inhibition	4
Padmala & Pessoa (2010)	35(16)	NA	22	RI	SST	Picture	SuccStop>UnSuccStop	14

Padmala & Pessoa (2010)	35(16)	NA	22	RI	SST	Picture	SuccStop>UnSuccStop	12
Lemire-Rodger et al. (2019)	22(11)	18-28	22.14	RI	GNG	Picture	inhibition>control	10
Zandbelt & Vink (2010)	24(6)	19-26	22.2	RI	SST	Bar	SuccStop>Go	71
Zandbelt & Vink (2010)	24(6)	19-26	22.2	RI	SST	Bar	SuccStop>UnSuccStop	14
Chikazoe et al. (2009b)	22(10)	NA	22.3	RI	SST	Picture	Stop>Go	57
Brown et al. (2012)	20(7)	18-28	22.5	RI	GNG	Picture	NoGo>Go	17
Boehler et al. (2014)	16(1)	19-24	22.8	RI	SST	Picture	SuccStop>Go	29
Boehler et al. (2010)	15(6)	NA	22.9	RI	SST	Picture	Stop>Go	50
O'Connor et al. (2012)	18(9)	NA	23	RI	GNG	Digit	Succ NoGo	19
Jimura et al. (2014)	46(26)	20-26	23	RI	SST	Picture	SuccStop>Go	17
Hester et al. (2009)	16(6)	19-42	23	RI	GNG	Letter	correct response inhibition	10
Behan et al. (2015)	20(9)	18-35	23.05	RI	GNG	Picture	NoGo success	3
Pornpattananangkul et al. (2016)	58(29)	NA	23.18966	RI	GNG	Letter	Go/NoGo > Go	10
Chiang et al. (2013)	16(6)	19-34	23.5	RI	GNG	Picture	NoGo>Go	15
Chikazoe et al. (2009a)	25(10)	20-27	23.5	RI	GNG	Picture	NoGo>Go	104
Leung & Cai (2007)	12(6)	19-28	23.5	RI	SST	Picture	Stop>Go	15
Jahfari et al. (2011)	20(9)	18-33	23.55	RI	SST	Picture	SuccStop>Go	7
Nakata et al. (2008)	15(7)	19-32	23.6	RI	GNG	Pulse	NoGo>Go	104
Schulz et al. (2011)	16(8)	18-35	23.6	RI	GNG	Arrow	Inhibition	8
Costa et al. (2013)	50(50)	18-30	23.65	RI	GNG	Picture	Succ>Unsucc	3
Costa et al. (2013)	42(42)	18-30	23.65	RI	SST	Picture	Succ>Unsucc	17
Ness & Beste (2013)	13(5)	19-31	23.69	RI	SST	Picture	Stop>Go	13
Rothmayr et al. (2011)	12(5)	23-24	23.7	RI	GNG	Picture	NoGo>Go	4
Fassbender et al. (2006)	16(5)	20-30	23.7	RI	GNG	Letter	correct inhibitions	18

Lavallee et al. (2014)	37(20)	NA	23.8	RI	GNG	Picture	NoGo>Go	9
Goldstein et al. (2007)	14(4)	18-31	23.9	RI	GNG	Word	NoGo>Go	30
McNab et al. (2008)	11(4)	22-34	24	RI	GNG	Picture	NoGo>Go	23
McNab et al. (2008)	11(4)	22-34	24	RI	SST	Arrow	Stop>Go	41
Jahfari et al. (2012)	16(5)	21-32	24.1	RI	SST	Picture	SuccStop>Go	8
Ko et al. (2014)	23(23)	NA	24.35	RI	GNG	Digit	NoGo>Go	1
Laurens et al. (2005)	10(5)	NA	24.4	RI	GNG	Letter&Sound	NoGo>Go	12
Chiu & Egner (2015)	24(14)	NA	24.4	RI	GNG	Face	NoGo>Go	8
Chen et al. (2015)	15	NA	24.47	RI	GNG	Digit	NoGo>Go	7
Del-Ben et al. (2005)	12(12)	19-36	24.7	RI	GNG	Letter	NoGo>Go	15
Scalzo et al. (2016)	21(8)	17.7-33.5	24.7	RI	GNG	Picture	Succ No>Go	20
Huster et al. (2010)	22(11)	20-28	24.7	RI	GNG	Tactile	NoGo	9
Baumeister et al. (2014)	17(9)	20-35	24.71	RI	Flanker/GNG	Arrow	NoGo>neutral	16
Boecker et al. (2011)	15(15)	NA	24.8	RI	SST	Picture	stopinhibit>go	2
Jamadar et al. (2010)	18(7)	NA	25	RI	GNG	Letter&Digit	NoGo>Go	43
Coxon et al. (2016)	20(9)	20-31	25	RI	SST	Bar	stopinhibit>go	28
Liu et al. (2012)	28(13)	20-38	25	RI	GNG	Letter	Correct NoGo	11
Watanabe et al. (2002)	11(9)	19-40	25	RI	GNG	Picture	NoGo	9
Zhang et al. (2012b)	25(10)	19-52	25	RI	SST	Picture	Stop>Go	1
Booth et al. (2003)	12(5)	20.6-30.9	25.1	RI	GNG	Picture	NoGo>Go	13
Asahi et al. (2004)	17(10)	23-30	25.1	RI	GNG	Letter	NoGo>Go	11
Hare et al. (2005)	10(5)	NA	25.2	RI	GNG	Face	NoGo>Go	6
Konishi et al. (1999)	6(5)	20-31	25.5	RI	GNG	Picture	NoGo>Go	5
Doallo et al. (2012)	12(4)	20-31	25.5	RI	GNG	Face	NoGo	18

Berkman et al. (2009)	14(6)	21-34	25.6	RI	GNG	Picture	NoGo>Go	21
Campanella et al. (2017)	17(7)	NA	25.8	RI	GNG	Letter	Correct Inhibition	17
Maguire et al. (2003)	6(6)	22-30	26	RI	GNG	Picture	Go/NoGo>Go	16
Brown et al. (2006a)	10(4)	22-33	26	RI	GNG	Picture	NoGo>response	20
Sebastian et al. (2016)	28(11)	21-47	26.1	RI	SST	Arrow	Stop>Go	13
Enriquez-Geppert et al. (2010)	15(5)	NA	26.2	RI	GNG	Picture	NoGo	20
Enriquez-Geppert et al. (2010)	15(5)	NA	26.2	RI	SST	Picture	Stop	32
Goghari & MacDonald (2009)	12(7)	NA	26.2	RI	GHG	Picture&Word	NoGo>Go	18
Ramautar et al. (2006)	16(8)	20-33	26.25	RI	SST	Picture	SuccStop>NoStop	7
Borgwardt et al. (2008)	15(15)	20-42	26.7	RI	GNG	Picture	NG>Oddball	5
Brevers et al. (2017)	16(6)	NA	26.87	RI	SST	Picture	SucceStop>UnsuccStop	16
Zhang et al. (2012a)	18(5)	20-39	27	RI	GNG	Picture	NoGo	8
Cai & Leung (2009)	12(6)	18-36	27	RI	SST	Picture	Stop>Go	25
de Zubicaray et al. (2000)	8(8)	NA	27	RI	GNG	Picture	NoGo>Go	11
Hughes et al. (2014)	12(6)	19-41	27.25	RI	SST	Letter	Stop>Go	15
Goya-Maldonado et al. (2010)	21(10)	NA	27.4	RI	GNG	Picture	NoGo>Go	2
Kolodny et al. (2017)	20(7)	NA	27.4	RI	GNG	Picture	NoGo>Go	9
Sebastian et al. (2013b)	24(9)	NA	27.42	RI	GNG	Arrow	NoGo>Go	25
Sebastian et al. (2013b)	24(9)	NA	27.42	RI	SST	Arrow	Stop>go	22
Tabu et al. (2011)	13(8)	NA	27.5	RI	SST	Picture	SuccStop>Go	6
Hughes et al. (2013)	15(8)	22-34	27.5	RI	SST	Letter	Signal inhibit>baseline	4
Hughes et al. (2013)	15(8)	22-34	27.5	RI	SST	Letter	Signal inhibit>respond	2

Walther et al. (2010)	17(8)	23-33	27.5	RI	GNG	Picture&Sound	NoGo	15
Rae et al. (2015)	16(12)	20-38	28	RI	SST	Arrow	Stop correct>Go	63
Durston et al. (2002)	10(5)	NA	28	RI	GNG	Picture	NoGo>Go	10
Rubia et al. (2006)	23(23)	20-43	28	RI	GNG	Arrow	NoGo>Go	11
Cai et al. (2014)	23(11)	19-37	28	RI	SST	Picture	Stop>Go	33
Karoly et al. (2014)	53(25)	21-53	28.3	RI	SST	Picture	correct reject>go	8
Dambacher et al. (2014)	17(17)	NA	28.4	RI	GNG	Letter	NoGo>Go	13
Dambacher et al. (2014)	17(17)	NA	28.4	RI	SST	Letter	Stop>Go	20
Drueke et al. (2013)	14(14)	18-39	28.5	RI	SST	Picture	stopinhibit>go	16
Cai & Leung (2011)	26(15)	18-39	28.5	RI	SST	Picture	Stop>Go	50
Blasi et al. (2006)	57(32)	NA	28.6	RI	GNG	Arrow	NoGo>Con/NoGo>NEU	16
Garavan et al. (2006)	71(26)	18-46	29	RI	GNG	Letter	NoGo>Go	20
Aron & Poldrack (2006)	13(9)	NA	29.2	RI	SST	Arrow	stopinhibit>go	35
Chevrier et al. (2007)	14(8)	22-35	29.4	RI	SST	Letter	Success Stop	3
Mehren et al. (2019)	20(16)	NA	29.5	RI	GNG	Letter	Correct NoGo	16
Fu et al. (2008)	18(18)	23-44	29.59	RI	GNG	Letter	NoGo>Go	22
Burke & Barnes (2011)	11(5)	NA	29.7	RI	GNG	Picture	NoGo>Rnd	17
Mulligan et al. (2011)	12(12)	NA	29.9	RI	GNG	Letter	NoGo>Go	11
Mulligan et al. (2011)	12(12)	NA	29.9	RI	GNG	Letter	NoGo>0	9
Fassbender et al. (2009)	15(5)	23-40	30	RI	GNG	Letter	correct inhibition	3
Kelly et al. (2004)	15(5)	23-40	30	RI	GNG	Letter	Successful inhibition	23
Garavan et al. (2002)	14(4)	19-45	30	RI	GNG	Letter	Stops	16
Hester et al. (2004)	15(5)	23-40	30	RI	GNG	Letter	Successful inhibition	21
Liddle et al. (2001)	16(9)	NA	30.2	RI	GNG	Letter	NoGo>baseline	19
Liddle et al. (2001)	16(9)	NA	30.2	RI	GNG	Letter	NoGo>Go	23
Sebastian et al. (2012)	24(11)	NA	30.3	RI	GNG	Arrow	NoGo>Go	19

Sebastian et al. (2012)	24(11)	NA	30.3	RI	SST	Arrow	Stop>Go	28
Tabu et al. (2012)	13(11)	NA	30.7	RI	SST	Picture	SuccStop>Go	8
Gavazzi et al. (2019)	36(15)	NA	30.75	RI	GNG	Letter	NoGo>Go	18
Congdon et al. (2014)	62(32)	NA	30.82	RI	SST	Arrow	Stop-inhibition>Go	4
Zheng et al. (2008)	20(8)	22-40	31	RI	SST	Picture	Succ-inhibition>Go	10
Zheng et al. (2008)	20(8)	22-40	31	RI	GNG	Picture	Succ-inhibition>Go	8
Daly et al. (2014)	14(14)	NA	31	RI	GNG	Arrow	NoGo > Oddball	17
Garavan et al. (1999)	14(8)	NA	31	RI	GNG	Letter	NoGO	14
Altshuler et al. (2005)	13(5)	NA	31	RI	GNG	Letter	NoGo>Go	4
Criaud et al. (2017)	20(10)	20-42	31	RI	GNG	Picture	(nogo+go) - (go_control)	21
Bellgrove et al. (2004)	42(13)	18-46	31	RI	GNG	Letter	Success Inhibition	19
Hester & Garavan (2004)	15(7)	20-40	31	RI	GNG	Letter	Success inhibition	64
White et al. (2014)	123(66)	NA	31.14	RI	SST	Arrow	Stop Inhibit>Go	17
Mobbs et al. (2007)	11(2)	15.5-48.8	31.4	RI	GNG	Letter	NoGo>Go	12
Hu et al. (2018)	149(66)	18-72	31.6	RI	SST	Picture	Stop>Go	11
Hendrick et al. (2010)	60(30)	22-42	32	RI	SST	Word	Stop>Go	18
Li et al. (2006)	24(18)	22-42	32	RI	SST	Picture	SuccSTop-FailStop	9
Li et al. (2008)	40(20)	22-42	32	RI	SST	Picture	SuccStop>Go	2
Barkataki et al. (2008)	14(14)	18-45	32.14	RI	GNG	Picture	NoGo>Go	1
Wang et al. (2018)	116(59)	NA	32.3	RI	SST	Picture	Stop>Go	20
Vercammen et al. (2012)	23(11)	NA	33.3	RI	GNG	Word	Inhibit negative >neutral	11
Li et al. (2008)	30(30)	22-45	33.5	RI	SST	Picture	SuccStop	8
Duerden et al. (2013)	20(15)	20.8-43.4	33.7	RI	GNG	Face	NoGo>Go	13
Crane et al. (2016)	54(16)	NA	33.8	RI	GNG	Letter	Correct response inhibition	10

Steele et al. (2013)	102(49)	23-52	33.92	RI	GNG	Letter	Correct Rejection>Hits	36
Horn et al. (2003)	21(21)	18-50	34	RI	GNG	Letter	NoGo>Go	14
Sharp et al. (2010)	26(17)	23-59	34	RI	SST	Arrow	Stop correct>Go	10
Page et al. (2009)	11(11)	NA	34.1	RI	GNG	Arrow	NoGo>Baseline	11
Langenecker et al. (2007)	22(8)	NA	34.2	RI	GNG	Letter	Correct Rejection	8
Welander-Vatn et al. (2013)	24(13)	NA	34.5	RI	GNG	Letter	NoGo	19
Kaladjian et al. (2009b)	20(10)	NA	34.6	RI	GNG	Letter&Picture	Correct NoGo>Go	16
Mazzola-Pomietto et al. (2009)	16(6)	NA	34.6	RI	GNG	Letter	NoGo>Go	7
Bannbers et al. (2013)	13(0)	NA	34.9	RI	GNG	Letter	Correct NoGo	5
Roth et al. (2007)	14(6)	NA	34.9	RI	GNG	Picture	NoGo	13
Hughes et al. (2012)	10(7)	NA	35.1	RI	SST	Letter	Stop>Baseline	5
Kaladjian et al. (2007)	21(19)	NA	35.7	RI	GNG	Letter&Picture	NoGo>Go	11
Rubia et al. (2001)	15(15)	26-58	36	RI	SST	Arrow	Stops	6
Rubia et al. (2001)	15(15)	26-58	36	RI	GNG	Arrow	NoGo	12
Le et al. (2020)	72(36)	21-74	36.4	RI	GNG	Picture	NoGo success	5
Kärgel et al. (2017)	40(26)	20-57	36.65	RI	GNG	Letter	NoGo>Go	16
Townsend et al. (2012)	30(17)	NA	37	RI	GNG	Letter	NoGo	24
Rosell-Negre et al. (2014)	28(23)	20-56	38.89	RI	SST	Letter	Stop>Go	9
Schmitz et al. (2006)	12(12)	NA	39	RI	GNG	Picture	Correct NoGo	11
Falconer et al. (2008)	23(10)	21-68	39.3	RI	GNG	Word	NoGo>Go	6
Karch et al. (2008)	16	NA	39.3	RI	GNG	Sound	NoGo>Control	13
Sebastian et al. (2013a)	49(19)	20-77	39.96	RI	GNG	Arrow	no-go>go	10
Sebastian et al. (2013a)	49(19)	20-77	39.96	RI	SST	Arrow	Stop>go	10
Rubia et al. (2001)	40	26-58	40	RI	SST	Arrow	Stop	5

Rubia et al. (2001)	7	26-58	40	RI	GNG	Arrow	NoGo	10
van der Salm et al. (2013)	48(22)	NA	40.7	RI	SST	Letter	Stop>Inhibit	9
Kaladjian et al. (2009a)	10(5)	NA	41.5	RI	GNG	Letter&Picture	NoGo>Go	20
Czapla et al. (2017)	21(17)	NA	41.95	RI	GNG	Picture	NoGo>Go	9
Brown et al. (2006b)	58(21)	NA	45.3	RI	GNG	Face	NoGo>Go	5
Berkman et al. (2012)	31(16)	NA	46	RI	GNG	Letter	NoGo>Go	18
Habermeyer et al. (2013)	7(7)	35-61	47	RI	GNG	Letter	NoGo > Go	16
Sjoerds et al. (2014)	16(10)	NA	47.1	RI	SST	Picture	SuccStop>Go	22
Nielson et al. (2004)	28(14)	19-77	50.4	RI	GNG	Letter	inhibition	34
Langenecker & Nielson (2003)	22(7)	NA	50.45	RI	GNG	Letter	NoGo	23
Nielson et al. (2002)	34(14)	18-78	52.3	RI	GNG	Letter	NoGo	43
Weywadt et al. (2017)	38(11)	41-75	61	RI	GNG	Letter	Correct Rejects > Hits	17
Bobb et al. (2012)	13(4)	55-85	62	RI	SST	Arrow	Stop>Go	18
Manza et al. (2018)	37(21)	NA	62.5	RI	SST	Picture	Stop>Go	3
Baglio et al. (2011)	11(4)	NA	66.9	RI	GNG	Letter	NoGo>baseline	5
Bloemendaal et al. (2018)	24(15)	61-72	67.5	RI	SST	Bar	StopSuccess > Go	12
Coxon et al. (2016)	20(9)	62-81	68.7	RI	SST	Bar	stopinhibit>go	4

N = number of subjects; CI = cognitive inhibition; RI = response inhibition; GNG = Go/NoGo task; SST = stop signal task

Table S2 Brain activation in all included inhibitory control experiments

Regions	R/L	MNI			No.Voxs	Maximum P
		x	y	z		
Angular Gyrus	R	30	-60	46	1882	0.37
Inferior Frontal Gyrus	L	-38	30	2	632	0.36
Inferior Frontal Gyrus	L	-44	16	22	1655	0.37
Inferior Frontal Gyrus	R	42	28	28	1692	0.42
Inferior Parietal Lobule	R	48	-44	40	2381	0.4
Inferior Parietal Lobule	L	-38	-52	44	2963	0.31
Insula	R	40	20	-2	4377	0.53
Insula	L	-34	14	-2	2039	0.41
Middle Cingulate Cortex	R	4	28	34	1850	0.38
Middle Cingulate Cortex	R	2	-26	38	54	0.18
Middle Frontal Gyrus	R	34	46	22	1714	0.32
Middle Frontal Gyrus	L	-36	38	26	584	0.21
Middle Frontal Gyrus	R	28	0	56	1691	0.31
Middle Temporal Gyrus	R	56	-42	12	2548	0.34
Occipital Gyrus	L	-40	-66	-8	835	0.22
Occipital Gyrus	R	40	-70	-6	343	0.2
Precentral Gyrus	R	46	8	32	2278	0.42
Precentral Gyrus	L	-32	0	50	1164	0.24
Precuneus	R	14	-68	46	407	0.27
Superior Temporal Gyrus	L	-14	4	6	1768	0.31
Superior Temporal Gyrus	R	12	-2	6	2005	0.33
Superior Temporal Gyrus	R	4	-14	26	83	0.2
Supplementary Motor Area	R	0	10	52	3485	0.42
SupraMarginal Gyrus	L	-56	-46	24	800	0.24

Maximum P is the maximum proportion of studies exhibiting the effect at the peak density weighted by sample size. The coordinates are Montreal Neurological Institute (MNI) standard stereotaxic spaces. The voxel size is $2 \times 2 \times 2mm^3$. R/L: right/left hemisphere

Table S3 Brain activation differences among four age groups for tasks tapping cognitive inhibition only

Regions	R/L	MNI			No.Voxs	Maximum P
		x	y	z		
Young > Middle-aged						
Inferior Frontal Gyrus	R	38	32	2	123	0.46
Inferior Parietal Lobule	L	-38	-52	42	393	0.36
Insula	L	-38	16	8	141	0.35
Middle Frontal Gyrus	L	-38	38	22	38	0.35
Occipital Gyrus	L	-44	-66	-4	39	0.36
Superior Parietal Lobule	L	-26	-62	44	388	0.38
Supplementary Motor Area	L	-4	8	54	359	0.39
Underaged > Middle-aged						
Superior Parietal Lobule	R	24	-62	50	368	0.6
Young > Older						
Inferior Frontal Gyrus	R	44	20	24	20	0.35
Inferior Frontal Gyrus	R	46	8	28	138	0.32
Inferior Parietal Lobule	L	-38	-50	42	375	0.36
Insula	R	40	22	-2	1360	0.56
Insula	L	-38	16	10	182	0.36
Middle Frontal Gyrus	L	-38	38	22	38	0.35
Occipital Gyrus	L	-44	-66	-4	38	0.36
Superior Parietal Lobule	L	-26	-62	44	355	0.38
Supplementary Motor Area	R	-4	8	56	273	0.39
Underaged > Adults						
Angular Gyrus	R	24	-60	46	430	0.45

Maximum P is the maximum proportion of studies exhibiting the effect at the peak density weighted by sample size. The coordinates are Montreal Neurological Institute (MNI) standard stereotaxic spaces. The voxel size is 2 × 2 × 2mm^3. R/L: right/left hemisphere

Table S4 Brain activation differences among four age groups for tasks tapping response inhibition only

Regions	R/L	MNI			No.Voxs	Maximum P
		x	y	z		
Young > Underaged						
Angular Gyrus	R	42	-50	38	301	0.28
Inferior Parietal Lobule	L	-50	-42	38	429	0.26
Inferior Temporal Gyrus	R	44	-66	-4	119	0.21
Insula	R	34	18	0	572	0.29
Insula	L	-34	18	4	204	0.26
Middle Frontal Gyrus	R	36	38	28	1193	0.32
Occipital Gyrus	R	24	-64	44	150	0.23
Occipital Gyrus	L	-22	-74	40	20	0.19
Precentral Gyrus	R	42	4	46	653	0.28
Supplementary Motor Area	R	12	10	60	1173	0.33
SupraMarginal Gyrus	R	56	-42	32	744	0.28
Middle-aged > Young						
Inferior Parietal Lobule	R	50	-50	50	38	0.33
Middle-aged > Underaged						
Inferior Parietal Lobule	R	50	-48	42	713	0.49
Middle Frontal Gyrus	R	38	40	32	13	0.37
Supplementary Motor Area	R	4	16	52	80	0.44
Young > Older						
Angular Gyrus	R	38	-58	34	107	0.27
Inferior Frontal Gyrus	L	-44	20	4	365	0.31
Inferior Frontal Gyrus	L	-40	28	20	74	0.22
Inferior Frontal Gyrus	R	46	34	2	433	0.3
Inferior Frontal Gyrus	R	44	14	32	2923	0.4
Inferior Parietal Lobule	L	-32	-58	44	180	0.26
Middle Cingulate Cortex	L	-2	22	34	1014	0.33
Middle Cingulate Cortex	R	4	-24	34	408	0.23
Middle Temporal Gyrus	R	56	-32	0	1182	0.27
Occipital Gyrus	R	22	-70	36	115	0.25
Precentral Gyrus	L	-42	2	38	356	0.21
Superior Frontal Gyrus	R	20	10	60	598	0.3
Supplementary Motor Area	R	2	10	62	75	0.29
SupraMarginal Gyrus	L	-54	-42	36	744	0.31
Thalamus	R	4	-12	4	727	0.25
Thalamus	L	-10	-6	10	94	0.25

Maximum P is the maximum proportion of studies exhibiting the effect at the peak density weighted by sample size. The coordinates are Montreal Neurological Institute (MNI) standard stereotaxic spaces. The voxel size is $2 \times 2 \times 2mm^3$. R/L: right/left hemisphere

Table S5 Brain activation differences between cognitive inhibition and 54 contrasts for response inhibition

Regions	R/L	MNI			No.Voxs	Maximum P
		x	y	z		
Cognitive inhibition> Response inhibition						
Occipital Gyrus	L	-28	-68	34	266	0.21
Response inhibition > Cognitive inhibition						
Angular Gyrus	R	48	-48	36	322	0.21
Inferior Frontal Gyrus	L	-36	22	-12	452	0.21
Insula	L	-32	24	-8	434	0.21
Middle Cingulate Cortex	R	2	-34	32	289	0.19
Pallidum	R	16	4	0	491	0.2
Putamen	L	-20	10	2	330	0.21

Maximum P is the maximum proportion of studies exhibiting the effect at the peak density weighted by sample size. The coordinates are Montreal Neurological Institute (MNI) standard stereotaxic spaces. The voxel size is 2 × 2 × 2mm^3. R/L: right/left hemisphere

Table S6 Activation regions of leave-one-out analysis in cognitive inhibition tasks

Excluded Tasks	Insula	AG	IFG	SPL	SMA
Flanker	√	√	√	√	√
Simon	√	√	√	√	√
Stroop	√	×	√	×	√
WCST	√	√	√	√	√
Other	√	√	√	√	√

'√' indicates the region is included in the activation map; '×' indicates the region is not included in the activation map. AG, angular Gyrus; IFG, inferior frontal gyrus; SPL, superior parietal lobule; SMA, supplementary motor area. Results survived a cluster-level $p < .05$ FWE corrected for multiple comparisons and cluster-forming threshold $p < .001$ at voxel level.

Table S7 Activation regions of leave-one-out analysis in response inhibition tasks

Excluded Tasks	Insula	AG	MFG	IPL	MCC	MTG	OG
Antisaccade	√	√	√	√	√	√	√
Go/NoGo	√	√	√	√	√	√	√
SST	√	√	√	√	√	√	×

'√' indicates the region is included in the activation map; '×' indicates the region is not included in the activation map. AG, angular Gyrus; MFG, middle frontal gyrus; IPL, inferior parietal lobule; MCC, middle cingulate cortex; MTG, middle temporal gyrus; OG, occipital gyrus. Results survived a cluster-level $p < .05$ FWE corrected for multiple comparisons and cluster-forming threshold $p < .001$ at voxel level.

Table S8 Brain activation in successful inhibition for tasks tapping response inhibition only

Regions	R/L	MNI			No.Voxs	Maximum P
		x	y	z		
Fusiform	L	-34	-66	-10	177	0.29
Inferior Frontal Gyrus	R	46	34	16	1021	0.44
Inferior Frontal Gyrus	L	-40	24	-6	582	0.38
Inferior Parietal Lobule	L	-38	-52	44	308	0.33
Inferior Temporal Gyrus	R	48	-66	-6	183	0.29
Insula	R	40	20	-4	2084	0.51
Middle Cingulate Cortex	R	4	16	43	93	0.31
Middle Frontal Gyrus	R	46	18	20	1551	0.53
Middle Frontal Gyrus	R	32	42	28	863	0.41
Middle Frontal Gyrus	R	42	10	40	1175	0.46
Middle Temporal Gyrus	R	58	-20	-6	69	0.28
Middle Temporal Gyrus	R	62	-30	0	36	0.28
Pallidum	R	22	2	2	415	0.38
Pallidum	L	-12	4	4	354	0.34
Putamen	R	24	16	-4	495	0.48
Putamen	L	-28	12	0	1657	0.45
Superior Frontal Gyrus	R	24	4	58	50	0.28
Superior Temporal Gyrus	L	-54	-46	20	20	0.27
Supplementary Motor Area	R	4	14	52	1205	0.38
SupraMarginal Gyrus	R	48	-44	36	2168	0.47

Maximum P is the maximum proportion of studies exhibiting the effect at the peak density weighted by sample size. The coordinates are Montreal Neurological Institute (MNI) standard stereotaxic spaces. The voxel size is $2 \times 2 \times 2mm^3$. R/L: right/left hemisphere

Table S9 Brain activation differences among four age groups for tasks tapping successful response inhibition

Regions	R/L	MNI			No.Voxs	Maximum P
		x	y	z		
Young > Middle-aged						
Inferior Frontal Gyrus	R	44	20	24	557	0.45
Middle Cingulate Cortex	L	-4	20	38	130	0.33
Middle Temporal Gyrus	R	58	-26	-4	309	0.32
Middle Temporal Gyrus	L	-56	-48	18	139	0.31
Middle Frontal Gyrus	R	24	52	30	20	0.3
Pallidum	R	20	8	4	426	0.39
Precentral Gyrus	R	38	4	46	741	0.39
Putamen	L	-26	10	2	1251	0.48
Supplementary Motor Area	R	12	0	56	319	0.36
Middle-aged > Young						
Inferior Parietal Lobule	R	56	-52	50	47	0.5
Middle-aged > Underaged						
Angular Gyrus	R	58	-48	34	483	0.74
Middle-aged > Older						
Inferior Parietal Lobule	R	42	-46	50	174	0.59
Young > Underaged						
Angular Gyrus	R	46	-48	34	561	0.35
Inferior Parietal Lobule	L	-44	-50	42	28	0.33
Inferior Temporal Gyrus	R	48	-64	-4	301	0.35
Middle Temporal Gyrus	R	58	-28	-2	372	0.32
Middle Frontal Gyrus	R	36	40	28	489	0.39
Occipital Gyrus	R	38	-72	-4	95	0.32
Pallidum	L	-16	4	6	311	0.4
Precentral Gyrus	R	44	10	38	238	0.39
Precentral Gyrus	R	34	0	52	1005	0.43
Putamen	R	30	12	4	1188	0.49
Putamen	L	-26	10	4	641	0.43
Superior Temporal Gyrus	L	-54	-46	18	18	0.29
Supplementary Motor Area	R	6	10	52	1170	0.4
Adults > Underaged						
Angular Gyrus	R	28	-60	46	112	0.28
Inferior Parietal Lobule	R	40	-50	40	356	0.37
Inferior Parietal Lobule	L	-42	-52	40	73	0.31
Inferior Temporal Gyrus	R	48	-64	-4	343	0.31
Middle Frontal Gyrus	R	36	40	28	634	0.37
Middle Temporal Gyrus	R	58	-38	6	628	0.37
Occipital Gyrus	R	40	-74	-2	121	0.29
Precentral Gyrus	R	36	2	48	989	0.37
Precuneus	R	18	-70	44	18	0.3

Putamen	R	30	14	2	1249	0.48
Putamen	L	-24	8	4	758	0.38
Supplementary Motor Area	R	6	12	52	1192	0.37
SupraMarginal Gyrus	R	54	-46	30	794	0.37

Maximum P is the maximum proportion of studies exhibiting the effect at the peak density weighted by sample size. The coordinates are Montreal Neurological Institute (MNI) standard stereotaxic spaces. The voxel size is $2 \times 2 \times 2mm^3$. R/L: right/left hemisphere

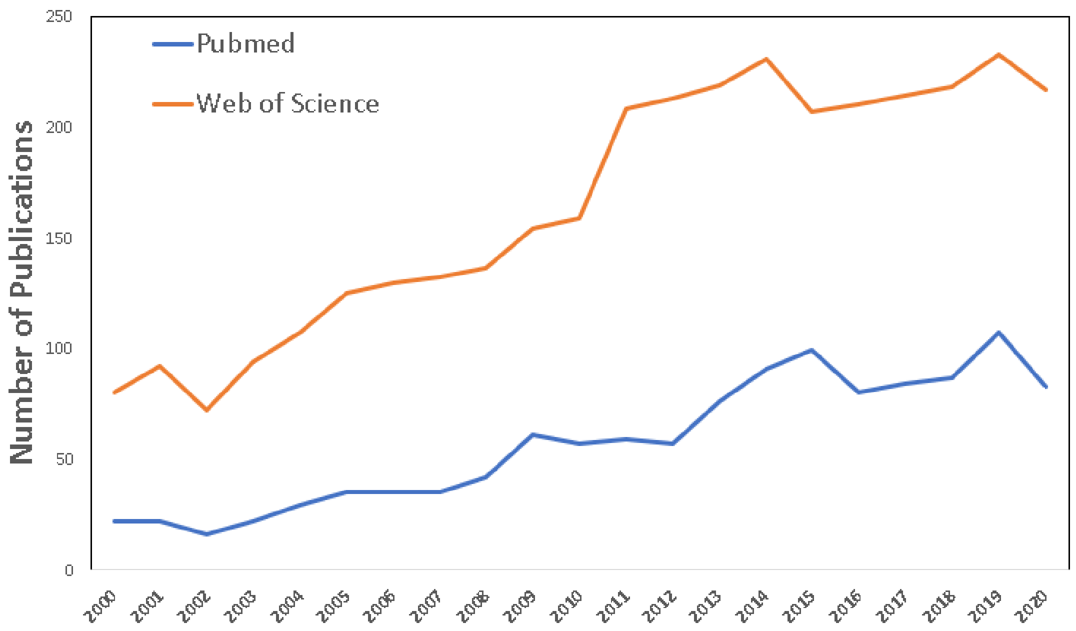

Figure S1 Number of studies investigating the development of inhibitory control across life span (1 Jan. 2000–31 Mar. 2020).

Two databases— PubMed and Web of Science— were searched using the keywords "inhibitory control" and "neural development".

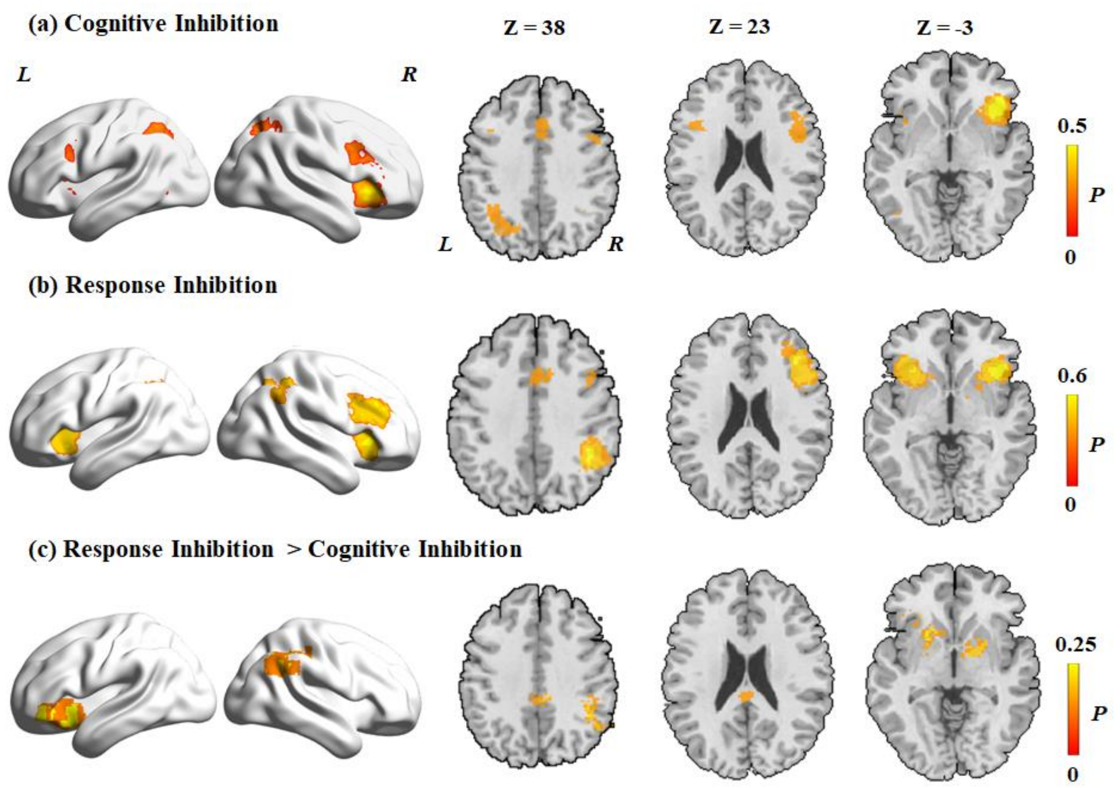

Figure S2 Brain activation differences between cognitive inhibition and randomly selected 54 contrasts for response inhibition.

Brain areas activated in (a) cognitive inhibition and (b) 54 contrasts for response inhibition. (c)Brain activation differences between cognitive inhibition and 54 contrasts for response inhibition. L/R: left/right. P represents the proportion of studies exhibiting the effect at the peak density weighted by sample size.

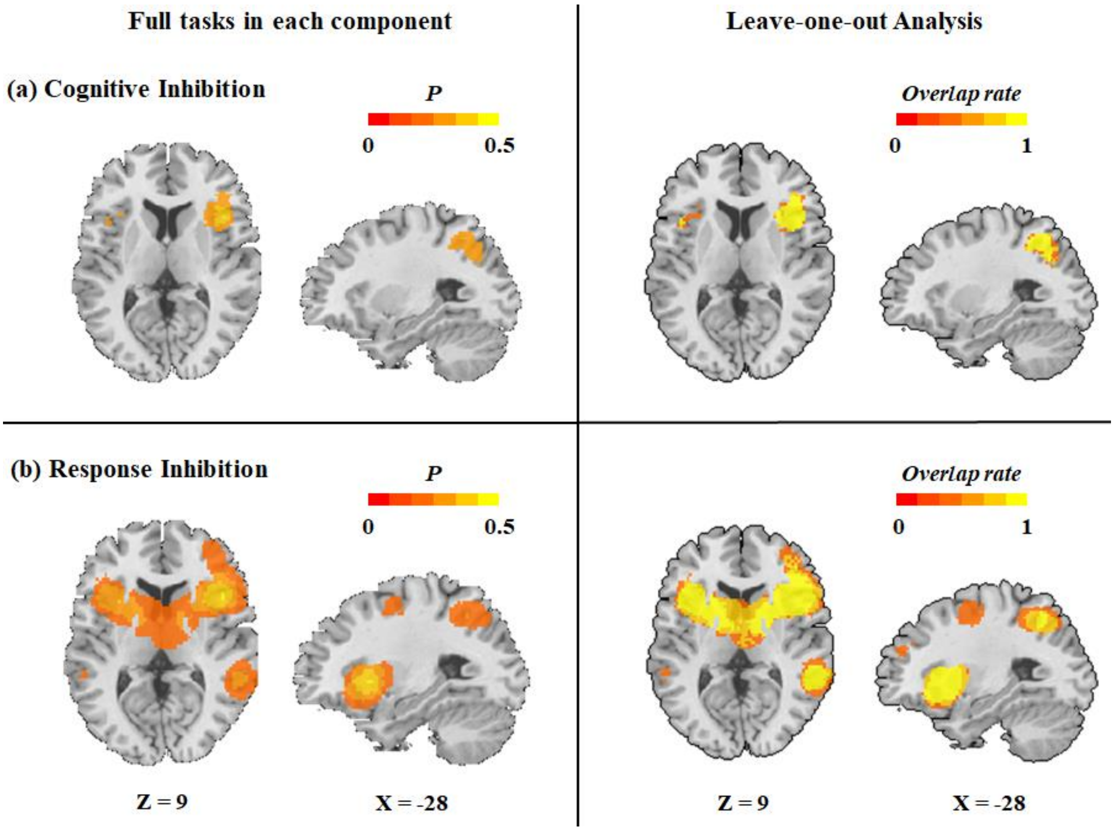

Figure S3 Activation maps between leave-one-out analysis and full tasks of each subcomponents in cognitive and response inhibition.

(a) Activation maps between leave-one-out analysis and full tasks from cognitive inhibition for MKDA analysis. (b) Activation maps between leave-one-out analysis and full tasks from response inhibition for MKDA analysis. *P* represents the proportion of studies exhibiting the effect at the peak density weighted by sample size

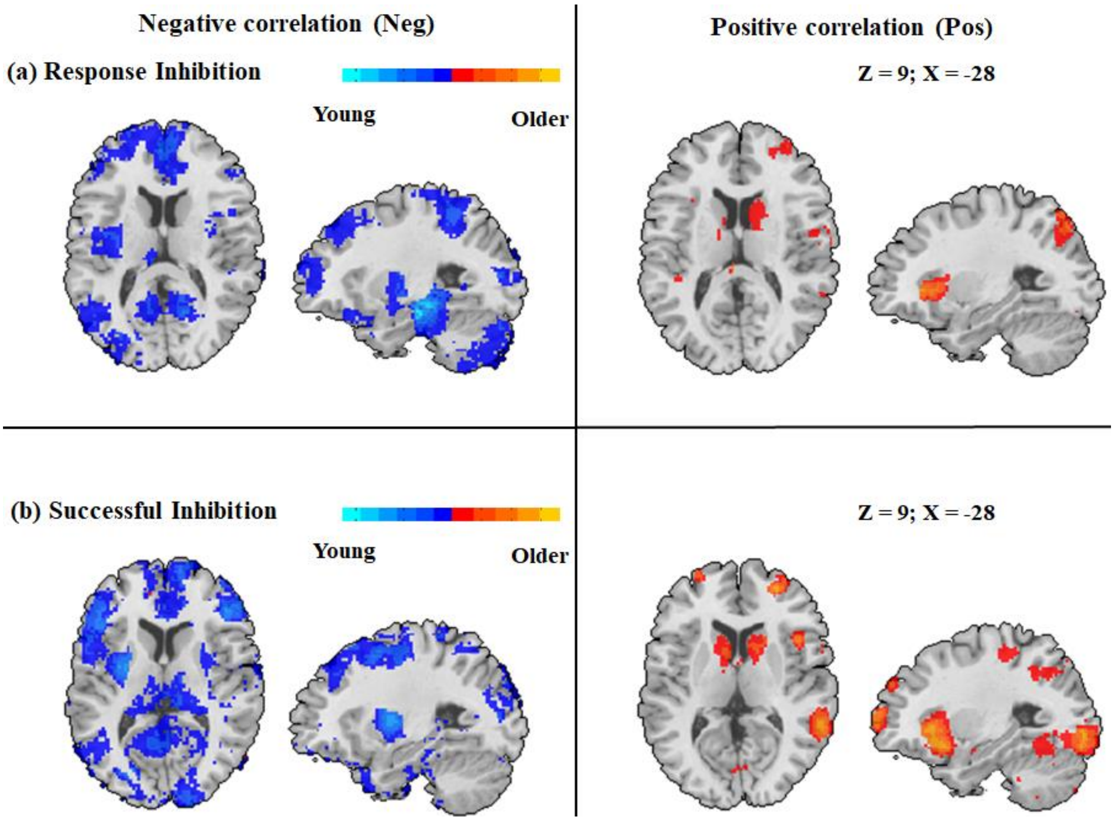

Figure S4 Activation maps displaying whole brain regression analysis in response inhibition and successful inhibition with age as a covariate.

(a) Correlation with age with clusters in response inhibition. (b) Correlation with age with clusters in successful inhibition.

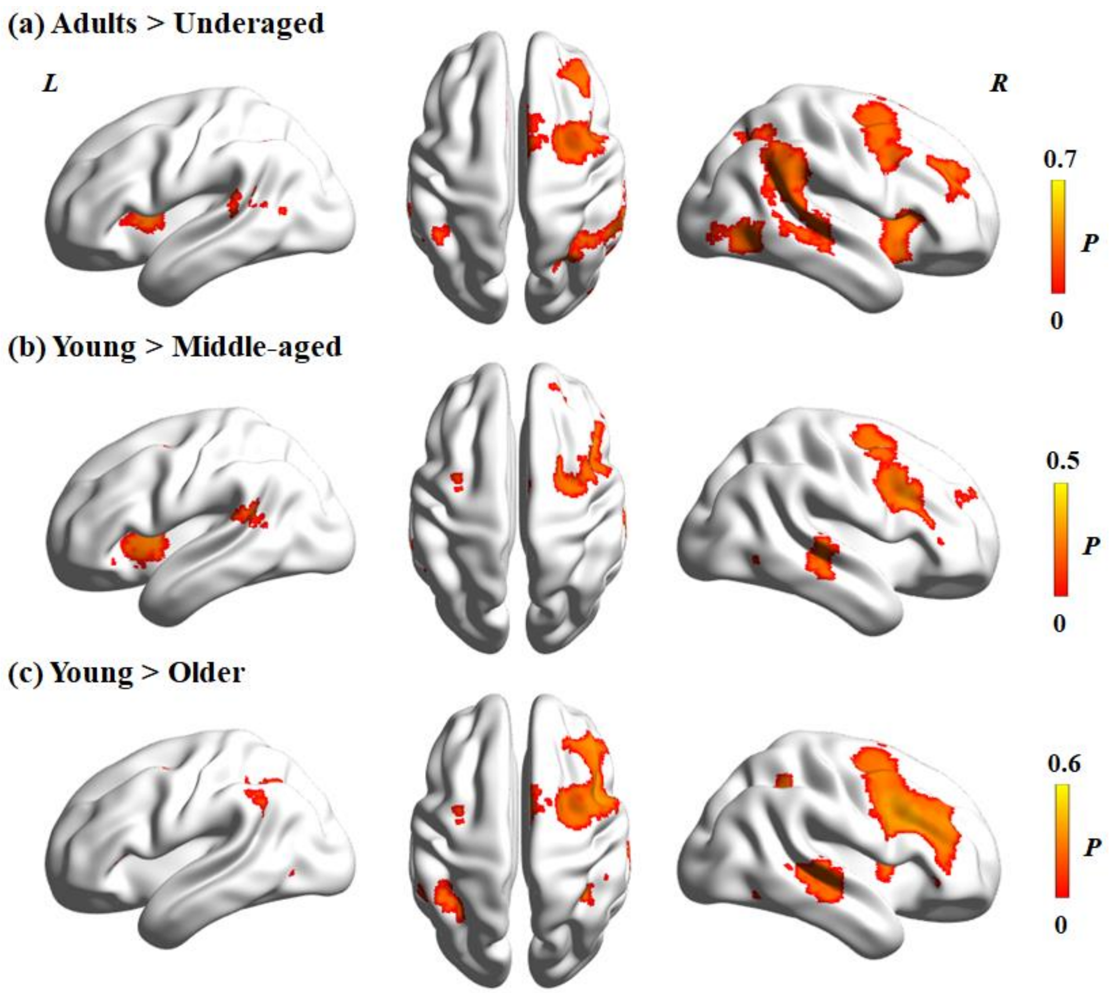

Figure S5 Brain activation differences among four age groups for tasks tapping successful response inhibition.

(a) Brain regions with higher activation in adults than underaged. (b) Brain regions with higher activation in young than middle-aged adults. (c) Brain regions with higher activation in young than older adults.

Reference

Agam, Y., Joseph, R. M., Barton, J. J., & Manoach, D. S. (2010). Reduced cognitive control of response inhibition by the anterior cingulate cortex in autism spectrum disorders. *Neuroimage, 52*(1), 336-347. doi:10.1016/j.neuroimage.2010.04.010

Altshuler, L. L., Bookheimer, S. Y., Townsend, J., Proenza, M. A., Eisenberger, N., Sabb, F., . . . Cohen, M. S. (2005). Blunted activation in orbitofrontal cortex during mania: a functional magnetic resonance imaging study. *Biol Psychiatry, 58*(10), 763-769. doi:10.1016/j.biopsych.2005.09.012

Anderson, B. A., Folk, C. L., & Courtney, S. M. (2016). Neural mechanisms of goal-contingent task disengagement: Response-irrelevant stimuli activate the default mode network. *Cortex, 81*, 221-230. doi:10.1016/j.cortex.2016.05.006

Andrews-Hanna, J. R., Mackiewicz Seghete, K. L., Claus, E. D., Burgess, G. C., Ruzic, L., & Banich, M. T. (2011). Cognitive control in adolescence: neural underpinnings and relation to self-report behaviors. *PLoS One, 6*(6), e21598. doi:10.1371/journal.pone.0021598

Aron, A. R., & Poldrack, R. A. (2006). Cortical and subcortical contributions to Stop signal response inhibition: role of the subthalamic nucleus. *J Neurosci, 26*(9), 2424-2433. doi:10.1523/jneurosci.4682-05.2006

Asahi, S., Okamoto, Y., Okada, G., Yamawaki, S., & Yokota, N. (2004). Negative correlation between right prefrontal activity during response inhibition and impulsiveness: a fMRI study. *Eur Arch Psychiatry Clin Neurosci, 254*(4), 245-251. doi:10.1007/s00406-004-0488-z

Baglio, F., Blasi, V., Falini, A., Farina, E., Mantovani, F., Olivotto, F., . . . Bozzali, M. (2011). Functional brain changes in early Parkinson's disease during motor response and motor inhibition. *Neurobiol Aging, 32*(1), 115-124. doi:10.1016/j.neurobiolaging.2008.12.009

Bannbers, E., Gingnell, M., Engman, J., Morell, A., Sylvén, S., Skalkidou, A., . . . Poromaa, I. S. (2013). Prefrontal activity during response inhibition decreases over time in the postpartum period. *Behav Brain Res, 241*, 132-138. doi:10.1016/j.bbr.2012.12.003

Barkataki, I., Kumari, V., Das, M., Sumich, A., Taylor, P., & Sharma, T. (2008). Neural correlates of deficient response inhibition in mentally disordered violent individuals. *Behav Sci Law, 26*(1), 51-64. doi:10.1002/bsl.787

Bartholdy, S., O'Daly, O. G., Campbell, I. C., Banaschewski, T., Barker, G., Bokde, A. L. W., . . . Schmidt, U. (2019). Neural Correlates of Failed Inhibitory Control as an Early Marker of Disordered Eating in Adolescents. *Biol Psychiatry, 85*(11), 956-965. doi:10.1016/j.biopsych.2019.01.027

Baumeister, S., Hohmann, S., Wolf, I., Plichta, M. M., Rechtsteiner, S., Zangl, M., . . . Brandeis, D. (2014). Sequential inhibitory control processes assessed through simultaneous EEG-fMRI. *Neuroimage, 94*, 349-359. doi:10.1016/j.neuroimage.2014.01.023

Behan, B., Stone, A., & Garavan, H. (2015). Right prefrontal and ventral striatum interactions underlying impulsive choice and impulsive responding. *Hum Brain Mapp, 36*(1), 187-198. doi:10.1002/hbm.22621

Bellgrove, M. A., Hester, R., & Garavan, H. (2004). The functional neuroanatomical correlates of response variability: evidence from a response inhibition task. *Neuropsychologia, 42*(14), 1910-1916. doi:10.1016/j.neuropsychologia.2004.05.007

Bennett, D. S., Mohamed, F. B., Carmody, D. P., Bendersky, M., Patel, S., Khorrami, M., . . . Lewis, M.

(2009). Response inhibition among early adolescents prenatally exposed to tobacco: an fMRI study. *Neurotoxicol Teratol, 31*(5), 283-290. doi:10.1016/j.ntt.2009.03.003

Berkman, E. T., Burklund, L., & Lieberman, M. D. (2009). Inhibitory spillover: intentional motor inhibition produces incidental limbic inhibition via right inferior frontal cortex. *Neuroimage, 47*(2), 705-712. doi:10.1016/j.neuroimage.2009.04.084

Berkman, E. T., Falk, E. B., & Lieberman, M. D. (2012). Interactive effects of three core goal pursuit processes on brain control systems: goal maintenance, performance monitoring, and response inhibition. *PLoS One, 7*(6), e40334. doi:10.1371/journal.pone.0040334

Berkman, E. T., Kahn, L. E., & Merchant, J. S. (2014). Training-induced changes in inhibitory control network activity. *J Neurosci, 34*(1), 149-157. doi:10.1523/jneurosci.3564-13.2014

Bernal, B., & Altman, N. (2009). Neural networks of motor and cognitive inhibition are dissociated between brain hemispheres: an fMRI study. *Int J Neurosci, 119*(10), 1848-1880. doi:10.1080/00207450802333029

Berron, D., Frühholz, S., & Herrmann, M. (2015). Neural control of enhanced filtering demands in a combined Flanker and Garner conflict task. *PLoS One, 10*(3), e0120582. doi:10.1371/journal.pone.0120582

Bhaijiwala, M., Chevrier, A., & Schachar, R. (2014). Withholding and canceling a response in ADHD adolescents. *Brain Behav, 4*(5), 602-614. doi:10.1002/brb3.244

Blasi, G., Goldberg, T. E., Weickert, T., Das, S., Kohn, P., Zoltick, B., . . . Mattay, V. S. (2006). Brain regions underlying response inhibition and interference monitoring and suppression. *Eur J Neurosci, 23*(6), 1658-1664. doi:10.1111/j.1460-9568.2006.04680.x

Bloemendaal, M., Froböse, M. I., Wegman, J., Zandbelt, B. B., van de Rest, O., Cools, R., & Aarts, E. (2018). Neuro-Cognitive Effects of Acute Tyrosine Administration on Reactive and Proactive Response Inhibition in Healthy Older Adults. *eNeuro, 5*(2). doi:10.1523/eneuro.0035-17.2018

Bobb, D. S., Jr., Adinoff, B., Laken, S. J., McClintock, S. M., Rubia, K., Huang, H. W., . . . Kozel, F. A. (2012). Neural correlates of successful response inhibition in unmedicated patients with late-life depression. *Am J Geriatr Psychiatry, 20*(12), 1057-1069. doi:10.1097/JGP.0b013e318235b728

Boecker, M., Drueke, B., Vorhold, V., Knops, A., Philippen, B., & Gauggel, S. (2011). When response inhibition is followed by response reengagement: an event-related fMRI study. *Hum Brain Mapp, 32*(1), 94-106. doi:10.1002/hbm.21001

Boehler, C. N., Appelbaum, L. G., Krebs, R. M., Hopf, J. M., & Woldorff, M. G. (2010). Pinning down response inhibition in the brain--conjunction analyses of the Stop-signal task. *Neuroimage, 52*(4), 1621-1632. doi:10.1016/j.neuroimage.2010.04.276

Boehler, C. N., Schevernels, H., Hopf, J. M., Stoppel, C. M., & Krebs, R. M. (2014). Reward prospect rapidly speeds up response inhibition via reactive control. *Cogn Affect Behav Neurosci, 14*(2), 593-609. doi:10.3758/s13415-014-0251-5

Booth, J. R., Burman, D. D., Meyer, J. R., Lei, Z., Trommer, B. L., Davenport, N. D., . . . Mesulam, M. M. (2003). Neural development of selective attention and response inhibition. *Neuroimage, 20*(2), 737-751. doi:10.1016/s1053-8119(03)00404-x

Borgwardt, S. J., Allen, P., Bhattacharyya, S., Fusar-Poli, P., Crippa, J. A., Seal, M. L., . . . McGuire, P. K. (2008). Neural basis of Delta-9-tetrahydrocannabinol and cannabidiol: effects during response inhibition. *Biol Psychiatry, 64*(11), 966-973. doi:10.1016/j.biopsych.2008.05.011

Braet, W., Johnson, K. A., Tobin, C. T., Acheson, R., Bellgrove, M. A., Robertson, I. H., & Garavan, H. (2009). Functional developmental changes underlying response inhibition and error-detection processes. *Neuropsychologia, 47*(14), 3143-3151. doi:10.1016/j.neuropsychologia.2009.07.018

Brass, M., Derrfuss, J., & von Cramon, D. Y. (2005). The inhibition of imitative and overlearned responses: a functional double dissociation. *Neuropsychologia, 43*(1), 89-98. doi:10.1016/j.neuropsychologia.2004.06.018

Brass, M., Zysset, S., & von Cramon, D. Y. (2001). The inhibition of imitative response tendencies. *Neuroimage, 14*(6), 1416-1423. doi:10.1006/nimg.2001.0944

Brevers, D., He, Q., Keller, B., Noël, X., & Bechara, A. (2017). Neural correlates of proactive and reactive motor response inhibition of gambling stimuli in frequent gamblers. *Sci Rep, 7*(1), 7394. doi:10.1038/s41598-017-07786-5

Brown, M. R., Goltz, H. C., Vilis, T., Ford, K. A., & Everling, S. (2006a). Inhibition and generation of saccades: rapid event-related fMRI of prosaccades, antisaccades, and nogo trials. *Neuroimage, 33*(2), 644-659. doi:10.1016/j.neuroimage.2006.07.002

Brown, M. R., Lebel, R. M., Dolcos, F., Wilman, A. H., Silverstone, P. H., Pazderka, H., . . . Dursun, S. M. (2012). Effects of emotional context on impulse control. *Neuroimage, 63*(1), 434-446. doi:10.1016/j.neuroimage.2012.06.056

Brown, S. M., Manuck, S. B., Flory, J. D., & Hariri, A. R. (2006b). Neural basis of individual differences in impulsivity: contributions of corticolimbic circuits for behavioral arousal and control. *Emotion, 6*(2), 239-245. doi:10.1037/1528-3542.6.2.239

Bruce, J., Fisher, P. A., Graham, A. M., Moore, W. E., Peake, S. J., & Mannering, A. M. (2013). Patterns of brain activation in foster children and nonmaltreated children during an inhibitory control task. *Dev Psychopathol, 25*(4 Pt 1), 931-941. doi:10.1017/s095457941300028x

Bunge, S. A., Dudukovic, N. M., Thomason, M. E., Vaidya, C. J., & Gabrieli, J. D. (2002). Immature frontal lobe contributions to cognitive control in children: evidence from fMRI. *Neuron, 33*(2), 301-311. doi:10.1016/s0896-6273(01)00583-9

Burke, M. R., & Barnes, G. R. (2011). The neural correlates of inhibiting pursuit to smoothly moving targets. *J Cogn Neurosci, 23*(11), 3294-3303. doi:10.1162/jocn_a_00025

Cai, W., Cannistraci, C. J., Gore, J. C., & Leung, H. C. (2014). Sensorimotor-independent prefrontal activity during response inhibition. *Hum Brain Mapp, 35*(5), 2119-2136. doi:10.1002/hbm.22315

Cai, W., & Leung, H. C. (2009). Cortical activity during manual response inhibition guided by color and orientation cues. *Brain Res, 1261*, 20-28. doi:10.1016/j.brainres.2008.12.073

Cai, W., & Leung, H. C. (2011). Rule-guided executive control of response inhibition: functional topography of the inferior frontal cortex. *PLoS One, 6*(6), e20840. doi:10.1371/journal.pone.0020840

Campanella, S., Absil, J., Carbia Sinde, C., Schroder, E., Peigneux, P., Bourguignon, M., . . . De Tiège, X. (2017). Neural correlates of correct and failed response inhibition in heavy versus light social drinkers: an fMRI study during a go/no-go task by healthy participants. *Brain Imaging Behav, 11*(6), 1796-1811. doi:10.1007/s11682-016-9654-y

Carrion, V. G., Garrett, A., Menon, V., Weems, C. F., & Reiss, A. L. (2008). Posttraumatic stress symptoms and brain function during a response-inhibition task: an fMRI study in youth.

Depress Anxiety, 25(6), 514-526. doi:10.1002/da.20346
Chen, C. Y., Huang, M. F., Yen, J. Y., Chen, C. S., Liu, G. C., Yen, C. F., & Ko, C. H. (2015). Brain correlates of response inhibition in Internet gaming disorder. *Psychiatry Clin Neurosci, 69*(4), 201-209. doi:10.1111/pcn.12224
Chevrier, A. D., Noseworthy, M. D., & Schachar, R. (2007). Dissociation of response inhibition and performance monitoring in the stop signal task using event-related fMRI. *Hum Brain Mapp, 28*(12), 1347-1358. doi:10.1002/hbm.20355
Chiang, H. S., Motes, M. A., Mudar, R. A., Rao, N. K., Mansinghani, S., Brier, M. R., . . . Hart, J., Jr. (2013). Semantic processing and response inhibition. *Neuroreport, 24*(16), 889-893. doi:10.1097/wnr.0000000000000014
Chikazoe, J., Jimura, K., Asari, T., Yamashita, K., Morimoto, H., Hirose, S., . . . Konishi, S. (2009a). Functional dissociation in right inferior frontal cortex during performance of go/no-go task. *Cereb Cortex, 19*(1), 146-152. doi:10.1093/cercor/bhn065
Chikazoe, J., Jimura, K., Hirose, S., Yamashita, K., Miyashita, Y., & Konishi, S. (2009b). Preparation to inhibit a response complements response inhibition during performance of a stop-signal task. *J Neurosci, 29*(50), 15870-15877. doi:10.1523/jneurosci.3645-09.2009
Chikazoe, J., Konishi, S., Asari, T., Jimura, K., & Miyashita, Y. (2007). Activation of right inferior frontal gyrus during response inhibition across response modalities. *J Cogn Neurosci, 19*(1), 69-80. doi:10.1162/jocn.2007.19.1.69
Chiu, Y. C., & Egner, T. (2015). Inhibition-Induced Forgetting Results from Resource Competition between Response Inhibition and Memory Encoding Processes. *J Neurosci, 35*(34), 11936-11945. doi:10.1523/jneurosci.0519-15.2015
Chuah, Y. M., Venkatraman, V., Dinges, D. F., & Chee, M. W. (2006). The neural basis of interindividual variability in inhibitory efficiency after sleep deprivation. *J Neurosci, 26*(27), 7156-7162. doi:10.1523/jneurosci.0906-06.2006
Cohen-Gilbert, J. E., Nickerson, L. D., Sneider, J. T., Oot, E. N., Seraikas, A. M., Rohan, M. L., & Silveri, M. M. (2017). College Binge Drinking Associated with Decreased Frontal Activation to Negative Emotional Distractors during Inhibitory Control. *Front Psychol, 8*, 1650. doi:10.3389/fpsyg.2017.01650
Congdon, E., Altshuler, L. L., Mumford, J. A., Karlsgodt, K. H., Sabb, F. W., Ventura, J., . . . Poldrack, R. A. (2014). Neural activation during response inhibition in adult attention-deficit/hyperactivity disorder: preliminary findings on the effects of medication and symptom severity. *Psychiatry Res, 222*(1-2), 17-28. doi:10.1016/j.pscychresns.2014.02.002
Cope, L. M., Hardee, J. E., Martz, M. E., Zucker, R. A., Nichols, T. E., & Heitzeg, M. M. (2020). Developmental maturation of inhibitory control circuitry in a high-risk sample: A longitudinal fMRI study. *Dev Cogn Neurosci, 43*, 100781. doi:10.1016/j.dcn.2020.100781
Costa, A., Riedel, M., Pogarell, O., Menzel-Zelnitschek, F., Schwarz, M., Reiser, M., . . . Ettinger, U. (2013). Methylphenidate effects on neural activity during response inhibition in healthy humans. *Cereb Cortex, 23*(5), 1179-1189. doi:10.1093/cercor/bhs107
Coxon, J. P., Goble, D. J., Leunissen, I., Van Impe, A., Wenderoth, N., & Swinnen, S. P. (2016). Functional Brain Activation Associated with Inhibitory Control Deficits in Older Adults. *Cereb Cortex, 26*(1), 12-22. doi:10.1093/cercor/bhu165
Crane, N. A., Jenkins, L. M., Dion, C., Meyers, K. K., Weldon, A. L., Gabriel, L. B., . . . Langenecker, S.

A. (2016). Comorbid anxiety increases cognitive control activation in Major Depressive Disorder. *Depress Anxiety, 33*(10), 967-977. doi:10.1002/da.22541

Criaud, M., Longcamp, M., Anton, J. L., Nazarian, B., Roth, M., Sescousse, G., . . . Boulinguez, P. (2017). Testing the physiological plausibility of conflicting psychological models of response inhibition: A forward inference fMRI study. *Behav Brain Res, 333*, 192-202. doi:10.1016/j.bbr.2017.06.030

Czapla, M., Baeuchl, C., Simon, J. J., Richter, B., Kluge, M., Friederich, H. C., . . . Loeber, S. (2017). Do alcohol-dependent patients show different neural activation during response inhibition than healthy controls in an alcohol-related fMRI go/no-go-task? *Psychopharmacology (Berl), 234*(6), 1001-1015. doi:10.1007/s00213-017-4541-9

Daly, E., Ecker, C., Hallahan, B., Deeley, Q., Craig, M., Murphy, C., . . . Murphy, D. G. (2014). Response inhibition and serotonin in autism: a functional MRI study using acute tryptophan depletion. *Brain, 137*(Pt 9), 2600-2610. doi:10.1093/brain/awu178

Dambacher, F., Sack, A. T., Lobbestael, J., Arntz, A., Brugman, S., & Schuhmann, T. (2014). A network approach to response inhibition: dissociating functional connectivity of neural components involved in action restraint and action cancellation. *Eur J Neurosci, 39*(5), 821-831. doi:10.1111/ejn.12425

de Zubicaray, G. I., Andrew, C., Zelaya, F. O., Williams, S. C., & Dumanoir, C. (2000). Motor response suppression and the prepotent tendency to respond: a parametric fMRI study. *Neuropsychologia, 38*(9), 1280-1291. doi:10.1016/s0028-3932(00)00033-6

Del-Ben, C. M., Deakin, J. F., McKie, S., Delvai, N. A., Williams, S. R., Elliott, R., . . . Anderson, I. M. (2005). The effect of citalopram pretreatment on neuronal responses to neuropsychological tasks in normal volunteers: an FMRI study. *Neuropsychopharmacology, 30*(9), 1724-1734. doi:10.1038/sj.npp.1300728

Doallo, S., Raymond, J. E., Shapiro, K. L., Kiss, M., Eimer, M., & Nobre, A. C. (2012). Response inhibition results in the emotional devaluation of faces: neural correlates as revealed by fMRI. *Soc Cogn Affect Neurosci, 7*(6), 649-659. doi:10.1093/scan/nsr031

Drueke, B., Schlaegel, S. M., Seifert, A., Moeller, O., Gründer, G., Gauggel, S., & Boecker, M. (2013). The role of 5-HT in response inhibition and re-engagement. *Eur Neuropsychopharmacol, 23*(8), 830-841. doi:10.1016/j.euroneuro.2013.05.005

Duerden, E. G., Taylor, M. J., Soorya, L. V., Wang, T., Fan, J., & Anagnostou, E. (2013). Neural correlates of inhibition of socially relevant stimuli in adults with autism spectrum disorder. *Brain Res, 1533*, 80-90. doi:10.1016/j.brainres.2013.08.021

Durston, S., Thomas, K. M., Worden, M. S., Yang, Y., & Casey, B. J. (2002). The effect of preceding context on inhibition: an event-related fMRI study. *Neuroimage, 16*(2), 449-453. doi:10.1006/nimg.2002.1074

Enriquez-Geppert, S., Konrad, C., Pantev, C., & Huster, R. J. (2010). Conflict and inhibition differentially affect the N200/P300 complex in a combined go/nogo and stop-signal task. *Neuroimage, 51*(2), 877-887. doi:10.1016/j.neuroimage.2010.02.043

Falconer, E., Bryant, R., Felmingham, K. L., Kemp, A. H., Gordon, E., Peduto, A., . . . Williams, L. M. (2008). The neural networks of inhibitory control in posttraumatic stress disorder. *J Psychiatry Neurosci, 33*(5), 413-422.

Fassbender, C., Hester, R., Murphy, K., Foxe, J. J., Foxe, D. M., & Garavan, H. (2009). Prefrontal and midline interactions mediating behavioural control. *Eur J Neurosci, 29*(1), 181-187.

doi:10.1111/j.1460-9568.2008.06557.x
Fassbender, C., Simoes-Franklin, C., Murphy, K., Hester, R., Meaney, J., Robertson, I. H., & Garavan, H. (2006). The Role of a Right Fronto-Parietal Network in Cognitive Control. *20*(4), 286-296. doi:10.1027/0269-8803.20.4.286
Feldstein Ewing, S. W., Houck, J. M., & Bryan, A. D. (2015). Neural activation during response inhibition is associated with adolescents' frequency of risky sex and substance use. *Addict Behav, 44*, 80-87. doi:10.1016/j.addbeh.2014.12.007
Fernandez-Ruiz, J., Peltsch, A., Alahyane, N., Brien, D. C., Coe, B. C., Garcia, A., & Munoz, D. P. (2018). Age related prefrontal compensatory mechanisms for inhibitory control in the antisaccade task. *Neuroimage, 165*, 92-101. doi:10.1016/j.neuroimage.2017.10.001
Fitzgerald, K. D., Zbrozek, C. D., Welsh, R. C., Britton, J. C., Liberzon, I., & Taylor, S. F. (2008). Pilot study of response inhibition and error processing in the posterior medial prefrontal cortex in healthy youth. *J Child Psychol Psychiatry, 49*(9), 986-994. doi:10.1111/j.1469-7610.2008.01906.x
Forstmann, B. U., Jahfari, S., Scholte, H. S., Wolfensteller, U., van den Wildenberg, W. P., & Ridderinkhof, K. R. (2008). Function and structure of the right inferior frontal cortex predict individual differences in response inhibition: a model-based approach. *J Neurosci, 28*(39), 9790-9796. doi:10.1523/jneurosci.1465-08.2008
Fu, L. P., Bi, G. H., Zou, Z. T., Wang, Y., Ye, E. M., Ma, L., . . . Yang, Z. (2008). Impaired response inhibition function in abstinent heroin dependents: an fMRI study. *Neurosci Lett, 438*(3), 322-326. doi:10.1016/j.neulet.2008.04.033
Galván, A., Poldrack, R. A., Baker, C. M., McGlennen, K. M., & London, E. D. (2011). Neural correlates of response inhibition and cigarette smoking in late adolescence. *Neuropsychopharmacology, 36*(5), 970-978. doi:10.1038/npp.2010.235
Garavan, H., Hester, R., Murphy, K., Fassbender, C., & Kelly, C. (2006). Individual differences in the functional neuroanatomy of inhibitory control. *Brain Res, 1105*(1), 130-142. doi:10.1016/j.brainres.2006.03.029
Garavan, H., Ross, T. J., Murphy, K., Roche, R. A., & Stein, E. A. (2002). Dissociable executive functions in the dynamic control of behavior: inhibition, error detection, and correction. *Neuroimage, 17*(4), 1820-1829. doi:10.1006/nimg.2002.1326
Garavan, H., Ross, T. J., & Stein, E. A. (1999). Right hemispheric dominance of inhibitory control: an event-related functional MRI study. *Proc Natl Acad Sci U S A, 96*(14), 8301-8306. doi:10.1073/pnas.96.14.8301
Gavazzi, G., Rossi, A., Orsolini, S., Diciotti, S., Giovannelli, F., Salvadori, E., . . . Viggiano, M. P. (2019). Impulsivity trait and proactive cognitive control: An fMRI study. *Eur J Neurosci, 49*(9), 1171-1179. doi:10.1111/ejn.14301
Goghari, V. M., & MacDonald, A. W., 3rd. (2009). The neural basis of cognitive control: response selection and inhibition. *Brain Cogn, 71*(2), 72-83. doi:10.1016/j.bandc.2009.04.004
Goldstein, M., Brendel, G., Tuescher, O., Pan, H., Epstein, J., Beutel, M., . . . Silbersweig, D. (2007). Neural substrates of the interaction of emotional stimulus processing and motor inhibitory control: an emotional linguistic go/no-go fMRI study. *Neuroimage, 36*(3), 1026-1040. doi:10.1016/j.neuroimage.2007.01.056
Goya-Maldonado, R., Walther, S., Simon, J., Stippich, C., Weisbrod, M., & Kaiser, S. (2010). Motor impulsivity and the ventrolateral prefrontal cortex. *Psychiatry Res, 183*(1), 89-91.

doi:10.1016/j.pscychresns.2010.04.006

Grandjean, J., D'Ostilio, K., Phillips, C., Balteau, E., Degueldre, C., Luxen, A., . . . Collette, F. (2012). Modulation of brain activity during a Stroop inhibitory task by the kind of cognitive control required. *PLoS One, 7*(7), e41513. doi:10.1371/journal.pone.0041513

Habermeyer, B., Esposito, F., Händel, N., Lemoine, P., Kuhl, H. C., Klarhöfer, M., . . . Graf, M. (2013). Response inhibition in pedophilia: an FMRI pilot study. *Neuropsychobiology, 68*(4), 228-237. doi:10.1159/000355295

Hakvoort Schwerdtfeger, R. M., Alahyane, N., Brien, D. C., Coe, B. C., Stroman, P. W., & Munoz, D. P. (2012). Preparatory neural networks are impaired in adults with attention-deficit/hyperactivity disorder during the antisaccade task. *Neuroimage Clin, 2*, 63-78. doi:10.1016/j.nicl.2012.10.006

Halari, R., Simic, M., Pariante, C. M., Papadopoulos, A., Cleare, A., Brammer, M., . . . Rubia, K. (2009). Reduced activation in lateral prefrontal cortex and anterior cingulate during attention and cognitive control functions in medication-naïve adolescents with depression compared to controls. *J Child Psychol Psychiatry, 50*(3), 307-316. doi:10.1111/j.1469-7610.2008.01972.x

Hansen, N. S., Thayer, R. E., Feldstein Ewing, S. W., Sabbineni, A., & Bryan, A. D. (2018). Neural Correlates of Risky Sex and Response Inhibition in High-Risk Adolescents. *J Res Adolesc, 28*(1), 56-69. doi:10.1111/jora.12344

Hare, T. A., Tottenham, N., Davidson, M. C., Glover, G. H., & Casey, B. J. (2005). Contributions of amygdala and striatal activity in emotion regulation. *Biol Psychiatry, 57*(6), 624-632. doi:10.1016/j.biopsych.2004.12.038

Hendrick, O. M., Ide, J. S., Luo, X., & Li, C. S. (2010). Dissociable processes of cognitive control during error and non-error conflicts: a study of the stop signal task. *PLoS One, 5*(10), e13155. doi:10.1371/journal.pone.0013155

Hester, R., & Garavan, H. (2004). Executive dysfunction in cocaine addiction: evidence for discordant frontal, cingulate, and cerebellar activity. *J Neurosci, 24*(49), 11017-11022. doi:10.1523/jneurosci.3321-04.2004

Hester, R., Madeley, J., Murphy, K., & Mattingley, J. B. (2009). Learning from errors: error-related neural activity predicts improvements in future inhibitory control performance. *J Neurosci, 29*(22), 7158-7165. doi:10.1523/jneurosci.4337-08.2009

Hester, R. L., Murphy, K., Foxe, J. J., Foxe, D. M., Javitt, D. C., & Garavan, H. (2004). Predicting success: patterns of cortical activation and deactivation prior to response inhibition. *J Cogn Neurosci, 16*(5), 776-785. doi:10.1162/089892904970726

Horn, N. R., Dolan, M., Elliott, R., Deakin, J. F., & Woodruff, P. W. (2003). Response inhibition and impulsivity: an fMRI study. *Neuropsychologia, 41*(14), 1959-1966. doi:10.1016/s0028-3932(03)00077-0

Hu, S., Ide, J. S., Chao, H. H., Castagna, B., Fischer, K. A., Zhang, S., & Li, C. R. (2018). Structural and functional cerebral bases of diminished inhibitory control during healthy aging. *Hum Brain Mapp, 39*(12), 5085-5096. doi:10.1002/hbm.24347

Huang, S., Zhu, Z., Zhang, W., Chen, Y., & Zhen, S. (2017). Trait impulsivity components correlate differently with proactive and reactive control. *PLoS One, 12*(4), e0176102. doi:10.1371/journal.pone.0176102

Hughes, M. E., Budd, T. W., Fulham, W. R., Lancaster, S., Woods, W., Rossell, S. L., & Michie, P. T.

(2014). Sustained brain activation supporting stop-signal task performance. *Eur J Neurosci, 39*(8), 1363-1369. doi:10.1111/ejn.12497

Hughes, M. E., Fulham, W. R., Johnston, P. J., & Michie, P. T. (2012). Stop-signal response inhibition in schizophrenia: behavioural, event-related potential and functional neuroimaging data. *Biol Psychol, 89*(1), 220-231. doi:10.1016/j.biopsycho.2011.10.013

Hughes, M. E., Johnston, P. J., Fulham, W. R., Budd, T. W., & Michie, P. T. (2013). Stop-signal task difficulty and the right inferior frontal gyrus. *Behav Brain Res, 256*, 205-213. doi:10.1016/j.bbr.2013.08.026

Huster, R. J., Westerhausen, R., Pantev, C., & Konrad, C. (2010). The role of the cingulate cortex as neural generator of the N200 and P300 in a tactile response inhibition task. *Hum Brain Mapp, 31*(8), 1260-1271. doi:10.1002/hbm.20933

Iannaccone, R., Hauser, T. U., Staempfli, P., Walitza, S., Brandeis, D., & Brem, S. (2015). Conflict monitoring and error processing: new insights from simultaneous EEG-fMRI. *Neuroimage, 105*, 395-407. doi:10.1016/j.neuroimage.2014.10.028

Jaffard, M., Longcamp, M., Velay, J. L., Anton, J. L., Roth, M., Nazarian, B., & Boulinguez, P. (2008). Proactive inhibitory control of movement assessed by event-related fMRI. *Neuroimage, 42*(3), 1196-1206. doi:10.1016/j.neuroimage.2008.05.041

Jahfari, S., Verbruggen, F., Frank, M. J., Waldorp, L. J., Colzato, L., Ridderinkhof, K. R., & Forstmann, B. U. (2012). How preparation changes the need for top-down control of the basal ganglia when inhibiting premature actions. *J Neurosci, 32*(32), 10870-10878. doi:10.1523/jneurosci.0902-12.2012

Jahfari, S., Waldorp, L., van den Wildenberg, W. P., Scholte, H. S., Ridderinkhof, K. R., & Forstmann, B. U. (2011). Effective connectivity reveals important roles for both the hyperdirect (fronto-subthalamic) and the indirect (fronto-striatal-pallidal) fronto-basal ganglia pathways during response inhibition. *J Neurosci, 31*(18), 6891-6899. doi:10.1523/jneurosci.5253-10.2011

Jamadar, S., Hughes, M., Fulham, W. R., Michie, P. T., & Karayanidis, F. (2010). The spatial and temporal dynamics of anticipatory preparation and response inhibition in task-switching. *Neuroimage, 51*(1), 432-449. doi:10.1016/j.neuroimage.2010.01.090

Janssen, T. W., Heslenfeld, D. J., van Mourik, R., Logan, G. D., & Oosterlaan, J. (2015). Neural correlates of response inhibition in children with attention-deficit/hyperactivity disorder: A controlled version of the stop-signal task. *Psychiatry Res, 233*(2), 278-284. doi:10.1016/j.pscychresns.2015.07.007

Jiang, J., Bailey, K., Xiang, L., Zhang, L., & Zhang, Q. (2016). Comparing the Neural Correlates of Conscious and Unconscious Conflict Control in a Masked Stroop Priming Task. *Front Hum Neurosci, 10*, 297. doi:10.3389/fnhum.2016.00297

Jimura, K., Hirose, S., Kunimatsu, A., Ohtomo, K., Koike, Y., & Konishi, S. (2014). Late enhancement of brain-behavior correlations during response inhibition. *Neuroscience, 274*, 383-392. doi:10.1016/j.neuroscience.2014.05.058

Kaladjian, A., Jeanningros, R., Azorin, J. M., Grimault, S., Anton, J. L., & Mazzola-Pomietto, P. (2007). Blunted activation in right ventrolateral prefrontal cortex during motor response inhibition in schizophrenia. *Schizophr Res, 97*(1-3), 184-193. doi:10.1016/j.schres.2007.07.033

Kaladjian, A., Jeanningros, R., Azorin, J. M., Nazarian, B., Roth, M., Anton, J. L., &

Mazzola-Pomietto, P. (2009a). Remission from mania is associated with a decrease in amygdala activation during motor response inhibition. *Bipolar Disord, 11*(5), 530-538. doi:10.1111/j.1399-5618.2009.00722.x

Kaladjian, A., Jeanningros, R., Azorin, J. M., Nazarian, B., Roth, M., & Mazzola-Pomietto, P. (2009b). Reduced brain activation in euthymic bipolar patients during response inhibition: an event-related fMRI study. *Psychiatry Res, 173*(1), 45-51. doi:10.1016/j.pscychresns.2008.08.003

Karch, S., Jäger, L., Karamatskos, E., Graz, C., Stammel, A., Flatz, W., . . . Mulert, C. (2008). Influence of trait anxiety on inhibitory control in alcohol-dependent patients: simultaneous acquisition of ERPs and BOLD responses. *J Psychiatr Res, 42*(9), 734-745. doi:10.1016/j.jpsychires.2007.07.016

Kärgel, C., Massau, C., Weiß, S., Walter, M., Borchardt, V., Krueger, T. H., . . . Schiffer, B. (2017). Evidence for superior neurobiological and behavioral inhibitory control abilities in non-offending as compared to offending pedophiles. *Hum Brain Mapp, 38*(2), 1092-1104. doi:10.1002/hbm.23443

Karoly, H. C., Weiland, B. J., Sabbineni, A., & Hutchison, K. E. (2014). Preliminary functional MRI results from a combined stop-signal alcohol-cue task. *J Stud Alcohol Drugs, 75*(4), 664-673. doi:10.15288/jsad.2014.75.664

Kelly, A. M., Hester, R., Murphy, K., Javitt, D. C., Foxe, J. J., & Garavan, H. (2004). Prefrontal-subcortical dissociations underlying inhibitory control revealed by event-related fMRI. *Eur J Neurosci, 19*(11), 3105-3112. doi:10.1111/j.0953-816X.2004.03429.x

Kim, C., Kroger, J. K., & Kim, J. (2011). A functional dissociation of conflict processing within anterior cingulate cortex. *Hum Brain Mapp, 32*(2), 304-312. doi:10.1002/hbm.21020

King, J. A., Korb, F. M., & Egner, T. (2012). Priming of control: implicit contextual cuing of top-down attentional set. *J Neurosci, 32*(24), 8192-8200. doi:10.1523/jneurosci.0934-12.2012

Ko, C. H., Hsieh, T. J., Chen, C. Y., Yen, C. F., Chen, C. S., Yen, J. Y., . . . Liu, G. C. (2014). Altered brain activation during response inhibition and error processing in subjects with Internet gaming disorder: a functional magnetic imaging study. *Eur Arch Psychiatry Clin Neurosci, 264*(8), 661-672. doi:10.1007/s00406-013-0483-3

Kolodny, T., Mevorach, C., & Shalev, L. (2017). Isolating response inhibition in the brain: Parietal versus frontal contribution. *Cortex, 88*, 173-185. doi:10.1016/j.cortex.2016.12.012

Konishi, S., Nakajima, K., Uchida, I., Kikyo, H., Kameyama, M., & Miyashita, Y. (1999). Common inhibitory mechanism in human inferior prefrontal cortex revealed by event-related functional MRI. *Brain, 122 (Pt 5)*, 981-991. doi:10.1093/brain/122.5.981

Konishi, S., Watanabe, T., Jimura, K., Chikazoe, J., Hirose, S., Kimura, H. M., & Miyashita, Y. (2011). Role for presupplementary motor area in inhibition of cognitive set interference. *J Cogn Neurosci, 23*(3), 737-745. doi:10.1162/jocn.2010.21480

Korsch, M., Frühholz, S., & Herrmann, M. (2014). Ageing differentially affects neural processing of different conflict types-an fMRI study. *Front Aging Neurosci, 6*, 57. doi:10.3389/fnagi.2014.00057

Langenecker, S. A., Kennedy, S. E., Guidotti, L. M., Briceno, E. M., Own, L. S., Hooven, T., . . . Zubieta, J. K. (2007). Frontal and limbic activation during inhibitory control predicts treatment

response in major depressive disorder. *Biol Psychiatry, 62*(11), 1272-1280. doi:10.1016/j.biopsych.2007.02.019
Langenecker, S. A., & Nielson, K. A. (2003). Frontal recruitment during response inhibition in older adults replicated with fMRI. *Neuroimage, 20*(2), 1384-1392. doi:10.1016/s1053-8119(03)00372-0
Laurens, K. R., Kiehl, K. A., & Liddle, P. F. (2005). A supramodal limbic-paralimbic-neocortical network supports goal-directed stimulus processing. *Hum Brain Mapp, 24*(1), 35-49. doi:10.1002/hbm.20062
Lavallee, C. F., Herrmann, C. S., Weerda, R., & Huster, R. J. (2014). Stimulus-response mappings shape inhibition processes: a combined EEG-fMRI study of contextual stopping. *PLoS One, 9*(4), e96159. doi:10.1371/journal.pone.0096159
Lawrence, E. J., Rubia, K., Murray, R. M., McGuire, P. K., Walshe, M., Allin, M., . . . Nosarti, C. (2009). The neural basis of response inhibition and attention allocation as mediated by gestational age. *Hum Brain Mapp, 30*(3), 1038-1050. doi:10.1002/hbm.20564
Le, T. M., Chao, H., Levy, I., & Li, C. R. (2020). Age-Related Changes in the Neural Processes of Reward-Directed Action and Inhibition of Action. *Front Psychol, 11*, 1121. doi:10.3389/fpsyg.2020.01121
Lei, D., Ma, J., Du, X., Shen, G., Tian, M., & Li, G. (2012). Altered brain activation during response inhibition in children with primary nocturnal enuresis: an fMRI study. *Hum Brain Mapp, 33*(12), 2913-2919. doi:10.1002/hbm.21411
Lemire-Rodger, S., Lam, J., Viviano, J. D., Stevens, W. D., Spreng, R. N., & Turner, G. R. (2019). Inhibit, switch, and update: A within-subject fMRI investigation of executive control. *Neuropsychologia, 132*, 107134. doi:10.1016/j.neuropsychologia.2019.107134
Lenartowicz, A., Verbruggen, F., Logan, G. D., & Poldrack, R. A. (2011). Inhibition-related activation in the right inferior frontal gyrus in the absence of inhibitory cues. *J Cogn Neurosci, 23*(11), 3388-3399. doi:10.1162/jocn_a_00031
Leung, H. C., & Cai, W. (2007). Common and differential ventrolateral prefrontal activity during inhibition of hand and eye movements. *J Neurosci, 27*(37), 9893-9900. doi:10.1523/jneurosci.2837-07.2007
Li, C. S., Huang, C., Constable, R. T., & Sinha, R. (2006). Imaging response inhibition in a stop-signal task: neural correlates independent of signal monitoring and post-response processing. *J Neurosci, 26*(1), 186-192. doi:10.1523/jneurosci.3741-05.2006
Li, C. S., Yan, P., Chao, H. H., Sinha, R., Paliwal, P., Constable, R. T., . . . Lee, T. W. (2008). Error-specific medial cortical and subcortical activity during the stop signal task: a functional magnetic resonance imaging study. *Neuroscience, 155*(4), 1142-1151. doi:10.1016/j.neuroscience.2008.06.062
Liddle, P. F., Kiehl, K. A., & Smith, A. M. (2001). Event-related fMRI study of response inhibition. *Hum Brain Mapp, 12*(2), 100-109. doi:10.1002/1097-0193(200102)12:2<100::aid-hbm1007>3.0.co;2-6
Liu, J., Zubieta, J. K., & Heitzeg, M. (2012). Sex differences in anterior cingulate cortex activation during impulse inhibition and behavioral correlates. *Psychiatry Res, 201*(1), 54-62. doi:10.1016/j.pscychresns.2011.05.008
Liu, X., Banich, M. T., Jacobson, B. L., & Tanabe, J. L. (2004). Common and distinct neural substrates of attentional control in an integrated Simon and spatial Stroop task as

assessed by event-related fMRI. *Neuroimage, 22*(3), 1097-1106. doi:10.1016/j.neuroimage.2004.02.033

Liu, Y., Angstadt, M., Taylor, S. F., & Fitzgerald, K. D. (2016). The typical development of posterior medial frontal cortex function and connectivity during task control demands in youth 8-19years old. *Neuroimage, 137*, 97-106. doi:10.1016/j.neuroimage.2016.05.019

Lock, J., Garrett, A., Beenhakker, J., & Reiss, A. L. (2011). Aberrant brain activation during a response inhibition task in adolescent eating disorder subtypes. *Am J Psychiatry, 168*(1), 55-64. doi:10.1176/appi.ajp.2010.10010056

Longo, C. A., Fried, P. A., Cameron, I., & Smith, A. M. (2013). The long-term effects of prenatal nicotine exposure on response inhibition: an fMRI study of young adults. *Neurotoxicol Teratol, 39*, 9-18. doi:10.1016/j.ntt.2013.05.007

Maguire, R. P., Broerse, A., de Jong, B. M., Cornelissen, F. W., Meiners, L. C., Leenders, K. L., & den Boer, J. A. (2003). Evidence of enhancement of spatial attention during inhibition of a visuo-motor response. *Neuroimage, 20*(2), 1339-1345. doi:10.1016/s1053-8119(03)00402-6

Majid, D. S., Cai, W., Corey-Bloom, J., & Aron, A. R. (2013). Proactive selective response suppression is implemented via the basal ganglia. *J Neurosci, 33*(33), 13259-13269. doi:10.1523/jneurosci.5651-12.2013

Manza, P., Schwartz, G., Masson, M., Kann, S., Volkow, N. D., Li, C. R., & Leung, H. C. (2018). Levodopa improves response inhibition and enhances striatal activation in early-stage Parkinson's disease. *Neurobiol Aging, 66*, 12-22. doi:10.1016/j.neurobiolaging.2018.02.003

Marco-Pallarés, J., Camara, E., Münte, T. F., & Rodríguez-Fornells, A. (2008). Neural mechanisms underlying adaptive actions after slips. *J Cogn Neurosci, 20*(9), 1595-1610. doi:10.1162/jocn.2008.20117

Marsh, R., Zhu, H., Schultz, R. T., Quackenbush, G., Royal, J., Skudlarski, P., & Peterson, B. S. (2006). A developmental fMRI study of self-regulatory control. *Hum Brain Mapp, 27*(11), 848-863. doi:10.1002/hbm.20225

Matsuda, T., Matsuura, M., Ohkubo, T., Ohkubo, H., Matsushima, E., Inoue, K., . . . Kojima, T. (2004). Functional MRI mapping of brain activation during visually guided saccades and antisaccades: cortical and subcortical networks. *Psychiatry Res, 131*(2), 147-155. doi:10.1016/j.pscychresns.2003.12.007

Mayer, A. R., Teshiba, T. M., Franco, A. R., Ling, J., Shane, M. S., Stephen, J. M., & Jung, R. E. (2012). Modeling conflict and error in the medial frontal cortex. *Hum Brain Mapp, 33*(12), 2843-2855. doi:10.1002/hbm.21405

Mazzola-Pomietto, P., Kaladjian, A., Azorin, J. M., Anton, J. L., & Jeanningros, R. (2009). Bilateral decrease in ventrolateral prefrontal cortex activation during motor response inhibition in mania. *J Psychiatr Res, 43*(4), 432-441. doi:10.1016/j.jpsychires.2008.05.004

McNab, F., Leroux, G., Strand, F., Thorell, L., Bergman, S., & Klingberg, T. (2008). Common and unique components of inhibition and working memory: an fMRI, within-subjects investigation. *Neuropsychologia, 46*(11), 2668-2682. doi:10.1016/j.neuropsychologia.2008.04.023

Mehren, A., Özyurt, J., Thiel, C. M., Brandes, M., Lam, A. P., & Philipsen, A. (2019). Effects of Acute Aerobic Exercise on Response Inhibition in Adult Patients with ADHD. *Sci Rep, 9*(1),

19884. doi:10.1038/s41598-019-56332-y
Mitchell, R. L. (2005). The BOLD response during Stroop task-like inhibition paradigms: Effects of task difficulty and task-relevant modality. *Brain Cogn, 59*(1), 23-37. doi:10.1016/j.bandc.2005.04.001
Mobbs, D., Eckert, M. A., Mills, D., Korenberg, J., Bellugi, U., Galaburda, A. M., & Reiss, A. L. (2007). Frontostriatal dysfunction during response inhibition in Williams syndrome. *Biol Psychiatry, 62*(3), 256-261. doi:10.1016/j.biopsych.2006.05.041
Mulligan, R. C., Knopik, V. S., Sweet, L. H., Fischer, M., Seidenberg, M., & Rao, S. M. (2011). Neural correlates of inhibitory control in adult attention deficit/hyperactivity disorder: evidence from the Milwaukee longitudinal sample. *Psychiatry Res, 194*(2), 119-129. doi:10.1016/j.pscychresns.2011.02.003
Nakata, H., Sakamoto, K., Ferretti, A., Gianni Perrucci, M., Del Gratta, C., Kakigi, R., & Romani, G. L. (2008). Executive functions with different motor outputs in somatosensory Go/Nogo tasks: an event-related functional MRI study. *Brain Res Bull, 77*(4), 197-205. doi:10.1016/j.brainresbull.2008.07.008
Ness, V., & Beste, C. (2013). The role of the striatum in goal activation of cascaded actions. *Neuropsychologia, 51*(13), 2562-2571. doi:10.1016/j.neuropsychologia.2013.09.032
Nielson, K. A., Langenecker, S. A., & Garavan, H. (2002). Differences in the functional neuroanatomy of inhibitory control across the adult life span. *Psychol Aging, 17*(1), 56-71. doi:10.1037//0882-7974.17.1.56
Nielson, K. A., Langenecker, S. A., Ross, T. J., Garavan, H., Rao, S. M., & Stein, E. A. (2004). Comparability of functional MRI response in young and old during inhibition. *Neuroreport, 15*(1), 129-133. doi:10.1097/00001756-200401190-00025
O'Connor, D. A., Rossiter, S., Yücel, M., Lubman, D. I., & Hester, R. (2012). Successful inhibitory control over an immediate reward is associated with attentional disengagement in visual processing areas. *Neuroimage, 62*(3), 1841-1847. doi:10.1016/j.neuroimage.2012.05.040
Ordaz, S. J., Foran, W., Velanova, K., & Luna, B. (2013). Longitudinal growth curves of brain function underlying inhibitory control through adolescence. *J Neurosci, 33*(46), 18109-18124. doi:10.1523/jneurosci.1741-13.2013
Padmala, S., & Pessoa, L. (2010). Moment-to-moment fluctuations in fMRI amplitude and interregion coupling are predictive of inhibitory performance. *Cogn Affect Behav Neurosci, 10*(2), 279-297. doi:10.3758/cabn.10.2.279
Padmanabhan, A., Geier, C. F., Ordaz, S. J., Teslovich, T., & Luna, B. (2011). Developmental changes in brain function underlying the influence of reward processing on inhibitory control. *Dev Cogn Neurosci, 1*(4), 517-529. doi:10.1016/j.dcn.2011.06.004
Page, L. A., Rubia, K., Deeley, Q., Daly, E., Toal, F., Mataix-Cols, D., . . . Murphy, D. G. (2009). A functional magnetic resonance imaging study of inhibitory control in obsessive-compulsive disorder. *Psychiatry Res, 174*(3), 202-209. doi:10.1016/j.pscychresns.2009.05.002
Passarotti, A. M., Sweeney, J. A., & Pavuluri, M. N. (2010). Neural correlates of response inhibition in pediatric bipolar disorder and attention deficit hyperactivity disorder. *Psychiatry Res, 181*(1), 36-43. doi:10.1016/j.pscychresns.2009.07.002
Poirel, N., Borst, G., Simon, G., Rossi, S., Cassotti, M., Pineau, A., & Houdé, O. (2012). Number conservation is related to children's prefrontal inhibitory control: an fMRI study of a

piagetian task. *PLoS One, 7*(7), e40802. doi:10.1371/journal.pone.0040802
Pornpattananangkul, N., Hariri, A. R., Harada, T., Mano, Y., Komeda, H., Parrish, T. B., . . . Chiao, J. Y. (2016). Cultural influences on neural basis of inhibitory control. *Neuroimage, 139*, 114-126. doi:10.1016/j.neuroimage.2016.05.061
Potenza, M. N., Leung, H. C., Blumberg, H. P., Peterson, B. S., Fulbright, R. K., Lacadie, C. M., . . . Gore, J. C. (2003). An FMRI Stroop task study of ventromedial prefrontal cortical function in pathological gamblers. *Am J Psychiatry, 160*(11), 1990-1994. doi:10.1176/appi.ajp.160.11.1990
Qiao, Y., Mei, Y., Du, X., Xie, B., & Shao, Y. (2016). Reduced brain activation in violent adolescents during response inhibition. *Sci Rep, 6*, 21318. doi:10.1038/srep21318
Rae, C. L., Hughes, L. E., Anderson, M. C., & Rowe, J. B. (2015). The prefrontal cortex achieves inhibitory control by facilitating subcortical motor pathway connectivity. *J Neurosci, 35*(2), 786-794. doi:10.1523/jneurosci.3093-13.2015
Ramautar, J. R., Slagter, H. A., Kok, A., & Ridderinkhof, K. R. (2006). Probability effects in the stop-signal paradigm: the insula and the significance of failed inhibition. *Brain Res, 1105*(1), 143-154. doi:10.1016/j.brainres.2006.02.091
Reuter, B., Kaufmann, C., Bender, J., Pinkpank, T., & Kathmann, N. (2010). Distinct neural correlates for volitional generation and inhibition of saccades. *J Cogn Neurosci, 22*(4), 728-738. doi:10.1162/jocn.2009.21235
Roos, L. E., Beauchamp, K. G., Pears, K. C., Fisher, P. A., Berkman, E. T., & Capaldi, D. (2017). Effects of prenatal substance exposure on neurocognitive correlates of inhibitory control success and failure. *Appl Neuropsychol Child, 6*(4), 269-280. doi:10.1080/21622965.2016.1159561
Rosell-Negre, P., Bustamante, J. C., Fuentes-Claramonte, P., Costumero, V., Benabarre, S., & Barros-Loscertales, A. (2014). Reward anticipation enhances brain activation during response inhibition. *Cogn Affect Behav Neurosci, 14*(2), 621-634. doi:10.3758/s13415-014-0292-9
Roth, R. M., Saykin, A. J., Flashman, L. A., Pixley, H. S., West, J. D., & Mamourian, A. C. (2007). Event-related functional magnetic resonance imaging of response inhibition in obsessive-compulsive disorder. *Biol Psychiatry, 62*(8), 901-909. doi:10.1016/j.biopsych.2006.12.007
Rothmayr, C., Sodian, B., Hajak, G., Döhnel, K., Meinhardt, J., & Sommer, M. (2011). Common and distinct neural networks for false-belief reasoning and inhibitory control. *Neuroimage, 56*(3), 1705-1713. doi:10.1016/j.neuroimage.2010.12.052
Rubia, K., Lim, L., Ecker, C., Halari, R., Giampietro, V., Simmons, A., . . . Smith, A. (2013). Effects of age and gender on neural networks of motor response inhibition: from adolescence to mid-adulthood. *Neuroimage, 83*, 690-703. doi:10.1016/j.neuroimage.2013.06.078
Rubia, K., Russell, T., Overmeyer, S., Brammer, M. J., Bullmore, E. T., Sharma, T., . . . Taylor, E. (2001). Mapping motor inhibition: conjunctive brain activations across different versions of go/no-go and stop tasks. *Neuroimage, 13*(2), 250-261. doi:10.1006/nimg.2000.0685
Rubia, K., Smith, A. B., Woolley, J., Nosarti, C., Heyman, I., Taylor, E., & Brammer, M. (2006). Progressive increase of frontostriatal brain activation from childhood to adulthood during event-related tasks of cognitive control. *Hum Brain Mapp, 27*(12), 973-993. doi:10.1002/hbm.20237

Sagaspe, P., Schwartz, S., & Vuilleumier, P. (2011). Fear and stop: a role for the amygdala in motor inhibition by emotional signals. *Neuroimage, 55*(4), 1825-1835. doi:10.1016/j.neuroimage.2011.01.027

Scalzo, F., O'Connor, D. A., Orr, C., Murphy, K., & Hester, R. (2016). Attention Diversion Improves Response Inhibition of Immediate Reward, But Only When it Is Beneficial: An fMRI Study. *Front Hum Neurosci, 10*, 429. doi:10.3389/fnhum.2016.00429

Schel, M. A., Kühn, S., Brass, M., Haggard, P., Ridderinkhof, K. R., & Crone, E. A. (2014a). Neural correlates of intentional and stimulus-driven inhibition: a comparison. *Front Hum Neurosci, 8*, 27. doi:10.3389/fnhum.2014.00027

Schel, M. A., Ridderinkhof, K. R., & Crone, E. A. (2014b). Choosing not to act: neural bases of the development of intentional inhibition. *Dev Cogn Neurosci, 10*, 93-103. doi:10.1016/j.dcn.2014.08.006

Schmitz, N., Rubia, K., Daly, E., Smith, A., Williams, S., & Murphy, D. G. (2006). Neural correlates of executive function in autistic spectrum disorders. *Biol Psychiatry, 59*(1), 7-16. doi:10.1016/j.biopsych.2005.06.007

Schulte, T., Müller-Oehring, E. M., Sullivan, E. V., & Pfefferbaum, A. (2012). Synchrony of corticostriatal-midbrain activation enables normal inhibitory control and conflict processing in recovering alcoholic men. *Biol Psychiatry, 71*(3), 269-278. doi:10.1016/j.biopsych.2011.10.022

Schulz, K. P., Bédard, A. V., Czarnecki, R., & Fan, J. (2011). Preparatory activity and connectivity in dorsal anterior cingulate cortex for cognitive control. *Neuroimage, 57*(1), 242-250. doi:10.1016/j.neuroimage.2011.04.023

Schulz, K. P., Fan, J., Tang, C. Y., Newcorn, J. H., Buchsbaum, M. S., Cheung, A. M., & Halperin, J. M. (2004). Response inhibition in adolescents diagnosed with attention deficit hyperactivity disorder during childhood: an event-related FMRI study. *Am J Psychiatry, 161*(9), 1650-1657. doi:10.1176/appi.ajp.161.9.1650

Sebastian, A., Baldermann, C., Feige, B., Katzev, M., Scheller, E., Hellwig, B., . . . Klöppel, S. (2013a). Differential effects of age on subcomponents of response inhibition. *Neurobiol Aging, 34*(9), 2183-2193. doi:10.1016/j.neurobiolaging.2013.03.013

Sebastian, A., Gerdes, B., Feige, B., Klöppel, S., Lange, T., Philipsen, A., . . . Tüscher, O. (2012). Neural correlates of interference inhibition, action withholding and action cancelation in adult ADHD. *Psychiatry Res, 202*(2), 132-141. doi:10.1016/j.pscychresns.2012.02.010

Sebastian, A., Jung, P., Neuhoff, J., Wibral, M., Fox, P. T., Lieb, K., . . . Mobascher, A. (2016). Dissociable attentional and inhibitory networks of dorsal and ventral areas of the right inferior frontal cortex: a combined task-specific and coordinate-based meta-analytic fMRI study. *Brain Struct Funct, 221*(3), 1635-1651. doi:10.1007/s00429-015-0994-y

Sebastian, A., Pohl, M. F., Klöppel, S., Feige, B., Lange, T., Stahl, C., . . . Tüscher, O. (2013b). Disentangling common and specific neural subprocesses of response inhibition. *Neuroimage, 64*, 601-615. doi:10.1016/j.neuroimage.2012.09.020

Shafritz, K. M., Bregman, J. D., Ikuta, T., & Szeszko, P. R. (2015). Neural systems mediating decision-making and response inhibition for social and nonsocial stimuli in autism. *Prog Neuropsychopharmacol Biol Psychiatry, 60*, 112-120. doi:10.1016/j.pnpbp.2015.03.001

Sharp, D. J., Bonnelle, V., De Boissezon, X., Beckmann, C. F., James, S. G., Patel, M. C., & Mehta, M. A. (2010). Distinct frontal systems for response inhibition, attentional capture, and error

processing. *Proc Natl Acad Sci U S A, 107*(13), 6106-6111. doi:10.1073/pnas.1000175107

Sheridan, M., Kharitonova, M., Martin, R. E., Chatterjee, A., & Gabrieli, J. D. (2014). Neural substrates of the development of cognitive control in children ages 5-10 years. *J Cogn Neurosci, 26*(8), 1840-1850. doi:10.1162/jocn_a_00597

Singh, M. K., Chang, K. D., Mazaika, P., Garrett, A., Adleman, N., Kelley, R., . . . Reiss, A. (2010). Neural correlates of response inhibition in pediatric bipolar disorder. *J Child Adolesc Psychopharmacol, 20*(1), 15-24. doi:10.1089/cap.2009.0004

Sjoerds, Z., van den Brink, W., Beekman, A. T., Penninx, B. W., & Veltman, D. J. (2014). Response inhibition in alcohol-dependent patients and patients with depression/anxiety: a functional magnetic resonance imaging study. *Psychol Med, 44*(8), 1713-1725. doi:10.1017/s0033291713002274

Smith, J. L., Jamadar, S., Provost, A. L., & Michie, P. T. (2013). Motor and non-motor inhibition in the Go/NoGo task: an ERP and fMRI study. *Int J Psychophysiol, 87*(3), 244-253. doi:10.1016/j.ijpsycho.2012.07.185

Song, Y., & Hakoda, Y. (2015). An fMRI study of the functional mechanisms of Stroop/reverse-Stroop effects. *Behav Brain Res, 290*, 187-196. doi:10.1016/j.bbr.2015.04.047

Steele, V. R., Aharoni, E., Munro, G. E., Calhoun, V. D., Nyalakanti, P., Stevens, M. C., . . . Kiehl, K. A. (2013). A large scale (N=102) functional neuroimaging study of response inhibition in a Go/NoGo task. *Behav Brain Res, 256*, 529-536. doi:10.1016/j.bbr.2013.06.001

Stevens, M. C., Kiehl, K. A., Pearlson, G. D., & Calhoun, V. D. (2007). Functional neural networks underlying response inhibition in adolescents and adults. *Behav Brain Res, 181*(1), 12-22. doi:10.1016/j.bbr.2007.03.023

Strakowski, S. M., Adler, C. M., Cerullo, M., Eliassen, J. C., Lamy, M., Fleck, D. E., . . . DelBello, M. P. (2008). Magnetic resonance imaging brain activation in first-episode bipolar mania during a response inhibition task. *Early Interv Psychiatry, 2*(4), 225-233. doi:10.1111/j.1751-7893.2008.00082.x

Suárez-Suárez, S., Doallo, S., Pérez-García, J. M., Corral, M., Rodríguez Holguín, S., & Cadaveira, F. (2020). Response Inhibition and Binge Drinking During Transition to University: An fMRI Study. *Front Psychiatry, 11*, 535. doi:10.3389/fpsyt.2020.00535

Tabu, H., Mima, T., Aso, T., Takahashi, R., & Fukuyama, H. (2011). Functional relevance of pre-supplementary motor areas for the choice to stop during Stop signal task. *Neurosci Res, 70*(3), 277-284. doi:10.1016/j.neures.2011.03.007

Tabu, H., Mima, T., Aso, T., Takahashi, R., & Fukuyama, H. (2012). Common inhibitory prefrontal activation during inhibition of hand and foot responses. *Neuroimage, 59*(4), 3373-3378. doi:10.1016/j.neuroimage.2011.10.092

Tamm, L., Menon, V., & Reiss, A. L. (2002). Maturation of brain function associated with response inhibition. *J Am Acad Child Adolesc Psychiatry, 41*(10), 1231-1238. doi:10.1097/00004583-200210000-00013

Tamm, L., Menon, V., Ringel, J., & Reiss, A. L. (2004). Event-related FMRI evidence of frontotemporal involvement in aberrant response inhibition and task switching in attention-deficit/hyperactivity disorder. *J Am Acad Child Adolesc Psychiatry, 43*(11), 1430-1440. doi:10.1097/01.chi.0000140452.51205.8d

Todd, R. M., Lee, W., Evans, J. W., Lewis, M. D., & Taylor, M. J. (2012). Withholding response in the

face of a smile: age-related differences in prefrontal sensitivity to Nogo cues following happy and angry faces. *Dev Cogn Neurosci, 2*(3), 340-350. doi:10.1016/j.dcn.2012.01.004

Townsend, J. D., Bookheimer, S. Y., Foland-Ross, L. C., Moody, T. D., Eisenberger, N. I., Fischer, J. S., . . . Altshuler, L. L. (2012). Deficits in inferior frontal cortex activation in euthymic bipolar disorder patients during a response inhibition task. *Bipolar Disord, 14*(4), 442-450. doi:10.1111/j.1399-5618.2012.01020.x

Tu, P. C., Yang, T. H., Kuo, W. J., Hsieh, J. C., & Su, T. P. (2006). Neural correlates of antisaccade deficits in schizophrenia, an fMRI study. *J Psychiatr Res, 40*(7), 606-612. doi:10.1016/j.jpsychires.2006.05.012

van der Salm, S. M., van der Meer, J. N., Nederveen, A. J., Veltman, D. J., van Rootselaar, A. F., & Tijssen, M. A. (2013). Functional MRI study of response inhibition in myoclonus dystonia. *Exp Neurol, 247*, 623-629. doi:10.1016/j.expneurol.2013.02.017

Vanderhasselt, M. A., Kühn, S., & De Raedt, R. (2011). Healthy brooders employ more attentional resources when disengaging from the negative: an event-related fMRI study. *Cogn Affect Behav Neurosci, 11*(2), 207-216. doi:10.3758/s13415-011-0022-5

Velanova, K., Wheeler, M. E., & Luna, B. (2009). The maturation of task set-related activation supports late developmental improvements in inhibitory control. *J Neurosci, 29*(40), 12558-12567. doi:10.1523/jneurosci.1579-09.2009

Vercammen, A., Morris, R., Green, M. J., Lenroot, R., Kulkarni, J., Carr, V. J., . . . Weickert, T. W. (2012). Reduced neural activity of the prefrontal cognitive control circuitry during response inhibition to negative words in people with schizophrenia. *J Psychiatry Neurosci, 37*(6), 379-388. doi:10.1503/jpn.110088

Veroude, K., Jolles, J., Croiset, G., & Krabbendam, L. (2013). Changes in neural mechanisms of cognitive control during the transition from late adolescence to young adulthood. *Dev Cogn Neurosci, 5*, 63-70. doi:10.1016/j.dcn.2012.12.002

Vink, M., Zandbelt, B. B., Gladwin, T., Hillegers, M., Hoogendam, J. M., van den Wildenberg, W. P., . . . Kahn, R. S. (2014). Frontostriatal activity and connectivity increase during proactive inhibition across adolescence and early adulthood. *Hum Brain Mapp, 35*(9), 4415-4427. doi:10.1002/hbm.22483

Wager, T. D., Sylvester, C. Y., Lacey, S. C., Nee, D. E., Franklin, M., & Jonides, J. (2005). Common and unique components of response inhibition revealed by fMRI. *Neuroimage, 27*(2), 323-340. doi:10.1016/j.neuroimage.2005.01.054

Wagner, G., Koch, K., Schachtzabel, C., Peikert, G., Schultz, C. C., Reichenbach, J. R., . . . Schlösser, R. G. (2013). Self-referential processing influences functional activation during cognitive control: an fMRI study. *Soc Cogn Affect Neurosci, 8*(7), 828-837. doi:10.1093/scan/nss074

Wagner, G., Sinsel, E., Sobanski, T., Köhler, S., Marinou, V., Mentzel, H. J., . . . Schlösser, R. G. (2006). Cortical inefficiency in patients with unipolar depression: an event-related FMRI study with the Stroop task. *Biol Psychiatry, 59*(10), 958-965. doi:10.1016/j.biopsych.2005.10.025

Walther, S., Goya-Maldonado, R., Stippich, C., Weisbrod, M., & Kaiser, S. (2010). A supramodal network for response inhibition. *Neuroreport, 21*(3), 191-195. doi:10.1097/WNR.0b013e328335640f

Wang, W., Hu, S., Ide, J. S., Zhornitsky, S., Zhang, S., Yu, A. J., & Li, C. R. (2018). Motor Preparation Disrupts Proactive Control in the Stop Signal Task. *Front Hum Neurosci, 12*, 151.

doi:10.3389/fnhum.2018.00151

Wang, Y., Braver, T. S., Yin, S., Hu, X., Wang, X., & Chen, A. (2019). Reward improves response inhibition by enhancing attentional capture. *Soc Cogn Affect Neurosci, 14*(1), 35-45. doi:10.1093/scan/nsy111

Ware, A. L., Infante, M. A., O'Brien, J. W., Tapert, S. F., Jones, K. L., Riley, E. P., & Mattson, S. N. (2015). An fMRI study of behavioral response inhibition in adolescents with and without histories of heavy prenatal alcohol exposure. *Behav Brain Res, 278*, 137-146. doi:10.1016/j.bbr.2014.09.037

Watanabe, J., Sugiura, M., Sato, K., Sato, Y., Maeda, Y., Matsue, Y., . . . Kawashima, R. (2002). The human prefrontal and parietal association cortices are involved in NO-GO performances: an event-related fMRI study. *Neuroimage, 17*(3), 1207-1216. doi:10.1006/nimg.2002.1198

Welander-Vatn, A., Jensen, J., Otnaess, M. K., Agartz, I., Server, A., Melle, I., & Andreassen, O. A. (2013). The neural correlates of cognitive control in bipolar I disorder: an fMRI study of medial frontal cortex activation during a Go/No-go task. *Neurosci Lett, 549*, 51-56. doi:10.1016/j.neulet.2013.06.010

Weywadt, C. R., Kiehl, K. A., & Claus, E. D. (2017). Neural correlates of response inhibition in current and former smokers. *Behav Brain Res, 319*, 207-218. doi:10.1016/j.bbr.2016.11.030

White, C. N., Congdon, E., Mumford, J. A., Karlsgodt, K. H., Sabb, F. W., Freimer, N. B., . . . Poldrack, R. A. (2014). Decomposing decision components in the stop-signal task: a model-based approach to individual differences in inhibitory control. *J Cogn Neurosci, 26*(8), 1601-1614. doi:10.1162/jocn_a_00567

Wingenfeld, K., Rullkoetter, N., Mensebach, C., Beblo, T., Mertens, M., Kreisel, S., . . . Woermann, F. G. (2009). Neural correlates of the individual emotional Stroop in borderline personality disorder. *Psychoneuroendocrinology, 34*(4), 571-586. doi:10.1016/j.psyneuen.2008.10.024

Wittfoth, M., Buck, D., Fahle, M., & Herrmann, M. (2006). Comparison of two Simon tasks: neuronal correlates of conflict resolution based on coherent motion perception. *Neuroimage, 32*(2), 921-929. doi:10.1016/j.neuroimage.2006.03.034

Yang, J., Ye, J., Wang, R., Zhou, K., & Wu, Y. J. (2018). Bilingual Contexts Modulate the Inhibitory Control Network. *Front Psychol, 9*, 395. doi:10.3389/fpsyg.2018.00395

Zandbelt, B. B., & Vink, M. (2010). On the role of the striatum in response inhibition. *PLoS One, 5*(11), e13848. doi:10.1371/journal.pone.0013848

Zhang, J., Hughes, L. E., & Rowe, J. B. (2012a). Selection and inhibition mechanisms for human voluntary action decisions. *Neuroimage, 63*(1), 392-402. doi:10.1016/j.neuroimage.2012.06.058

Zhang, S., Hu, S., Chao, H. H., Luo, X., Farr, O. M., & Li, C. S. (2012b). Cerebral correlates of skin conductance responses in a cognitive task. *Neuroimage, 62*(3), 1489-1498. doi:10.1016/j.neuroimage.2012.05.036

Zheng, D., Oka, T., Bokura, H., & Yamaguchi, S. (2008). The key locus of common response inhibition network for no-go and stop signals. *J Cogn Neurosci, 20*(8), 1434-1442. doi:10.1162/jocn.2008.20100

Zurawska Vel Grajewska, B., Sim, E. J., Hoenig, K., Herrnberger, B., & Kiefer, M. (2011).

Mechanisms underlying flexible adaptation of cognitive control: behavioral and neuroimaging evidence in a flanker task. *Brain Res, 1421*, 52-65. doi:10.1016/j.brainres.2011.09.022
